# Pursuing the elusive biosignature for suicide: a decennial update

**DOI:** 10.1038/s41380-026-03507-5

**Published:** 2026-03-12

**Authors:** Ran Barzilay, Katherin Sudol, Nikolaos P. Daskalakis, Maura B. Dupont, Maria A. Oquendo

**Affiliations:** 1 Department of Psychiatry, Perelman School of Medicine, University of Pennsylvania, Philadelphia, PA USA; 2 Department of Child and Adolescent Psychiatry and Behavioral Sciences, Children’s Hospital of Philadelphia (CHOP), Philadelphia, PA USA; 3 Lifespan Brain Institute of CHOP and Penn Medicine, Philadelphia, PA USA; 4 Department of Psychiatry and Behavioral Sciences, Endeavor Health, Evanston, IL USA; 5 Department of Psychiatry, McLean Hospital, Harvard Medical School, Belmont, MA USA; 6 Stanley Center for Psychiatric Research, Broad Institute of MIT and Harvard, Cambridge, MA USA; 7 Department of Pharmacology, Physiology, & Biophysics, Chobanian and Avedisian School of Medicine at Boston University, Boston, MA USA; 8 Department of Psychiatry, Vagelos College of Physicians and Surgeons, Columbia University, New York State Psychiatric Institute, New York, NY USA

**Keywords:** Neuroscience, Genetics

## Abstract

Suicide is the second leading cause of death for American adolescents and young adults and is transdiagnostic. Suicide risk is impacted by genetic and both distal and proximal environmental factors, particularly stress exposures. This review encompasses the past 10 years of research comparing biological measures between suicide decedents and control decedents and identifies studies focused on stress-related biological pathways, inflammation, neuroplasticity, and the serotonergic system as candidate contributors. Inclusion criteria for studies aimed to maximize confidence that reported biological differences are specific to suicide and independent of confounding psychiatric comorbidity, addressing ambiguity in previous work. The review revealed evidence for alterations in stress-related biological systems and decreased serotonergic tone among suicide decedents. Methodological and conceptual advances over the past decade have driven a shift from hypotheses-driven to data-driven approaches, including genomic, transcriptomic and methylomic analyses. While multi-omic studies have the potential to identify mechanistic molecular targets, to date findings lack interpretability. This review highlights the need for research in larger samples, across multiple brain areas, and in specific cell types to fill a gap in system biology-guided multi-omic studies. Lastly, incorporating poly-environmental stress exposure (exposomic) models in suicide postmortem research may elucidate mechanisms linking environmental stress and biological measures, potentially increasing the reproducibility of postmortem suicide studies.

## Introduction

Suicide is the third leading cause of death globally among those aged 15–29 years [1]. In the United States, suicide is the second leading cause of death for individuals aged 10–14 and 20–34, and the third leading cause for those aged 15–19 [2]. Suicide often presents in the context of environmental stressors including trauma and isolation, yet biological processes are also key [3, 4]. Identifying biological correlates of suicide death may deliver biomarkers and new treatment targets. Of note, suicidal behavior occurs with a range of lethality and of suicidal intent, variables that themselves may be linked to biomarkers [5, 6]. To reduce the variance this may introduce, we focus on the most severe suicidal behavior, suicide [4] and review important inroads in the search for genes and polygenic risk scores to identify suicide risk.

**Fig. 1:**
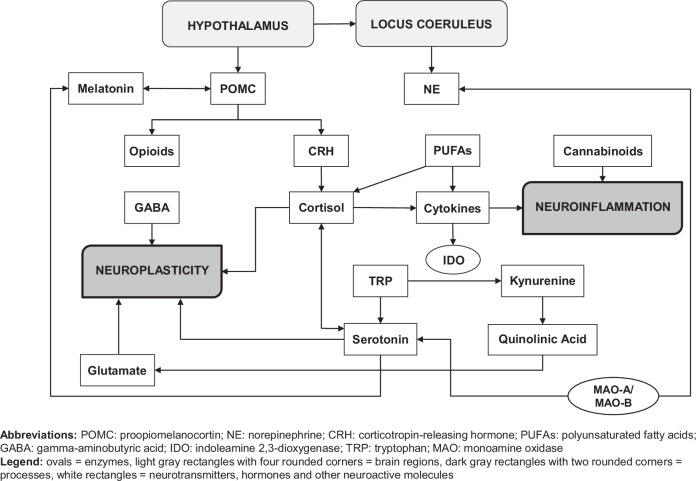
Conceptual Model of Biomarker Relationships in Suicide Risk. A model for relationships between biomarkers studied in relation to suicide.

The neurobiology underlying suicide is profoundly complex, involving a dynamic interplay between stress-related biological pathways, inflammation, neuroplasticity, and serotonergic and other systems. At the core of this complexity lies the hypothalamic-pituitary-adrenal (HPA) axis, orchestrating the body’s response to stress through tightly regulated connections between adrenal cortex and both subcortical and cortical brain regions [7]. Chronic stress can dysregulate the HPA axis, leading to persistent glucocorticoid elevations that affect immune function—promoting a pro-inflammatory state—but also disrupt neuroplastic processes crucial for adaptive brain remodeling [8, 9]. Inflammation, in turn, can impair neuroplasticity and alter serotonergic neurotransmission, both implicated in mood regulation and vulnerability to suicidal behavior [10]. The serotonergic system is itself directly influenced by stress hormones, illustrating the intertwined nature of these systems [11]. The deep interconnection between stress, immune signaling, neuroplasticity, and serotonin function, forms the basis for the biological risk for suicide in a manner that defies simple explanation (Fig. 1 shows depiction).

A decade ago, our literature review summarized findings related to biomarkers associated with suicide [4]. We reported that dysregulation of stress response systems, especially the HPA axis, may have downstream effects on neuroinflammation and neuroplasticity at the level of cells and circuits, leading to abnormalities in these systems in suicide. That review underscored that whether serotonergic abnormalities identified in suicide are independent of stress response abnormalities remained unclear. Here, we review and summarize studies from the last decade presenting biological findings specifically associated with suicide death, independent of comorbid psychiatric conditions.

## Methods

We searched the literature between July 2022 and February 2024 for studies published between January 1, 2014 and December 31, 2023 in PubMed. Detailed search criteria and terms can be found in the Supplementary Materials. Briefly, two different syntaxes combined the term “suicide” with terms across systems previously implicated in suicide (including stress biology, neuroinflammation, neuroplasticity and neurotransmitters) and applied specific logic to focus on original postmortem research, excluding studies of suicide attempters. Three of the authors (RB, KS, MAO) searched the literature within specific biological systems using these syntaxes and screened abstracts to identify papers for full review. We read the papers yielded by the search in full and finalized the set to be included in the review based on agreed-upon criteria. We also reviewed all reference lists of studies identified through the search to locate additional studies. We included peer-reviewed, original, English language, post-mortem research studies that compared biological indices in individuals with a psychiatric diagnosis who died by suicide to the same indices in individuals with that same psychiatric diagnosis who did not die by suicide, which we refer to as non-suicides. Three exceptions to this rule were made. We included studies comparing 1) suicide decedents with heterogeneous psychiatric disorders to non-psychiatric non-suicide controls, referred to as controls, 2) suicide decedents to a heterogenous non-suicide group (including both non-suicides and controls), and 3) suicide decedents to a group for which information about psychiatric disorders was not specified or available. Studies that compared suicide decedents with a homogenous psychiatric diagnosis to non-psychiatric non-suicide controls were excluded due to challenges in interpreting whether differences between the populations are related to suicide or the psychiatric diagnosis. To ensure findings were derived from what we conceived to be minimally powered sample sizes, we excluded papers in which any studied groups (cases or comparison groups) included <20 subjects. These studies (n = 60) are summarized in Supplementary Tables 1-4. We did not include reviews, meta-analyses, book chapters, or case series.

## Results

Suicide is complex, resulting from multiple risk factors [12, 13]. One conceptual model for suicidal behavior is the diathesis-stress model, whereby a person with a vulnerability (the diathesis) experiences stressful life circumstances or a psychiatric illness episode and their risk crosses a critical threshold leading to suicide [14]. This model is supported by data showing that suicide decedents display an overrepresentation of suicides in their families [15] and/or demonstrate substantial exposures to adversity, particularly at young ages [16]. Evidence also supports the critical role of recent life stressors in precipitating suicide [17]. We frame findings linking stress biology to suicide primarily around the HPA axis, acknowledging that the diathesis–stress model likely also applies to other systems that interact closely with the HPA axis, as included in this review.

A total of 129 manuscripts met inclusion criteria. Of these, 69 included at least 20 subjects in every comparison group. Characteristics of each study’s sample and a summary of main findings are in 4 tables categorized based on methodological approaches to enable methodical descriptions of the changes associated with suicide across 1) levels of analysis, from genomics and epigenomics to transcriptomics and proteomics, 2) tissue types (focal brain regions, CSF, blood), and 3) biological systems; and to highlight the potential importance of a multiomics approach to suicide research. Table 1 includes studies examining brain expression of genes and proteins, and their regulation through epigenetic processes. Table 2 includes genetic studies. Table 3 includes studies employing neurochemical and autoradiographic analyses of brain tissue. Table 4 includes studies testing biological changes in post-mortem specimens other than brain tissue.

**Table 1:** Gene and Protein Expression Findings in Suicide Decedents.

System	Author/ Year	Sample •Source: [Bank name]•Toxicology: psychotropics, drugs/alcohol •psychiatric non suicide group •other special characteristics	Brain Region	Gene expression			Protein expression		Epigenetic analysis	Findings	Comments
				Array/other	qPCR	RNA-seq	Western blot (WB)/ELISA	IHC			
**Stress biology**	Pandey 2019 [30]	24 S (6 of them NPCs, the rest with mixed diagnoses of AUD, MDD, adjustment, 24 NS NPCs; Tox: + / Meds: + Source: Brain Collection, Maryland Psychiatric Research Center	PFC (BA9) Hippocampus and Amygdala		+		+			Compared to controls, suicide brains had: (1) increased mRNA levels of CRH in the PFC, in the central amygdaloid nucleus (CeAMY) and in the subiculum. mRNA levels of CRH-Receptor1 (CRHR1) and CRF binding protein (CRH-BP) were significantly decreased in the PFC. No observed changes in the hippocampus of any of the CRH components studied; (2) CRH protein expression was significantly increased and CRHR1, CRHR2 and CRH-BP was significantly decreased in the PFC in S compared to C. There were no changes in the protein expression of CRH components in the hippocampus.	All teenage subjects. Not clear if effects are specific to suicide or relate to the psychiatric disorders in the suicide group. Psychiatric diagnoses determined through a structured interview with a family member/friend
**Stress biology**	Rizavi 2023 [31]	24 S (multiple psychiatric diagnoses), 24 NS NP; Tox: + / Meds: + Source Brain Collection, Maryland Psychiatric Research Center	PFC (BA9) and Hippocampus		+				+	Gene expression analyses of glucocorticoid receptor and FKBP5 revealed suicide cases showed decreased expression in specific GR exon-1 variants in PFC and increased FKBP5 levels in both PFC and hippocampus. There were significant differences in gene expression of DNA methylating and demethylating enzymes. Lower gene expression in PFC was accompanied with greater methylation of the FKPB5 gene promoter region in suicide compared to controls.	All teenage subjects. The suicide cohort consisted of several diagnostic groups, including MDD, AUD/drug abuse, and conduct disorders, with limited power for testing diagnostic specificity.
**Stress biology**	Pandey 2016 [32]	52 S (24 MDD, 16 SCZ, 12 Substance or Conduct), 27 NS (12 MDD, 15 SCZ) and 24 NPCs. Tox: + / Meds: + Source: Brain Collection, Maryland Psychiatric Research Center	PFC (BA9)		+			**+**		The study examined gene and protein expression of SKA2 and found mRNA expression of SKA2 was significantly decreased in the PFC of S relative to NS and NPC; this finding was not driven by any particular diagnosis. SKA2 protein expression was also significantly decreased in the PFC of S relative to NS and NPC.	Large sample with 52 S. Males > females. Various psychiatric diagnoses included.
**Stress biology**	Kouter 2023 [33]	25 S, 28 NS Tox: + / Meds: + Source: Institute of Forensic Medicine, University of Ljubljana, Slovenia	Hippocampus, Insula, amygdala, PFC (BA 46)		+				**+**	The study used targeted methylation analyses to compare methylation of 8 genes (NR3C1 (coding for corticosterone receptor), SLC6A4, HTR1A, TPH2, SKA2, MAOA, GABRA1, and NRIP3). In the insula, compared to controls, suicide was associated with hypermethylation of NR3C1, HTR1A, and SKA2; and hypomethylation of GABRA1 and NRIP3, which was also hypomethylated in the hippocampus and amygdala of suicide compared to control. Gene expression levels of NR3C1 and SLC6A4 were lower in the hippocampus of suicide compared to control.	All male subjects. No report of diagnoses in either S or NS. Methylation analyses were also done in blood (see Table 4).
**Stress biology**	Underwood 2023 [36]	26 S (13 with ELA and 13 without) and 26 NS NPCs (13 with ELA and 13 without); Tox: + / Meds: + Source: not specified	DLPFC (B9), ACC (BA24)				+			Expression levels of CRH, CRH binding protein, GR, FKBP5 and BDNF did not differ between S and NS in both brain regions. For BDNF, there was an interaction between suicide and ELA in ACC; among S, BDNF of S+ELA was higher than S-ELA, while among NS, BDNF of NS+ELA was lower than NS-ELA. This interaction was not observed for DLPFC.	Psychiatric diagnoses of S are unknown. Negative tox for drugs and meds for all donors. Dysregulated HPA axis is related to suicide but not ELA
**Stress biology**	Lutz 2017 [37]	52 S (27 MDD-S with child abuse and 25 MDD-S no child abuse) and 26 NS NP. Tox: NA / Meds: NA Source: Douglas-Bell Canada Brain Bank.	Anterior cingulate cortex			**+**			+	The study applied genome wide DNA methylation and gene expression analyses using reduced representation bisulfite sequencing and RNA-sequencing, respectively. A history of child abuse was associated with cell type–specific changes in DNA methylation of oligodendrocyte genes LINGO3 and POU3F1 and a global impairment of the myelin-related transcriptional program. Effects were absent in MDD-S no child abuse.	Large sample with 51 S. Psychological autopsies were performed.
**Neuroinflammation**	Wang 2018 [41]	Cohort 1: 21 MDD-S, 22 S without MDD, 16 NS NPCs; Tox: + / Meds: + Source: Quebec Suicide Brain Bank Cohort 2: 14 MDD-S, 12 MDD-NS, 12 NS NPCs; Tox: + / Meds: + Source: Brain Collection, Maryland Psychiatric Research Center	DLPFC		+					TNF-a gene expression was significantly higher DLPFC of individuals who died by suicide, regardless of psychiatric diagnosis. Expression of miR-19a-3p, which regulates TNF-a, was upregulated specifically in individuals who died by suicide. Expression of TRBP significantly lower in the dorsolateral prefrontal cortex in suicide subjects.	Study conducted in two cohorts.
**Neuroinflammation**	Clark 2016 [42]	25 MDD-S, 20 MDD-NS, 36 NS NPCs; Tox: NA / Meds: NA Source: NIMH section of Psychopathology.	VLPFC (BA45 and BA47).		**+**					No differences between S and NS in gene expression of interferon, TNF-alpha, IL-13, IL-33 and CCL2; and no evidence for alteration in expression of genes related to the kynurenic pathway in S.	Donors with MDD had lower levels of IFN-gamma and TNF-alpha than NPCs. Study also tested kynurenine metabolism (Table 3).
**Neuroinflammation**	Zheng 2023 [44]	34 SCZ-S, 80 SCZ-NS, 37 Bipolar-S, 41 Bipolar-NS, 48 MDD-S, 39 MDD-NS, 85 NS NPCs. Tox: + / Meds: + Source: SMRI	DLPFC (BA9)				**+**			Study evaluated association of CMV infection (CMV IgG+) with suicide, mood disorders and with inflammatory markers. Among those with a psychiatric disorder SZ, (BD, MDD grouped together), CMV + samples had an OR =2.1 of S, relative to CMV – samples. Post hoc comparisons by dx not significant.	Large sample with 119 S. Study also investigated CMV related neuroinflammation but results in relation to suicide were not reported.
**Neuroinflammation**	Punzi 2019 [50]	65 violent suicide, 46 non-violent suicide, 78 NS with mixed diagnoses. Tox: NA / Meds: NA Source: Lieber Institute for Brain Development brain repository	DLPFC BA9 and BA46			+				Study aimed to confirm the prior postmortem association of the long intergenic noncoding RNA LINC01268 with violent suicide compared to non-violent suicide and to NS. RNA-seq data was used for Weighted Gene Co-expression Network Analysis (WGCNA) revealing a LINC01268-related network containing genes involved in peripheral immunological responses. LINC01268 has the strongest connectivity with P2RY13, largely unknown purinergic receptor in peripheral immune system and in brain.	Large sample with 111 S. Sample included only White individuals. The relationship between suicide and the lincRNA was first explored in a diagnostically mixed sample (SCZ, Bipolar, MDD). The comparison between S and NS was evaluated in patients only.
**Neuroinflammation**	Cabrera- Mendoza 2020 [51]	48 S (22 with MDD, 5 Bipolar, 4 personality disorders), 27 NS (2 with personality disorders); Tox: + / Meds: NA Source: Institute of Forensic Sciences (INCIFO) in Mexico City, Mexico and University of Texas Health Science Center at Houston Brain Collection	DLPFC (BA9)	+						Microarray identified differentially expressed genes between suicides and controls of each sex: 1,729 genes in females and 1,997 genes in males. Female-exclusive suicide genes were related to cell proliferation and immune response. Meanwhile, male-exclusive suicide genes were associated to DNA binding and ribonucleic protein complex. Sex-independent suicide genes showed enrichment in mitochondrial and vesicular functions.	Large sample with 48 S. Study focused on sex differences. In suicide group, 38 males and 10 females, in control group, 20 males and 7 females. Not clear if findings are specific to suicide or to psychiatric diagnoses that were prevalent in suicides. Case definition based on coroner’s records.
**Neuroinflammation**	Punzi 2022 [52]	77 violent suicide, 50 non-violent suicide, 99 NS with MDD, BD, SCZ diagnoses. 103 NS NPC. Tox: NA / Meds: NA Source: Lieber Institute RNA-seq data set (DEF) and postmortem collection (GRS and GO).	DLPFC BA46, BA9			**+**				RNA-seq data was used to identify differentially expressed genes (DEGs) and WGCNA tested for gene coexpression modules and module interactions associated with violent suicide. FDR<0.05. Gene Ontology (GO) Consortium toolkit applied. NV-S patients compared with NS patients yielded 44 DEGs (21 down and 23 up in NV-S), and no significant GO enrichment for these genes. VS patients compared to NS patients yielded 189 DEGs (67 down and 122 up in VS patients); no enrichment for down-regulated DEGs, up-regulated DEGs showed enrichment for biological and molecular processes involving the G protein-coupled purinergic nucleotide receptor signaling pathway. Violent suicide transcriptomic pattern was divergent from other patient groups and more similar to NS NPCs. Violent suicide genomic risk scores were low for their diagnoses and for suicide attempt, but high for IQ. DEGs in violent suicide implicate purinergic signaling in microglia. WGCNA showed DEGs were coexpressed in a context of mitochondrial metabolic activation unique to violent suicide. Nil GO findings except for DEGs up-regulated in violent suicide.	Large sample with 127 S.
**Neuroplasticity**	Youssef 2018 [59]	37 S and 53 NS. 45 had MDD and 45 did not; 32 had ELA. Tox: NA / Meds: NA Source: not specified	Caudal & rostral brainstem; ACC (BA24) & DLPFC (BA9)				+			The study examined BDNF protein levels finding lower BDNF in ACC in S+ELA, S no ELA, NS+ELA compared to NS no ELA; Lower BDNF in ACC and caudal brainstem of MDD vs non-MDD. No difference in dlPFC BDNF in S compared to NS	Undetermined number had alcohol in blood. Psychological autopsy.
**Neuroplasticity**	Ropret 2021 [61]	22 S (various psychiatric diagnoses) and 20 NS NPCs Tox: + / Meds: + Source: Institute of Forensic Medicine, University of Ljubljana, Slovenia	BA9 and hippocampus		+				**+**	The study examined methylation and expression BDNF in two brain regions and in the blood. There was no difference in BDNF methylation between the S and NS in brain samples, with lower BDNF methylation in blood of S vs. NS. In BA9, S had higher expression of BDNF transcript NM_170731.4 than NS, with no differences in BDNF expression in hippocampus. Blood BDNF expression levels could not be quantified.	Caucasian male subject sample. Significant difference in age (NPC>S). One NS had history of SCZ; one was on sertraline. Study also included analyses of blood, see Table 4.
**Neuroplasticity**	Maussion 2014 [64]	Gene expression was assessed in 33 S (mixed diagnoses) and 28 NPCs. Methylation analyses in 11 S and 13 NS (diagnoses unknown). Tox: + / Meds: NA Source: Quebec Suicide Brain Bank	DLPFC (BA 8/9)		**+**				**+**	This study evaluated methylation levels of the TrkB-T1 gene using a custom-built microarray, which has lower expression in brain tissue of suicides. Results show hypermethylation in the frontal cortex of suicide completers in the TrkB-T1 3′UTR region compared to controls, and correlation of methylation with lower mRNA expression levels.	Diagnoses in NS are not noted. Methylation studies only in low TrkB-T1 expression S (n=11); C-no Dx unselected for TrkB-T1
**Neuroplasticity**	Zarrilli 2014 [65]	31 S, 21 NS; Tox: NA / Meds: NA Source: Institute of Forensic Medicine, University of Ljubljana	Wernicke׳s brain tissue		**+**					Expression of TrkB was lower in S compared to controls (1.534 versus 2.165, *P*=0.031). This study also examined polymorphisms (SNPs) of NTRK2 gene and identified 2 out of 17 variants with statistically significant differences for both genotypic and allelic frequencies between suicide and controls (See Table 2).	All Caucasian donors without history of psychiatric disorders.
**Neurotransmitter system: Cannabinoid**	Erdozain 2015 [100]	22 S (11 AUD) and 22 NS (11 AUD). Tox: + / Med: + Source: Basque Institute of Legal Medicine, Spain	DLPFC (BA9)		+		+			This study tested CB1 gene and protein expression, affinity (saturation assays) and functionality (binding assays), as well as evaluation of other endocannabinoid elements. No CB1 receptor gene expression differences were observed across groups. In AUD subjects, there was an increase in CB1 receptor protein in suicide vs. non-suicide samples, with no changes in other endocannabinoid elements.	Study focused on AUD effects. Toxicology of psychotropic drugs and ethanol was conducted. No specification if cannabis was assessed.
**Neurotransmitter system: Cannabinoid**	Tao 2020 [101]	35 S + SCZ, 113 NS + SCZ. Tox: + / Med: + Postmortem human brain tissue multisite collection including banks at NIMH, NICHD and Lieber Institute.	DLPFC		+					Study evaluated gene expression of CB1 receptor gene (CNR1) and found that, compared to schizophrenia NS, the expression of CNR1 is significantly upregulated in the PFC of schizophrenia S.	Study focused on CB1 receptor gene and methylation. Data comparing S to NS is available only for SCZ subject and only in DLPFC. Analysis did not adjust for cannabis toxicology, associated with increased CNR1 gene expression.
**Neurotransmitter system: GABA**	Davis 2016 [89]	176 SCZ patients (35 S), 61 BD patients (40 S), 138 MDD patients (88 S), and 364 NPC. Tox: + / Med: + Source: NIMH Clinical Brain Disorders Branch	dlPFC		**+**					Study examined GAD2 transcripts and found that expression of the full-length GAD2 transcript was associated with S across diagnoses. SCZ-S group had higher expression of GAD2 full transcript in dlPFC than SCZ-NS group.	Large sample of 163 S. Only commented on SCZ group; unclear if others not analyzed independently or if nonsignificant.
**Neurotransmitter system: GABA**	Tao 2018 [90]	DLPFC cohort: 181 SCZ PTs (36S), 69 BD PTs (45S), 146 MDD patients (90S) and 340 NPC. Hippocampus cohort: 128 SCZ PTs (24S), 295 NPCs. Tox: + / Med: + Source: NIMH Clinical Brain Disorders Branch.	DLPFC (BA9 / BA46), hippocampus		**+**					Study examined GAD1 transcripts and found that SCZ-S had higher expression of GAD1 full-length transcript in the dlPFC compared to SCZ-NS; other GAD1 transcripts in the dlPFC did not associate with suicide. There was no association between suicide and GAD1 transcript expression in hippocampus.	DLPFC large sample with 171 S.
**Neurotransmitter system: Glutamate**	Garcia-Gutierrez 2023 [62]	28 S and 26 NS both without DX. with negative Tox; negative Med. Source: Institute of Legal Medicine of Alicante, Spain	DLPFC, hippocampus and amygdala.		**+**			**+**		Assessed gene expression of mGluR5, and its scaffolding proteins Homer1a, and p11 in DLPFC, AMY, and HIP. S had less p11 gene expression in DLPFC, AMY and HIP. S had no difference in mGluR5 or Homer1a gene expression in DLPFC but increased mGluR5 and Homer1a in AMY and HIP. S had less BDNF gene expression in HIP. **Immunohistochemistry in Dentate Gyrus of HIP**, S had less type 1 anti-vesicular glutamate transporters (VGluT1) VGAT-ir and more type 1 anti-vesicular GABA transporters (VGAT1) than C.	alterations in mGluR5, Homer1a, p11, BDNF and excitatory/ inhibitory balance in corticolimbic brain areas of S.
**Neurotransmitter system: Glutamate**	Powers 2020 [87]	51 MDD-S, 28 MDD-NS, and 32 NPCs Tox + / Meds: + Source: NIMH Clinical Brain Disorders Branch	dlPFC		**+**					The study of glutamate transporter expression found females MDD-S, dlPFC expression of EAAT2, VGLUT1, and VGLUT2 levels were higher than in NPCs, but not compared to MDD-NS, with no difference in expression levels in males or between groups. In males, but not females, those who died by a violent suicide method had reduced EAAT1 and EAAT2 expression levels in dlPFC.	Large sample with 51 S.
**Neurotransmitter system: Opioid**	Lutz 2015 [94]	60 S and 34 NS control for Anterior insula; 55 S and 27 NS control for mediodorsal thalamus; 54 S and 26 NS for dACC; Tox: + / Meds: NA Source: Douglas-Bell Canada Brain Bank.	Anterior insula, mediodorsal thalamus and dACC		+					Evaluation of nociceptin/orphanin FQ peptide (N/OFQ) and Nociceptin Opioid-like Peptide (NOP) gene expression revealed decreased N/OFQ levels by 18% in the dACC of suicide subjects independent of current depressive or substance disorders at the time of death. No such effects were observed in other brain areas.	Large sample with 60 S. Mood disorder rate was 84% in S and 15% in NS group. Substance use disorder rate was 45% in S and 15% in NS group.
**Neurotransmitter system: Opioid**	Lutz 2018 [95]	26 S + childhood abuse, 30 S – childhood abuse, 26 NS NP. Tox: NA / Med: NA. Source: Douglas-Bell Canada Brain Bank.	ACC, Mediodorsal thalamus, Anterior insula.		+				+	The study tested opioid Kappa opioid receptor gene expression and targeted DNA methylation using bisulfite sequencing. Differences in Kappa expression were only observed in Anterior Insula. History of child abuse was associated in the anterior insula with downregulation of the kappa receptor expression and methylation, which were not observed for S without child abuse.	The aim of this work was to identify effects of child abuse, not suicide, hence analyses do not focus on suicide effects, rather controlled for it to specifically link effects of child abuse to biological phenotypes. Abuse interview that assesses various dimensions of childhood experience, including sexual and physical abuse, as well as neglect.
**Neurotransmitter system: Opioid**	Gaine 2019 [96]	23 BD-S, 27 BD-NS, 31NPCs Tox: NA / Med: NA. Source: SMRI	PFC (BA46)						**+**	The study evaluated differential methylation throughout the genome across study groups. Comparison of BD-S to BD-NS revealed 330 DMR (183 hypermethylated and 147 hypomethylated in BD suicide subjects), with a DNA methylation difference ranging from 6 to 19%. Pathway analysis of the genes identified in the differential methylation comparison between BD-S to BD-NS revealed the opioid signaling pathway as the top canonical pathway, while this pathway was not among the top pathways when comparing BD to NPC or BD-S to NPCs.	
**Neurotransmitter system: Serotonin**	Di Narzo 2014 [69]	Cohort 1: 22 S (23% MDD); 29 NS NPCs. Source: Brain Collection of Dr Yasmin Hurd (Mount Sinai, NY, NY) Tox: + /Med: + Cohort 2: MDD-S (N=10); MDD-NS (N=24) C-no DX (N=31) Source: U Pittsburgh brain collection Tox: NA / Meds: +	Cohort 1: OFC (BA 11); Cohort 2: subgenual ACC (BA 25)			**+**			**+**	The study evaluated 5-HT2CR RNA editing using massive parallel sequencing of PCR-amplified fragments identifying 32 5-HT2CR mRNA isoforms. S express more ABCD mRNA variant, encoding a hypoactive 5-HT2CR Isoform than NS with same DX.	Donors were not drug or med free in Cohort 2
**Neurotransmitter system: Serotonin**	Ramos-Rosales 2022 [70]	20 S and 20 NS Tox: NA / Meds: NA Source: Forensic Medical Services of the General Fiscally of the State of Durango, Mexico	PFC, ventromedial nucleus of hypothalamus		+					This study examined expression levels of 5HTR2A and MAO-A genes finding higher 5HTR2A expression in PFC and higher MAO-A expression in hypothalamus of S compares to NS.	Mostly male sample with some individuals < age 18. Most S were by violent methods. A few NS with AUD/drug abuse/dependence; unclear about other psychopathology.
**Neurotransmitter system: Serotonin**	Bach 2016 [74]	23 S and 26 NPC, stratified by smoking status (Sm vs NSm) Tox: - /Med: + Source: Not specified	DRN			**+**				This study used in situ hybridization to examine TPH mRNA expression and found S non-smokers had higher TPH2 mRNA expression than S smokers, NPC smokers and NPC non-smokers. There were no differences in TPH2 expression between S smoking and NS smoking. In DRN subnuclei, the difference between S smokers and S non-smokers was only significant in the ventrolateral DRN.	Predominantly male sample. S with higher incidence of smoking, and more heavy smokers. Various psychopathology included.
**Neurotransmitter system: Other**	Bristow 2021 [78]	79 MDD PTs (51 S and 28 NS) and 32 NPCs. Tox: NA / Meds: + Source: NIMH Clinical Brain Disorders Branch	dlPFC		+					This study tested transcriptional differences in monoaminergic genes including SERT, NET, DAT, VMAT, PMAT, TPH1, TPH 2. There were no differences in monoamine gene transcription between depressed S and NS as a whole, and when separated by sex.	Large sample with 51 S. No information about toxicology.
**Other**	Miguel-Hidalgo 2014 [102]	30 S (6 AUD -MDD, 18 no AUD + MDD, 6 AUD + MDD), 22 NS (10 AUD -MDD, 5 no AUD + MDD, 7 AUD + MDD); Tox: NA / Meds: NA Source: Cuyahoga County Coroner's Office in Cleveland, Ohio.	oPFC (BA 47)					+		Among psychiatric subjects, no difference in connexin43 levels or immunostaining was found between suicides and non-suicides.	Study focused AUD and MDD effects and found reduced connexin43 expression in both conditions with no effects for suicide. Diagnoses were made based on informants and medical records.
**Other**	Yoshida 2019 [103]	61 S, 128 controls; Tox: NA / Meds: NA. conducted in the Source Department of Legal Medicine, University of Toyama, Toyama, Japan	Locus ceruleus, raphe nucleus, entorhinal cortex					+		The study evaluated Alzheimer disease related tau and amyloid beta pathology in non-demented young people. Amyloid beta and tau pathology was not associated with suicide. The study also evaluated association of Apolipoprotein E (APOE) genotype and found no clear significant association between APOE e4 allele and suicide history.	Large sample with 61 S. All brains were from young people under 40. Psychiatric evaluations were not available for many cases, hence effects cannot be specifically related to suicide.
**Other**	Kurtulus Dereli 2018 [104]	21 S (7 of which had MDD diagnosis), 21 controls. Tox: NA / Meds: NA Source: Pamukkale University, Department of Forensic Medicine, Finland	Pineal gland					+		Lower levels of acetylserotonin 0-methyltransferase (ASMT), the final enzyme in melatonin synthesis pathway, were observed in brains of suicide descendants.	Study sample was young (18-40) due to calcification of pineal gland in older ages. Study also tested blood and urine samples.
**Other**	Udawela 2017 [105]	Cohort 1a: 16 SCZ-S, 28 SCZ-NS, and 26 NS NPCs Cohort 1b: 12 SCZ-S, 26 SCZ-NS and 20 NS NPCs. Cohort 2: 13 MDD-S, 2 MDD-NS, 5 Bipolar-S, 10 Bipolar-NS 15, 9 S without diagnoses, and 14 NS NPCs. Tox: NA / Meds: + Source: Australian Brain Bank Network at Florey Institute of Neuroscience and Mental Health	BA9 and BA24		+		+			This study examined phospholipase C beta (PLCB; variants a & b) gene and protein expression. Within Schizophrenia donors across combining all cohorts, there was greater mRNA expression in S vs NS decedents in BA9, with no differences in protein levels of PLCB in BA9 or BA 24.	
**Other**	Cabrera 2019 [112]	43 S (23 + SUD and 20 -SUD), 23 NS (9 + SUD and 14 -SUD); Tox: + / Med: - Source: Institute of Forensic Sciences (INCIFO) in Mexico City, Mexico	DLPFC (BA9)	+						Microarray revealed 222 differentially expressed genes, predominately enriched in cell proliferation in the comparison between suicides with and without SUD. When comparing the transcriptome of suicides with SUD to non-suicide cases with SUD, results identified 550 differentially expressed genes, mainly enriched in oxidative phosphorylation. Differentially expressed genes (1,417) between suicides and non-suicides without SUD were detected, mostly related to mitochondrial function.	Per authors, this is the first transcriptome analysis of suicide in a Latino population (Mexico City).
**Other**	Policicchio 2020 [114]	25 MDD-S, 28 NS NPCs; Tox: + / Meds: NA Source: Douglas-Bell Canada Brain Bank.	PFC (BA 11) and cerebellum (BA 25)						+	This study evaluated DNA methylation changes in the brain by utilizing previously published and unpublished methylome datasets. Analyses revealed altered DNA methylation at several genetic loci in suicide cases compared to controls in both examined brain regions with suicide-associated differentially methylated positions enriched among functional pathways relevant to psychiatric phenotypes and suicidality, including nervous system development (PFC) and regulation of long-term synaptic depression (cerebellum). Functional analyses of gene expression in the PFC showed that changes in methylation can influence gene expression using a dual luciferase assay, but specific target genes were not identified.	Additional analyses were done only in cerebellum tissue comparing 50 S to 80 NS with psychiatric conditions. This analysis showed that cerebellum suicide-associated findings were largely independent of comorbid psychiatric disorders.

**Abbreviations**: Activating transcription factor (ATF), Anterior Cingulate Cortex (ACC), adrenoreceptor alpha 1a/2a, beta 1 (ADRA1A, ADRA2A, ADRB1), alcohol use disorder (AUD), aldehyde dehydrogenase-1 family, member L1 (ALDH1L1), androgen receptor (AR), antidepressant (AD), arginine vasopressin receptor-1a (AVP1a), benzodiazepines (BZD), brain-derived neurotrophic factor (BDNF), Brain Enriched Guanylate Kinase Associated (BEGAIN), Brodman area (BA), calcium/calmodulin-dependent protein kinase II alpha (CAMK2A), CAMP-response element-binding protein (CREB), Cannabinoid receptor 1 (CB1), Cannabinoid receptor 2 (CB2), catechol-O-methyltransferase (COMT), C/EBP homologous protein (CHOP), cellular RA binding protein 1, 2 (CRABP1, 2), chemokine (C-X3-C motif) receptor 1 (CX3CR1), cluster of differentiation 68 (CD68), control (c), coronin1A CORO1A, corticotropin-releasing hormone (CRH), CRH binding protein (CRHBP), 2',3'-Cyclic-nucleotide 3'-phosphodiesterase (CNPase), cytochrome P450, dentate gyrus (DG), delta like canonical Notch ligand (DLL), differentially methylated region (DMR); dopamine receptor D1 and 2 (DRD1, DRD2), dopamine transporter (DAT), dorsal anterior cingulate cortex (dACC), dorsal raphe nucleus (DRN), dorsolateral prefrontal cortex (DLPFC), early life adversity (ELA), estrogen receptor a/b (ERa/b), family 26, A1, B1, C1 (CYP26A1, B1, C1), excitatory amino acid transporter (EAAT), FK506-binding protein 51 (FKBP5), protein-coupled receptor 30 (GPR39), G protein-coupled receptor 55 (GPR55), Glial fibrillary acidic protein (GFAP), glucocorticoid receptor (GR), glucose-regulated protein (GRP), glutamate ammonia ligase (GLUL), glutamic acid decarboxylase (GAD), glutamate transporter 1 (GLT1), Glycogen Synthase Kinase 3 Beta (GSK3b), glutamine synthetase (GS), GPM6A, GPM6B, GRIK3, GRIN2B, GRM2, Hardy-Weinberg Equilibrium (HWE), heat shock protein 70 (HSP 70) and HSP 90, human leukocyte antigen-DRA (HLA-DRA), IBA1-immunoreactive (IBA1-IR), Indoleamine 2,3-dioxygenase-1 (Ido-1), immunoglobulin (Ig), interferon (IFN), interleukin-1b (IL1b), interleukin 6 (IL-6), ionized calcium-binding adapter molecule-1 (IBA1), Kyoto Encyclopedia of Genes and Genomes (KEGG), Major Depressive Disorder (MDD), psychotropic medications (Med), metabotropic glutamate receptor (mGlu), microRNA (miR), mineralocorticoid receptor (MR), minor allele frequency (MAF), monoamine oxidase A (MAOA), monoamine oxidase B (MAOB), myelin-associated glycoprotein (MAG), myelin basic protein (MBP), myelin oligodendrocyte glycoprotein (MOG), Myelin Proteolipid Protein (PLP), nerve growth factor (NGF), nerve growth factor receptor (NGFR), neural progenitor cells (NPC); neurotrophic tyrosine receptor kinase (NTRK, also known as tropomyosin-related kinases, Trk), neurotrophin-3 (NT-3), Neurogenic locus notch homolog (NOTCH), neurotrophin-4/5 (NT-4/5), neutral amino acid transporter (ASCT), Nitrous Oxide Synthase: NOS1, NOS2, NOS3, NOS1-interacting DHHC domain-containing protein with dendritic mRNA (NIDD), non-suicide (NS), not assessed (NA), non-psychiatric controls (NPC), norepinephrine transporter (NET), nuclear receptor subfamily 3 group C member 1 (NR3C1), Nucleolar organizing regions (NORs), oligodendrocyte-lineage (OL), oligodendrocyte transcription factor 2 (OLIG2), orbitofrontal cortex (OFC), plasma membrane monoamine transporter (PMAT), polygenic risk score (PGR); polymerase chain reaction (PCR), Prefrontal Cortex (PFC), purinergic receptor 12 (P2RY12), quantitative real-time polymerase chain reaction (qPCR), RARa, b, g and retinoid X receptor a, b, g (RXRa, b, g), receiver operating characteristic (ROC), retinaldehyde dehydrogenase 1,2,3 (RALDH 1,2,3), S100 calcium binding protein b (S100b), serotonin receptor (5HTR), schizophrenia (SCZ), serotonin receptor 1A and 2A (5-HT1A and 5-HT2A), serotonin transporter (SERT), Shared Genomic Segments (SGS), single nucleotide polymorphism (SNP), sodium-coupled neutral amino acid transporter (SNAT), spindle and kinetochore associated complex subunit 2 (SKA2), substance use disorder (SUD), suicide (S), suicide attempter (SA), toll like receptor (TLR), toxicology (Tox), translocator protein (TSPO), triggering receptor expressed on myeloid cells 2 (TREM2), transactivation Response RNA binding protein (TRBP), Tryptophan 2,3-dioxygenase (TDO2), tryptophan hydroxylase (TPH), tumor necrosis factor-a (TNFa), urocortin 3 (UCN3), ventrolateral prefrontal cortex (VLPFC), vesicular glutamate transporter (VGLUT), vesicular monoamine transporter (VMAT), Xbox binding protein (XBP-1).

**Table 2:** Genetic Findings in Suicide Decedents.

System	Author/Year	Sample	Genes/Loci	Sample	Findings	Comments re:
**Stress biology**	Fudalej 2015 [34]	563 S, 475 NS; + Tox; NA Med. Cases from Department of Forensic Medicine, Medical University of Warsaw, Poland. Suicide based on postmortem examination results.	FKBP5 polymorphisms rs1360780 and rs3800373	Blood samples collected during autopsy	Significant association between the high-induction rs3800373 C allele and suicide (OR = 1.36).	The control group were live people representing the population of central Poland, with no psychiatric screening.
**Stress biology**	Park 2016 [35]	182 S, 161 NS; All subjects were cancer patients from Seoul.	Functional Bcl-1 polymorphism of (rs41423247), the neuron-specific glucocorticoid receptor (NR3C1) gene.	formalin-fixed paraffin-embedded tissue samples of patients.	No association with Bcl-1 polymorphism was found when comparing S to NS. Within S, GG genotype of Bcl-1 polymorphism were at increased risk of early suicide (within one year of cancer diagnosis) when compared to those carrying the CC genotype (OR 3.8).	The control group were alive by the end of 13-year follow-up.
**Neuroinflammation**	Kaushik 2023 [40]	218 S, 226 NS from Indian populations.	Association study of polymorphisms in TNF-α promoter region.	Blood samples.	GG genotype of SNP TNF-α(□ 308 G>A) and TT genotype of SNP TNF-α-(850 C>T) were significantly higher in suicide	MH history was low in suicides (9%) and 0 in controls, likely due to MH diagnoses stigma in India.
**Neuroinflammation**	Shimmyo 2017 [43]	602 S, 728 NS of Japanese descent. suicide determination based on medicolegal examination and police investigation.	Association study of two functional polymorphisms on MIF gene promoter and suicide.	Peripheral blood.	No significant differences in allele or genotype frequency distributions of the MIF promoter polymorphisms between suicides and controls.	Healthy controls were alive.
**Neuroplasticity, BDNF**	Zarrilli 2014 [65]	55 S, 86 NS; Subjects collected from the Institute of Forensic Medicine, University of Ljubljana	Used PCR to examine polymorphisms (SNPs) of NTRK2 gene.	Brain	Of 17 SNPs examined, two (i.e. rs10868235 C/T and rs1867283 A/G) showed statistically significant differences for both genotypic and allelic frequencies between suicide and controls. Study also tested expression level of TrkB (see Table 1).	All Caucasian donors without history of psychiatric disorders.
**Neurotransmitters, Adrenergic system**	Rivero 2016 [79]	236 S, 280 NS (of which n=187 without psychiatric diagnosis). Suicide determined by a medical examination in the Basque Institute of Legal Medicine, Spain.	Evaluation of the polymorphism a2CDel322-325-AR that confers a2C-adrenoceptor (a2C-AR) loss-of-function	Brain (prefrontal cortex)	The frequency of a2CDel322-325-AR in suicide (9%) and non-suicide subjects (11%) was similar.	The study was performed in a homogenous set of Caucasian subjects from European origin.
**Neurotransmitters, Adrenergic system**	Hasegawa 2023 [80]	1007 S, 884 NS of Japanese descent. Suicide based on the medicolegal examinations and police investigations.	Association study of the variable number of tandem repeats (VNTR) in MAOA gene promoter using fluorescence-based PCR assays.	Peripheral blood.	Neither the genotype-based associations nor allele/haplotype frequencies of the two VNTRs were significantly associated with suicide.	
**Neurotransmitter, GABAergic, Glutamatergic**	Yin, Pantazatos et al. 2016 [91]	121 S and 88 NS Source: Unspecified Medical Examiner’s Office, UK Brain Expression Consortium	Examination of 119 SNPs in 24 candidate genes in the glutamatergic and GABAergic systems.	Brain	There was no relationship between genotype and suicide.	NS included those with and without various psychiatric diagnoses.
**Neurotransmitter, Other**	Čugura 2018 [81]	77 complex S, 406 simple S, and 289 controls Source: Institute of Forensic Medicine, Faculty of Medicine, University of Ljubljana, Slovenia	Examination of MAOA and MAOB polymorphisms (rs3027407, rs909525, rs1137070, rs1799836)	Whole blood, liver and heart tissue samples	Male complex suicides had different allele distributions of two MAOA polymorphisms relative to simple suicides and controls. Compared to victims of complex suicide, male victims of simple suicide were more often carriers of MAOA alleles that are, according to literature, associated with higher levels of impulsivity and anger. No differences in allele distribution were found in females and no difference in allele distribution was found for MAO B.	Complex suicides used more than one method. Unclear if control group included psychiatric diagnoses.
**Neurotransmitter, Other**	Lombardo 2015 [83]	34 S, 24 NS; Subjects collected from the Institute of Forensic Medicine, University of Ljubljana	CGH array	Brain	Two of the S cases had genomic rearrangements present in genes previously related to psychiatric risk. One subject showed an amplification of an area of at least 59 bp at Xp11.3 (chrX:43,516,819-43,516,878, hg19) involving the intron 1 of *monoamino-oxidase A (MAOA)* gene; a second subject harbored a duplication of about 2.5 Mb located at 22q11.21 (chr22:18,894,835-21,464,119, hg19) involving several genes among which was *catechol-O-methyl transferase (COMT)* gene.	All Caucasian donors. Psychiatric diagnoses not described.
**Neurotransmitters, Serotonergic**	Rahikainen 2017 [73]	349 S (137 violent and 212 non-violent) and 284 NS citalopram users Tox: +/ Meds: +Source: Department of Forensic Medicine, University of Helsinki	Examined the rs25531 polymorphism of 5-HTTLPR, MAOA-uVNTR, and 5-HTR2B Q20* using RT-PCR	Blood	Male citalopram users with low functioning s/s genotype of 5HTTLPR/rs25531 are at increased risk to die by violent suicide. This was not observed for female subjects. There were no differences in MAOA-uVNTR, and 5-HTR2B Q20* allele frequencies between S and NS.	Four samples excluded. No information about illicit substance toxicology (only alcohol discussed). No information about psychopathology.
**Neurotransmitters, Serotonergic**	Uršič 2018 [82]	266 S and 191 NS Tox: + / Meds: NA Source: Institute of Forensic Medicine, Faculty of Medicine University of Ljubljana	Used PCR to examine the allele frequencies of a MAO-uVNTR polymorphism	Blood	In males, statistically significant differences in allele frequencies observed between the following groups: 1) NS and non-violent S, 2) NS without alcohol dependence and S, 3) NS without alcohol dependence and S without alcohol dependence. S more likely to have 3R allele (associated with lower transcription rate relative to other alleles). No differences in females.	Predominantly male sample. NS group included those with alcohol dependence. No information about psychopathology.
Other (GWAS)	Galfalvy 2015 [107]	Sample 1: MDD-S (n=75), Other dx- S (n=43), No Dx-S (n=3) Sample 2 (controls pooled from two sites): C-NS live-no DX (n=794), C-NS live-any DX (n=302), C-NS deceased no DX (n=80), C-NS deceased any DX (n=57) Sample 3: MDD-S (n=38), Other or unk DX-S (n=143), No DX-S (n=15)	Genome-wide association study of suicides.	N/A	No SNPs were associated with suicide at 10-8	Samples from the US, Canada for suicides and controls. Additional controls from Germany
Other (GWAS)	Otsuka 2019 [108]	Two independent datasets totaling 746 S and 14,049 NS of Japanese descent.	Genome-wide association study of suicides.	N/A	No genome-wide significant SNP for suicide. There was a significant SNP-based heritability (35–48%; P < 0.001) for suicide by genomic restricted maximum-likelihood analysis and a shared genetic risk between two datasets by polygenic risk score analysis.	
Other (GWAS)	Han 2023 [109]	986 S and 415 controls from the Utah Suicide Genetic Risk Study (USGRS)	Genomic association analysis prioritizing brain expression quantitative trait loci (eQTLs) within regulatory regions in suicide deaths from the Utah Suicide Genetic Risk Study (USGRS)		One significant eQTL locus, rs926308 showed genome wide significant association with suicide (*p* = 3.24e−06). This result was also replicated through rigorous investigation within two independent suicide cohorts. The rs926308-*T* is associated with lower expression of *RFPL3S*, a gene important for neocortex development and implicated in arousal.	Donors of European ancestry.
Other	Coon 2020 [111]	Sample 1 43 Utah families (7-9 generations) heavily loaded for S (n=215) Sample 2 Utah S (n= 1300)	Shared Genomic Segments study to identify rare genetic variants in large families with high suicide liability.	Blood samples	Sample 1: 207 genes confer suicide risk-- 18 previously associated with S. Sample 2: Putatively functional SNPs in target SGS genes with elevated MAF: SP110 Gene transcription; immune deficiency; AGBL2 ATP/GTP binding; brain structure and function; SUCLA2 Mitochondrial protein, energy to synapse; APH1B Transmembrane protein linked to Alzheimer’s and Parkinson’s diseases	Multiple diagnoses.

**Abbreviations**: 5-HTR, serotonin receptor; ACC, anterior cingulate cortex; EFEMP1, gene encoding EGF-containing fibulin-like extracellular matrix protein 1; FKBP5, FK506-binding protein 51; MAOA, monoamine oxidase A; MDD, major depression disorder; Meds, medications; MH, mental health; MIF, Macrophage migration inhibitory factor; NA, not assessed; NP, non-psychiatric; NR3C1, nuclear receptor subfamily 3 group C member 1; NS, non-suicide; NTRK2, neurotrophic tyrosine receptor kinase 2; OFC, orbitofrontal cortex; OR, odds ratio; PFC, prefrontal cortex; SCZ, schizophrenia; sg, subgenual; S, suicide; SA, suicide attempt; SNP, single nucleotide polymorphism; TNF, tumor necrosis factor.

**Table 3:** Neurochemical, Autoradiographic, and Other Findings in Suicide Decedents.

System	Author/year	Sample • Source: [Bank name] • Toxicology: psychotropics, drugs/alcohol • psychiatric non suicide group • other special characteristics	Brain Region	Method	Findings	Comments re:
**Neuroinflammation**	Clark 2016 [42]	25 MDD-S, 20 MDD-NS, 36 NS NPCs; Tox: NA / Meds: - Source: NIMH section of Psychopathology.	VLPFC (BA45 and BA47).	Kynurenine pathway compounds were determined with HPLC and gas chromatography/ mass Spectrometry. qPCR was used to assess gene expression enzymes that catalyze kynurenine and of cytokines.	No differences among depressed suicides compared to depressed non suicides. Reduced kynurenine metabolism in depression versus control groups with lower conversion of tryptophan to kynurenine, lower gene expression of Ido-1 and TDO-2, and lower IFN-gamma and TNF-alpha levels (see Table 1).	Information on the use of antidepressants and other psychotropic medications was insufficient for statistical comparisons.
**Fatty acids**	Hamazaki 2015 [54]	20 S (of which 13 MDD, 3 SCZ, 4 BIP), 40 NS from a sample of 2 MDD, 12 SCZ, 11 BIP and 15 NP controls. Tox: NA / Meds: NA Source: Victorian Brain Bank Network (VBBN), Florey Institute for Neuroscience and Mental Health.	Prefrontal cortex (BA8).	Examination of n-3 polyunsaturated fatty acid (PUFA) levels using thin layer and gas chromatography.	No significant differences were found in any individual PUFAs between suicide subjects and non-suicide subjects. However, significant decreases were seen in total saturated fatty acids in suicide subjects (-1.4%).	Not clear if effects are specific to suicide as most suicides had psychiatric disorders and 3/8 of controls did not.
**Fatty acids**	Hamazaki 2016 [55]	45 S (of which 44 with SCZ and 1 without psychiatric diagnoses) and 143 NS (of which 51 with SCZ and 92 without psychiatric diagnoses). Tox: NA / Meds: + Source: Victorian Brain Bank Network (VBBN), Florey Institute for Neuroscience and Mental Health.	frontal cortex (BA8).	Examination of n-3 PUFA levels using thin layer and gas chromatography.	Linoleic acid, arachidonic acid, and docosapentaenoic acid were significantly lower in suicide than in non-suicide. In contrast, erucic acid (22:1 n-9), docosatetraenoic acid, and the ratio of n-6/n-3 were higher in the suicide group than in the non-suicide group.	Not clear if effects are specific to suicide as most suicides had psychiatric disorders and 2/3 of controls did not.
**Fatty acids**	Hamazaki 2017 [56]	20 S (of which 13 MDD, 3 SCZ, 4 BIP), 40 NS from a sample of 2 MDD, 12 SCZ, 11 BIP and 15 NP controls. Tox: NA / Meds: NA Source: Victorian Brain Bank Network (VBBN), Florey Institute for Neuroscience and Mental Health.	Corpus Callosum.	Examination of n-3 polyunsaturated fatty acid (PUFA) levels using thin layer and gas chromatography.	No significant differences in any PUFAs between suicides and non-suicide cases regardless of psychiatric disorder. Slightly higher levels of the monosaturated fatty acid, oleic acid in suicides.	Not clear if effects are specific to suicide as most suicide cases had psychiatric disorders and 3/8 of controls did not.
**Neurotransmitters:** **Serotonergic system**	Underwood 2018 [11]	83 S and 149 NS Various diagnoses Tox: + / Meds: - Source: not specified	PFC	Used quantitative autoradiography to map SERT, 5-HT1a, and 5-HT2a receptor binding	SERT: Lower binding in S across all brain regions, although it was attributable to MDD diagnosis. 5-HT1a: Greater binding in S group independent of MDD diagnosis. AUD associated with more 5-HT1a binding but only in suicides. 5-HT2a: Greater binding in suicides when MDD and AUD included in the model.	NS control group included subjects with and without psychiatric diagnoses. Psychological autopsy available for 61S and 106 NS. Significant findings were limited to subgroup with psychological autopsy.
**Neurotransmitters:** **Serotonergic system**	Odagaki 2021 [71]	BD (n=20, 14S), MDD (n=20, 17S), SCZ (n=20, 10S), and NS NPCs (n=20). Tox: + / Meds: + Source: Basque Institute of Legal Medicine, Bilbao, Spain	PFC	Used [35S]GTPcS binding/ immunoprecipitation assay to determine the functional activation of Gaq/11 proteins coupled to 5-HT2A receptors	S within each diagnosis did not differ in half-maximal effect, maximum percent increase, or slope factor of 5-HT induced [35S]GTPcS binding to Gaq/11 relative to NS and HC. This did not change when S across diagnoses were combined and compared to NS and HC.	
**Neurotransmitters:** **Serotonergic system**	Krzyzanowska, Steiner, Karnecki et al. 2016 [75]	27 S and 30 NS, unknown diagnoses in both groups. Tox: most had negative blood alcohol / Med: NA Source: Department of Forensic Medicine, Medical University of Gdańsk.	DRN	The transcriptional activity of ribosomal DNA (rDNA) in DRN neurons was evaluated by the AgNOR silver staining method in paraffin embedded brain tissue.	AgNOR staining was decreased in S vs C suggesting serotonergic neurons are producing fewer axonal endings and smaller neuronal bodies.	Dx for S is unknown, most decedents had no blood alcohol detected.
**Neurotransmitters:** **Serotonergic system**	Krzyżanowska, Steiner, Brisch et al. 2016 [76]	24 S and 20 NS with various psychiatric diagnoses (MDD, BD or SCZ), 28 NPCs. Tox: NA / Med: assessed in health records. Source: Department of Forensic Medicine, Medical University of Gdańsk.	DRN	The transcriptional activity of ribosomal DNA (rDNA) in DRN neurons was evaluated by the AgNOR silver staining method in paraffin embedded brain tissue.	AgNOR staining was decreased in S vs NS and C suggesting the finding in S is independent of psych dx	No SUD, <50% had psychotropics in 90 days prior to death based on health records.

**Abbreviations**: 5-HT, serotonin; 5-HTR, serotonin receptor; ACC, anterior cingulate cortex; AUD, alcohol use disorder; BA, Brodman area; D1, dopamine 1 receptor; D2, dopamine 2 receptor; DAT, dopamine transporter; DRN, dorsal raphe nucleus; GFAP, Glial fibrillary acidic protein; HIAA, hydroxyindoleacetic acid; Ido-1, Indoleamine 2,3-dioxygenase-1; IFN, interferon; MDD, major depression disorder; Meds, psychotropic medications; NA, not assessed; NMDAR, NMDA receptor; NPC, non-psychiatric control; NS, non-suicide; ; OFC, orbitofrontal cortex; PFC, prefrontal cortex; PUFA, polyunsaturated fatty acid; rDNA, ribosomal DNA; S, suicide; SERT, serotonin transporter; SCZ, schizophrenia; TDO2, Tryptophan 2,3-dioxygenase; TDP-43, Transactive response DNA binding protein of 43 kDa; TNF, tumor necrosis factor; Tox, toxicology; VLPFC, ventrolateral prefrontal cortex.

**Table 4:** Findings in Non-Brain Biological Substrate of Suicide Decedents.

System	Author/year	Sample	Biological Substrate	Method	Findings	Comments
**Stress biology**	Laugesen 2021 [27]	12,028 S, 140,278 NS controls matched using risk-set sampling, birth year and sex. Sample derived from a Danish Registry.	N/A	Case-control registry study of glucocorticoid use and risk of suicide in a Danish population.	Oral glucocorticoid initiation was associated with suicide in a dose-dependent manner, with findings of a 7-fold increased risk in cancer patients and a 2-fold increased risk in patients treated for other medical conditions.	
**Stress biology**	Ragnarsson 2019 [28]	N=502 patients with Cushing’s disease of which 6 died by suicide. Sample derived from a Swedish Patient Registry.	N/A	Comparison of the observed number of deaths among patients with Cushing’s disease with the expected number from the general population.	Cushing’s disease is associated with excess death by suicide than expected.	77% women in the sample.
**Stress biology**	Chaurasia 2023 [29]	100 S, 20 NS Tox: NA / Meds: NA Sample derived from a medical center in Bhopal, India.	Adrenal glands	Comparison of adrenal volume, weight and relative adrenal weight (weight of adrenal per squared height of subject) between suicide decedents and controls.	Compared to controls, weight and relative weight of the adrenal glands was greater among suicide decedents. No differences were observed in adrenal glands’ volume between groups.	Cases with alcohol detected at autopsy and with known history of taking antidepressants were excluded.
**Inflammation**	Dickerson 2018 [45]	1292 individuals (733 SCZ, 483 BD, 76 MDD) followed for 8.2 years with 16 confirmed deaths by suicide.	Blood	Associations of blood serological markers (Ig G antibodies to herpes simplex virus type 1 (HSV-1), CMV, Epstein-Barr virus (EBV), human herpes virus type 6 (HHV-6), and the protozoan Toxoplasma gondii) with suicide in a population of people with psychiatric disorders.	S had higher levels of IgG to CMV with an apparent dose effect.	75% of S had a SUD
**Inflammation**	Russell 2021 [46]	N=462,747 (of which 687 S cases) and N=359,849 (of which 605 S cases) participants with information on WBC count and CRP, respectively. Source: the Taiwan MJ cohort.	Blood	Associations study of WBC count and CRP levels with suicide.	There was an association of suicide with WBC count (adjusted hazard ratio [aHR] = 1.13 per 1 standard deviation increase of log-transformed WBC. The association was driven by the highest quintile of WBC count (aHR = 1.39; reference: the lowest quintile). No association between CRP and suicide was found.	
**Inflammation**	Batty 2018 [47]	106 643 men (of which 1010 suicide cases) and 312 884 women (of which 1019 suicide cases). Cohort obtained from the Korean Cancer Prevention Study.	Blood	Associations of WBC count with suicide death in adults.	In women, those in the second WBC quartile and higher experienced around 30% increased risk of death (HR 1.35; 95% CI: 1.11–1.64). No such association was observed in men.	
**Inflammation**	Batty 2016 [48]	39, 349 participants followed for mean 8.6 years with 26 suicide cases. Data were pooled from a series of independent, geographically representative health surveys conducted in the UK that were later linked with national cause of death registers.	Blood	Associations of blood CRP levels with later death by suicide. CRP was categorized as high (>3mg/L), intermediate (1-3mg/L) and low (<1mg/L).	People in the highest inflammation group were 4-times more likely to die by suicide relative to those in the lowest group, adjusting for age, sex, smoking, and socioeconomic factors.	
**Inflammation**	Bultink 2021 [49]	SLE patients (n=4,356) of which n=32 died by suicide and matched controls (n=21845). Cohort obtained from a UK clinical research dataset representing 8% of the UK population.	N/A	Population-based cohort study to assess whether SLE is associated with increased suicide risk compared to non-SLE control subjects using Cox proportional hazards models.	Death attributable to suicide were all significantly increased in SLE patients compared with age- and sex-matched subjects.	Finding was not specific to suicide with increased risk for other non-suicide death in SLE group.
**Neuroplasticity**	Liu 2023 [60]	30 S, 25 live controls with no data on diagnoses Tox: -/Meds: NA	Blood	Enzyme-linked immunosorbent assays (ELISA) used to detect BDNF, proBDNF, BDNF/proBDNF, Trk-b, GDNF, and TPH2 levels	Compared to controls, S blood samples had greater TPH and proBDNF levels, lower GDNF levels and a lower and BDNF/proBDNF ratio, with no differences in BDNF or Trk-b levels.	S: 50% no known dx, 50% had either MDD or SZ Live C had no physical illness or brain injury; no PMI data.
**Neuroplasticity**	Ropret 2021 [61]	22 S and 20 NS Tox: +/Meds: + Source: Institute of Forensic Medicine, Faculty of Medicine, University of Ljubljana, Slovenia	Blood	Examined DNA methylation with next-generation sequencing of bisulfite-converted DNA, and mRNA expression levels with PCR	S blood samples showed reduced DNA methylation in a BDNF region upstream of exon 1. Study also included analyses of brain tissue, see Table 1.	Caucasian male subject sample. Significant difference in age (HC>S). One NS had SCZ, another on sertraline. S with various diagnoses.
**Neurotransmitters, Serotonergic system and stress biology**	Kouter 2023 [33]	25 S, 28 NS Tox: + / Meds: + Source: local brain collection.	Blood	Examined targeted methylation of eight genes linked with suicide: NR3C1, SLC6A4, HTR1A, TPH2, SKA2, MAOA, GABRA1, and NRIP3.	Compared to controls, suicide was associated with hypomethylation of HTR1A and GABRA1 and hypermethylation of SKA2 and MAO-A.	All male subjects. No report of diagnoses in either S or NS. Methylation analyses were also done in brain (see Table 1).
**Neurotransmitters, Serotonergic system**	Dogan 2016 [77]	32 S and 56 NS Tox: NA / Meds: NA Source: Selcuk University Faculty of Medicine	CSF	Examined CSF levels of S100B protein and 5-HT using ELISA	CSF S100B protein levels were higher in S than NS. CSF 5-HT levels were significantly lower in S than in NS.	Sample contained 8 teenagers. Sample predominantly male. No information about psychopathology.
**Other**	Otsuka 2017 [106]	For peripheral blood samples, 508 S and 535 NS. For brain tissue, 20 S and 25 NS. All subjects were of Japanese descent.	Blood and prefrontal cortex	Quantification of telomere length and of mitochondrial DNA copy number	In peripheral blood, suicide samples had significantly shorter TL and higher mtDNAcn of peripheral bloods with sex/age dependent differences (shorter TL was more remarkably in female/young suicides; higher mtDNAcn more so in male/elderly suicides). In brain tissue, suicide cases had shorter TL and lower mtDNAcn.	Controls of blood analyses were derived from healthy living NPCs. Diagnoses not clear for brain donors.

**Abbreviations**: 5HT, serotonin; CRP, C-reactive Protein; HIAA, hydroxyindoleacetic acid; HVA, Homovanillic acid; mtDNA, mitochondrial DNA; NS, non-suicide; S100b, calcium binding protein b; suicide; SLE, systemic lupus erythematosus; TL, telomere length; WBC, white blood cells.

## Stress biology and suicide

The HPA Axis is a major physiological pathway for stress response; its activation by stress may alter immune responses and lead to inflammation [18, 19]. Inflammation, in turn may dysregulate neuronal pruning [20] and serotonergic transmission [21], further disrupting neural circuitry. Upon receiving stress signals, the paraventricular nucleus of hypothalamus (PVN) releases corticotrophin releasing hormone (CRH), which stimulates proopiomelanocortin (POMC) production from the anterior pituitary leading to adrenocorticotropic hormone (ACTH) release into the bloodstream, affecting cortisol release by the adrenal cortex [22]. Glucocorticoid receptors (GR) are intracellular receptors mediating downstream cortisol effects. In tissues with expression of high-affinity mineralocorticoid receptors (MR), like hippocampus, there is a balance between MR activation during basal states and GR activation during stress states [23]. Two key molecules regulate GR function: FKBP5, a co-chaperone protein that forms a complex with GR, decreases GR affinity to glucocorticoids, decreasing overall GR signaling [24]; and SKA2, a protein that interacts with GR increasing its activation [25]. These molecular GR regulators not only influence cellular responses to glucocorticoids but also impact neural circuitry. Alterations in GR signaling, modulated by FKBP5 and SKA2, can affect synaptic plasticity and the connectivity of stress-responsive brain regions such as hippocampus, PFC, and amygdala, thereby shaping behavioral and cognitive responses to stress [26].

Most [27–34], but not all [35, 36] studies examining changes in neurobiological components of the stress response found associations with suicide. Two conditions that increase cortisol levels, exogenous glucocorticoid administration [27] and Cushing disease [28], were both associated with suicide risk. Further, one study reported greater adrenal gland weight in suicide decedents compared to controls [29]. In brain, most studies reported significant differences in HPA axis indices in suicide decedents compared to non-suicides or controls, though changes were not consistent across brain regions. For example, a study of PFC, hippocampus, subiculum and amygdala of suicides compared to controls revealed that CRH mRNA levels and protein expression were increased in PFC (BA 9), central amygdaloid nucleus and subiculum of suicides; mRNA levels and protein expression of CRH-R1 and CRH-BP, but not CRH-R2, were decreased in PFC and amygdala, perhaps due to down-regulation, with changes in subiculum being more varied. In contrast, no changes in hippocampal CRH components were detected [30]. Another study from the same group reported decreased expression of specific GR exon-1 variants in PFC and hippocampus in suicides and a strong correlation of DNA methylation changes with gene expression in PFC [31]. Alterations were also observed in proteins modulating GRs, whereby, compared to non-suicides or controls, suicides showed decreased gene and protein expression of SKA2 in PFC, decreasing GR activation [32]; increased gene expression of FKBP5 in PFC, decreasing GR affinity [31]; hypermethylation of GR and SKA2 genes in insula, decreasing GR activity [33]; and decreased FKBP5 methylation in PFC also decreasing GR affinity [31]. However, a single study reported no differences in protein expression of CRH, GR, and FKBP5 in dorsolateral PFC (DLPFC) and anterior cingulate cortex (ACC) [36]. The discrepancy between PFC findings may relate to the possibility that differences in gene expression observed between suicide cases and controls [31] do not always translate to differences in protein levels, which were not observed between groups [36], due to post-transcriptional and translational regulation. Additionally, differences in sample characteristics—specifically, the inclusion of adolescents [31] and a cohort enriched with early life adversity [36] —could also contribute to divergent findings. Analyses of methylation profiles in blood revealed hypermethylation of the SKA2 gene, but no differences in GR gene methylation among suicides compared to controls [33]. Together, these studies suggest GR function downregulation. One study using genome-wide methylation analyses found that among depressed suicide decedents, child abuse was associated with cell type–specific changes in DNA methylation of oligodendrocyte genes [37]. Genetic studies found an association with suicide of a polymorphism that upregulates FKBP5 gene expression [34], and no association of a functional polymorphism (Bcl-1) of the GR gene [35]. Thus, studies support the association of altered stress biology with suicide, with most studies documenting the role of elevated peripheral cortisol and decreased GR activity (decreased SKA2, increased FKBP5, and variable methylation of glucocorticoid signaling genes), particularly in PFC. Other brain regions remain understudied in suicide.

## Neuroinflammation and suicide

Converging evidence implicates inflammation in suicide [38, 39]. First, stress biology is related to inflammation through the HPA axis and its end-product, cortisol, a potent suppressor of inflammation in the short term, though pro-inflammatory if chronically elevated. Second, production of serotonin, the neurotransmitter most robustly associated with suicide, is curtailed by inflammation [4]. Below, we summarize evidence for immune alterations in suicide decedents’ brains and systemic inflammation’s association with suicide.

Studies of individual cytokines in suicides [40–42] have yielded inconsistent evidence for alterations in tumor necrosis factor alpha (TNF-α) levels. TNF-α, a pro-inflammatory cytokine activating indoleamine 2,3-dioxygenase (IDO), an enzyme catabolizing tryptophan to produce kynurenine, shunts tryptophan away from serotonin synthesis [4]. For example, in a targeted genetic study (i.e., candidate gene) examining polymorphisms in the basal promoter region that influences TNF-α expression, suicide was associated with alterations in two sites, one leading to increased and one to decreased TNF-α expression [40]. In contrast, another targeted genetic study examined promoter polymorphisms of the macrophage migration inhibitory factor, a cytokine promoting TNF-α production, and found no significant differences in allele, genotype, or haplotype frequencies between suicides and controls [43]. One study reported increased TNF-α gene expression in DLPFC of suicide decedents compared to controls [41], yet another found no such differences, nor differences interferon, IL-13 and IL-33 gene expression in ventrolateral PFC [42]. Differences in the PFC regions examined may account for inconsistent findings regarding differences in TNF-α expression levels across the two studies. No studies about other cytokine dysregulation in suicide decedents’ brains met inclusion criteria.

Among studies focused on individual pathogens, one suggests an association of cytomegalovirus (CMV) infection with suicide. Brains with positive CMV IgG were more than two-fold as likely to belong to suicide decedents than CMV IgG-negative brains [44]. Another study found a dose–response link between CMV seropositivity in blood and suicide, with no similar association for other viruses [45].

Additional converging evidence linking systemic inflammation to suicide arises from studies examining peripheral indicators of inflammation. Suicide was associated with higher white blood cell counts [46], with one study indicating this occurs only in women [47], and greater C-reactive protein levels [48], a finding not replicated in another study [46]. Moreover, systemic lupus erythematosus, a multisystem autoimmune disorder, is associated with increased suicide risk compared with the general population [49].

Lastly, we identified three data-driven studies showing differences between suicide decedents and controls in DLPFC gene expression-related signaling pathways involved in immune response [50–52]. Two studies from the same laboratory applied RNA-seq and Weighted Gene Co-expression Network Analysis (WGCNA) to study gene expression in violent and non-violent suicides compared to controls and suggest that differences in immune gene networks, such as those involved in purinergic signaling [53], may be specific to violent suicide [50, 52]. The third was a microarray study examining sex-specific gene expression in DLPFC that identified differential expression of genes related to cell proliferation and immune response in female, but not male suicides and genes associated with DNA binding and ribonucleic protein complex in male, but not female suicides [51]. These approaches highlight the potential of unbiased data-driven whole genome expression studies to identify individual relevant genes, gene networks (i.e., genes with coordinated/correlated expression), and pathways that are dysregulated in suicide decedents’ brains. However, heterogeneity in these studies’ findings makes it difficult to draw definitive conclusions. More work is needed to translate findings obtained with data‑driven methods into interpretable biological mechanisms.

To summarize, some data support an association between inflammation and suicide, both centrally and peripherally, with little data on specific mechanisms underpinning this association, although there is support for the role of TNF-α and CMV infection. It is possible that the link between suicide and CMV reflects secondary infections resulting from reduced immune function. As well, individuals with comorbid mental and physical illnesses may have compromised immunity, increasing vulnerability to infections, making findings about these specific factors far from definitive.

## Polyunsaturated fatty acids and suicide

Polyunsaturated fatty acids (PUFA) are abundant in brain with either pro-inflammatory (omega-6) or anti-inflammatory (omega-3) effects. Prior work did not show conclusive evidence for altered PUFA levels in suicide [4]. Three studies examined alterations in fatty acid levels in suicide decedents. In frontal cortex, there were no differences in PUFA levels, but lower levels of total saturated fatty acids were found in suicide decedents compared to controls [54]. Another study found lower linoleic acid (n-6), arachidonic acid (n-6), and docosapentaenoic acid (n-3) levels, higher erucic acid (n-9) and docosatetraenoic acid (n-6) levels, and a higher n-6/n-3 fatty acid ratio in suicide decedents’ PFC compared to controls [55]. In corpus callosum, greater oleic acid levels (n-9) were seen in suicide decedents compared with controls, while total PUFA levels were unchanged [56]. Together, the evidence for fatty acids dysregulation in PFC and corpus callosum is difficult to interpret, while other brain regions remain unstudied.

## Neuroplasticity

Brain derived neurotrophic factor (BDNF), a growth factor involved in neural development, neuronal survival and synaptic plasticity [57], has been extensively studied due to its role in stress vulnerability [58]. BDNF and its receptor, tropomyosin receptor kinase-B (TRK-B) are the most studied in relation to neuroplasticity and implicated in suicide’s pathogenesis. One study showed BDNF levels to be higher in suicide decedents’ ACC with and without early life adversity compared to controls without early life adversity [59], and in a study from the same laboratory, higher in suicide decedents with early life adversity compared with suicides without life adversity, suggesting differences were due to early life adversity. However, among controls, BDNF levels were lower in those with early life adversity compared to those without it, making interpretation more complex [36]. Another study observed no alterations in BDNF or TRK-B blood levels in suicide decedents [60]. Additional findings are also inconsistent, with one study reporting greater BDNF gene expression in suicide decedents’ PFC but not hippocampus [61], and another reporting lower BDNF gene expression in suicide decedents’ hippocampus relative to controls [62]. Differing results may be due to study population heterogeneity, as both studies compared suicide cases and controls across mixed diagnostic groups rather than within specific diagnoses, potentially confounding results given substantial BDNF level variability across different psychiatric conditions [63].

As for BDNF gene methylation, no differences were observed in brain, but lower methylation was reported in suicide decedents’ blood compared to controls [61]. Hypermethylation of TRK-B was reported in suicides’ DLPFC relative to controls, which was correlated with lower gene expression [64]; this was also observed in Wernicke’s area [65].

Thus, little evidence supports alterations of BDNF gene expression or methylation in suicide decedents’ brains. Early life adversity may impact BDNF expression in suicide decedents through changes in stress signaling and may be related to the reported differences in BDNF methylation patterns in the periphery. In contrast, reported differences in TRK-B receptor methylation and gene expression in suicides’ brains were somewhat more compelling, which may reflect deficits in brain’s stress response as disruption of TRK-B can impair dendritic spine stability in PFC and alter HPA activity [66].

## Neurotransmitters

The most extensively studied neurotransmitter system in suicide is the serotonergic system. We also identified studies examining glutamatergic, GABAergic, opioid, and cannabinoid system alterations.

### Serotonergic system

Serotonin (5-hydroxytryptamine [HT]) is a monoamine neurotransmitter key to various high level brain functions including mood and cognition [67]. Importantly, the serotonergic system is central to the brain’s capacity to cope with stress [68].

Studies of brain serotonin receptors noted changes in RNA editing of 5-HT2C suggestive of hypoactivation [69], higher expression of 5HT2A [70], and greater binding in 5-HT1A and 5-HT2A receptors [11] in PFC of suicide decedents compared to controls. A single study reported no difference in 5-HT2A receptor activation in PFC of suicide decedents and controls [71]. One study found hypomethylation of the 5HT1A gene in blood among suicide decedents relative to controls [33]. Notably, such hypomethylation was associated with greater 5HT1A gene expression in blood [72].

Differences between suicide and non-suicide decedents were also observed in serotonin transporter function. In a study of serotonergic system-related candidate genes, suicide was associated with a polymorphism of the serotonin transporter (5HTTLPR) linked to lower 5HTT functioning [73]. Lower serotonin transporter binding in PFC and ACC was found in suicide decedents compared to controls [11]. In dorsal raphe nucleus (DRN), greater expression of tryptophan hydroxylase (TPH), the rate-limiting enzyme in serotonin synthesis, was observed in non-smoking suicide decedents compared to smoking suicide decedents and controls. However, no differences in TPH expression were reported among suicide decedent smokers relative to controls, suggesting higher TPH expression was related to smoking, not suicide [74]. Additional research reported lower ribosomal DNA activation in suicides’ DRN relative to controls [75, 76]. Lastly, one study reported lower cerebrospinal fluid serotonin levels in suicide decedents compared to controls [77]. To summarize, multiple studies report alterations in the serotonergic system with lower serotonin levels, hypoactivation of the serotonin transporter and 5HT2C receptor, and higher expression of 5HT1A and 2 A receptors, suggesting hypoactivation of serotonergic transmission in suicide, with most studies conducted in PFC. A key consideration is that some of these changes, for example in receptor expression, may be compensatory rather than primary.

### Other monoaminergic neurotransmitter studies

Prior evidence suggests norepinephrine and, to a lesser extent, dopamine neurotransmission disruption in suicide [4], together with altered expression of monoamine oxidase-A (MAO-A), an enzyme that metabolizes serotonin, dopamine and norepinephrine.

We identified one study assessing expression levels of various monoamine transporter genes (serotonin, norepinephrine, dopamine) in DLPFC finding no differences between suicides and controls [78]. Additionally, we identified five candidate gene studies examining associations of polymorphisms in monoaminergic genes with suicide. One study of the α2C-adrenoceptor loss-of-function polymorphism found no association with suicide [79]. Of the remaining four genetic association studies, all focused on MAO-A, two found no association between MAO-A polymorphisms and suicide [73, 80], and two reported associations with suicide only in males [81, 82]. Of note, one of the latter studies additionally found no associations of MAO-B polymorphisms with suicide [81]. Lastly, a single study reported hypermethylation of the MAO-A gene in blood [33]. One study employed microarray-based Comparative Genomic Hybridization (CGH) and identified genomic rearrangements in genes relevant to monoaminergic neurotransmitter metabolism in two of 34 brains of suicide decedents included in the study compared to controls [83]. One subject had a rearrangement in intron 1 of the MAO-A gene and the other subject in the catechol-O-methyltransferase (COMT) gene, which metabolizes catecholamines.

Thus, little systematic evidence supports associations of adrenergic or dopaminergic system alterations with suicide, but inconsistent evidence suggests MAO-A polymorphisms may be associated with suicide in males. Moreover, many of the studies are candidate gene studies, which have historically shown limited reproducibility [84].

### Glutamatergic and GABAergic systems

Glutamate is a major excitatory neurotransmitter critical for learning, memory and neuroplasticity and glutamate signaling may be affected by environmental stress [85]. Despite the growing interest in glutamatergic transmission’s role in suicide spurred by the anti-suicidal effects of ketamine, a glutamate N-methyl-D-aspartate (NMDA) receptor antagonist [86], only two studies of the glutamatergic system in suicide met inclusion criteria. The first investigated glutamate transporter mRNA levels in DLPFC and found female depressed suicides had greater expression levels of Excitatory Amino Acid Transporter 2, Vesicular Glutamate Transporter 1 and 2 compared to non-psychiatric controls, but not when compared with depressed non-suicide decedents, where there were no sex differences [87]. These findings may reflect compensatory changes in glutamate transporter expression in response to altered excitatory neurotransmission, shifts in DLPFC excitatory tone or involvement of glial cells, particularly astrocytes, which regulate glutamate uptake and synaptic homeostasis [88]. The second study examined gene expression of the metabotropic glutamate receptor (mGluR5) and its scaffolding proteins, Homer1a and p11, in three brain regions: DLPFC, amygdala and hippocampus. Suicides had greater mGluR5 expression in amygdala and hippocampus but not DLPFC and showed differences in p11 and Homer1a expression across brain regions relative to controls [62]. Of note, several smaller studies on the glutamatergic system in suicide are described in the Supplemental Tables. In conclusion, there is not enough evidence to substantiate associations of glutamatergic alterations with suicide, with a need for more large-scale data.

Little is known about the major inhibitory neurotransmitter gamma-aminobutyric acid’s (GABA) role in suicide [4]. Two studies assessed expression of glutamic acid decarboxylase (GAD), an enzyme involved in GABA synthesis, in suicide decedents’ brains. The first found elevated levels of the full-length transcript of GAD2 in DLPFC of suicide decedents with schizophrenia compared to non-suicide decedents [89]. The second study reported increased GAD1 mRNA full transcript in DLPFC, but not hippocampus, of suicide decedents with schizophrenia compared to non-suicides, with no differences observed in mRNA levels of other GAD1 transcripts [90]. A single genetic study compared polymorphisms from functional regions of 24 glutamatergic and GABAergic genes (4 transporters, 4 enzymes, and 16 receptors) finding no association between the 119 single nucleotide polymorphisms tested and suicide [91]. Thus, knowledge on GABAergic alterations in suicide is still lacking.

### Opioid system

The opioid system is hypothesized to be biologically linked to suicide risk because physical [92] and social pain [93], regulated by opioid signaling, impact reward circuitry and may increase vulnerability to suicide. Three studies examined the opioid system in suicide. One evaluated nociceptin/orphanin fluoroquinolone (N/OFQ) peptide, a modulator that activates opioid-like peptide receptors and decreases neuronal excitability. It found decreased N/OFQ gene expression in dorsal ACC of suicide decedents compared to controls and noted no differences in anterior insula or mediodorsal thalamus [94]. Another study found epigenetic changes and expression differences in the insula kappa receptor gene to be associated with child abuse, but not suicide [95]. In a study of bipolar disorder comparing PFC (BA46) methylation profiles between suicide decedents and non-suicides, 330 differentially methylated regions were identified, with genes involved in the opioid signaling pathway being overrepresented among these regions [96].

Therefore, despite emerging clinical and epidemiological data linking the opioid system with suicide, only a few studies have examined opioid neurobiological pathways in suicide, highlighting the need for further research.

### Cannabinoid system

The cannabinoid system is garnering interest for its potential involvement in suicidal behavior [97] given its interactions with processes involved in suicide risk, including stress pathways [98] and neuroplasticity [99]. Two studies of the cannabinoid system in suicide yielded inconsistent findings. One which included individuals with alcohol use disorder found increased expression of the CB1 receptor protein, but not mRNA, in suicide decedents’ DLPFC compared to non-suicide decedents, indicating involvement of post-transcriptional modifications in suicide decedents [100]. A second study found increased expression of CB1 receptor mRNA in DLPFC of suicide decedents with schizophrenia compared to non-suicides [101]. This nascent area of work requires further research to determine whether cannabinoid signaling is altered in suicide decedents’ brains.

## Other findings

Six manuscripts could not be categorized as above. One reported no differences in orbital PFC connexin43 (a gap junction protein in astrocytes) between suicides and non-suicides [102], and another reported no differences in tau or amyloid-beta protein levels in suicides versus controls [103]. Lower levels of acetylserotonin-O-methyltransferase protein, which synthesizes melatonin in pineal glands were reported among suicide decedents compared to controls [104]; greater gene expression, but similar protein levels, of phospholipase B in PFC of suicides versus non-suicides were reported [105]; as were shorter telomeres and altered mitochondrial DNA content in blood and PFC of suicides compared to controls [106]. Finally, a small GWAS (313 suicides and 294 controls) reported four significant SNPs associated with suicide death, likewise without a clear connection to a specific biological pathway [107].

## Discussion

This review summarizes the growing effort to unravel the biological processes associated with suicide. It builds on and substantiates findings from our 2014 review, further underscoring the role of stress biology in suicide, either directly or through downstream pathways such as inflammation, neuroplasticity, and neurotransmitter systems, primarily serotonin. Our carefully selected inclusion criteria allowed a focus on studies that were at least moderately powered and helped minimize the likelihood that neurobiological differences identified were attributable to comorbid psychiatric disorders rather than to suicide. Moreover, the strengths of the studies included in this review—the larger sizes, the strict inclusion criteria for diagnostic group comparison, and the fact that many studies applied novel methods not previously available— support the functional relevance of the implicated systems to suicide. These studies reported converging evidence documenting alterations in the expression and regulation of components of the HPA axis, from peripheral cortisol levels to central expression of GRs and their regulatory proteins, as well as evidence for gene expression and epigenetic changes that relate to suicide among individuals with childhood adversity. They also suggest the immune system plays a role in suicide, as evidenced by the association between peripheral inflammation and suicide. However, no specific cytokine or pathogen has been identified as a specific marker for suicide, and currently, no strong evidence exists of the involvement of brain inflammation in suicide’s pathophysiology. Among studies of neurotransmitter systems, most highlighted serotonergic alterations in suicide, primarily in PFC, using different methods. Nevertheless, despite the substantial number of quality manuscripts published in the past 10 years, a lack of replication with well powered studies means that a cohesive biological signature underpinning suicide remains elusive, perhaps due to the considerable heterogeneity and inconsistency across studies.

A notable methodological trend is the avaliability of data-driven “omics” analyses of the genome [108–112], transcriptome [52, 113] and methylome [37, 96, 114] of suicide decedents, as opposed to molecular target studies that test the role of a specific gene or protein. Importantly, three genetic studies did not meet inclusion criteria due to their use of live controls. Notably, the largest genome-wide association study (GWAS) to date—comprising over 3400 suicides and 15,000 controls [111] identified six genome-wide significant variants at two loci on chromosomes 13 and 15; however, these variants were not directly linked to known biological pathways. Additionally, a study of rare genetic variants in 2672 suicides and over 50,000 controls identified five novel, high-impact rare variants in genes related to immune regulation and homeostasis, highlighting the role of rare variants and these pathways in suicide risk [115]. Together, these findings underscore the importance of both common and rare genetic variation in suicide risk. Notably, a GWAS of copy number variants (CNVs) found no associations, either for specific CNVs or for overall rare CNV burden, between 276 suicides and 1133 controls, possibly due to limited power [116]. Such data-driven analyses will likely become increasingly common in postmortem suicide research as availability of genomic, RNA-seq, ATAC-seq, proteomic, and methylomic technologies and datasets grows, and with the development of novel computational methods to analyze such databases. This trend aligns with the evolution seen in psychiatric genetics research from “candidate gene” to genome-wide approaches [117] leveraging data from large and diverse populations and employing functional genomics for biological mapping [110]. However, the transition to brain multi-omics (including single cell genomics) in postmortem suicide research is limited by the need for collaborative studies with large samples, and powerful bioinformatic efforts for data analytics [118]. Specifically, proteomic research—a rapidly expanding field—remains notably underrepresented in postmortem suicide studies, but its continued growth promises to facilitate more investigations soon. Moreover, to unveil the potential of biomarkers as treatment targets, marker validation studies must be followed by mechanistic testing in cellular (e.g., iPSC-derived neurons, organoids) and animal models. Such research is critical to understanding cellular and behavioral effects of identified molecular targets.

This review’s limitations are primarily related to the inclusion and exclusion criteria, which may have influenced the findings. First, requiring at least 20 subjects in each comparison group might have excluded some relevant studies. However, we summarize these findings in 4 Supplemental Tables for further examination. Second, although the inclusion and exclusion criteria aimed to compare suicide and non-suicide decedents within diagnostic groups to isolate the effects of suicide from psychopathology, the inclusion of three exceptions introduced some heterogeneity into the literature synthesis. Studies comparing suicide decedents to “heterogeneous” or “undefined psychiatric status” decedents could introduce bias. Third, we did not account for psychiatric disorder severity, as this aspect is not addressed in current postmortem research. Notably, while psychiatric disorder severity may be germane to suicide risk, severity does not always correlate with suicide risk, given suicidal behavior often emerges soon after psychiatric symptom onset, rather than progressively increasing as disease worsens. Fourth, by presenting results according to individual biological systems, we cannot do justice to the inherently interconnected nature of the pathways covered. Fifth, the reviewed postmortem studies cannot determine whether biosignatures reflect long-term risk (trait), with the exception of genetics, or temporary changes at the time of death (state), which would be instructive. Lastly, we identified only a few studies integrating multiple levels of analysis—such as gene expression, protein expression, and epigenetic regulation—across the biological systems involved in suicide. Most studies focus on a single level, limiting our understanding of how these processes interact. More multi-level research is needed to clarify the complex biology underlying suicide. Despite these challenges, our approach contributes to refining the understanding of suicide’s distinct biology.

## Future directions

This review has identified some key gaps in postmortem suicide research and suggests future approaches (Table 5). First, in terms of study design, there is a need for studies that maximize confidence that findings are suicide specific. This might be addressed by comparisons of suicide and non-suicide decedents within diagnostic groups and studying suicide transdiagnostically, although empirical evidence validating these approaches as able to isolate biomarkers that are suicide specific is lacking. Comparisons of suicide decedents with a specific diagnosis to healthy controls who did not die by suicide fails to distinguish the effects of suicide from those of psychopathology. Second, to ensure adequate power, we urge the scientific community to increase collaborative efforts across brain banks and use harmonized measurements to allow for substantial sample sizes, as has been done in genetic [119] and neuroimaging [120] research. If studies are adequately powered, even null findings will be relevant. Other significant barriers to increasing sample sizes in suicide research include policy barriers to brain donation for suicide research such as complex requirements for informed consent as well as stigma. Strategies to engage next of kin and stigma reduction campaigns may be useful. Moreover, postmortem brain studies currently have limitations beyond sample size, including lack of access to in vivo data in high risk individuals who go on to die by suicide, technical challenges resulting from the method used (gunshot wound to the brain, for example) that exclude a large proportion of suicide deaths given greater than 50% of suicides are due to firearms, impact of postmortem interval, disambiguation of agonal effects versus suicide-related markers, to name a few. Some of these are poised to be overcome. For example, if consortia conducting resting state fMRI studies in high risk individuals could conduct follow up studies to identify participants that later die by suicide, biomarkers for risk of suicide death among suicide attempters might emerge.

**Table 5:** Considerations for conducting future postmortem suicide analyses.

Study phase	Consideration	Hypothesis-driven analyses	Data-driven analyses
Broad planning	Conceptual model	Prior to analysis, consider how the examined gene/protein fits with a conceptual model of suicide (e.g., the stress-diathesis model).	Choose the data type and brain region(s) that you expect to show differences between suicide and comparators based on a conceptual model.
	Identifying the cohort	Aim to increase sample size by combining samples from various brain banks.	
Designing the study	Defining control groups	Prioritize comparisons between groups that are as similar as possible except for the cause of death. For example, compare suicide to non-suicide decedents within diagnostic groups. Avoid comparisons that do not allow inferences about whether results are due to effects of psychiatric disorder or suicide.	
	Choosing the brain structure	Most existing research has focused on the PFC. Aim to replicate/test previously reported findings in the PFC. Consider examining brain structures that were understudied in previous research. Consider brain structures that fit with the conceptual model. For example, if relying on the stress-diathesis model, consider components of limbic system.	
	Power considerations	Calculate power to test differences in the studied gene/protein.	Consider how the number of comparisons and/or measures and/or networks tested could affect robustness of findings.
Leveraging novel methods	Cell type specificity	Test research questions in specific cell types. Consider pre-specifying hypotheses regarding expected differences based on the system examined (e.g., gene/proteins involved in inflammation are more likely to show a signal in brain immune cells).	
	System biology	Address research questions across levels of analyses in a single study by combining analyses of gene expression, regulation, and protein expression and function.	
	Account for the exposome	Given the robust evidence for the association of stress exposures with suicide, aim to include measures of environment in your research (e.g., early life adversity, exposome scores).	
Robustness tests	Maximize impact of findings	Clearly describe whether and how the results of your study align with previous literature.	Aim to generalize findings in at least one more cohort/dataset.
Discussion of findings	Interpretation of the results	Describe how the hypothesis-driven analyses support or weaken the conceptual model.	Describe how findings that were identified without a priori hypotheses, often using multiomic approaches, fit with existing conceptual models.

Third, although this review concentrated on suicide death rather than suicide attempts, approximately half of those dying by suicide made past attempts. Considering suicidal behavior as a continuum, suicide may represent a more severe phenotype compared to suicide attempts. Consequently, future research could leverage pre-mortem findings to identify biomarkers associated with suicide. Fourth, we call on researchers to harness the potential of multiomic analyses across brain regions and apply the most powerful methods that will allow for a deeper understanding of brain biology [118]. To date, most postmortem suicide research has been conducted in PFC tissue, with limited specificity to cell types and circuitry. Neuroimaging studies suggest multiregional alterations may underpin suicidal thoughts and behavior [121], highlighting a need for postmortem research in other brain regions. Moreover, given suicide is driven by complex biological and environmental pathways, it would be prudent to apply technologies that allow integration of genomic and epigenomic data, including Assay for Transposase-Accessible Chromatin using sequencing (ATAC-seq) and methylation assays, across different cell types and brain circuits and in the periphery, using multiomic and systems biology methods and integrating results at the levels of genomic loci, mediating genes and pathways [122]. Finally, we encourage researchers to revisit existing datasets containing postmortem transcriptomic data from psychiatric disorders, even if suicide was not the original variable of interest, as long as relevant associations can be established within the dataset. For example, the NIH NeuroBioBank provides access to high-quality, well-characterized postmortem human brain samples and accompanying genomic and transcriptomic data that can be reanalyzed for suicide-related associations if phenotypic linkage is possible. This relatively modest effort could greatly advance the field by substantially increasing samples, expanding coverage of brain regions, and diversifying the available transcriptomic data.

Additionally, given the association of environmental stress with suicide risk, we call for greater rigor of stress exposure quantification in postmortem suicide work. Gathering an early life adversity history, as in some studies in this review [36, 37, 59, 95], is an excellent starting point. However, a growing understanding of the colinear nature of adversities, requires quantification of poly-environmental stress exposures to capture stress exposure better than is possible by measuring specific experiences in isolation [123]. This aligns with the exposome framework, capturing the totality of non-genetic exposures and linking environmental effects to downstream biological processes representing the biological encoding of stress (e.g., allostatic load [124]). These exposomic models can complement and enhance the explanatory power of models including genetic and other biological data [125]. In fact, multi-level environmental exposures have been leveraged to calculate specific, generalizable exposomic suicide attempt risk scores using risk and protective exposures identified in an exposome-wide association study [126]. However, collecting exposome data postmortem is limited by recall bias and missing records. Alternatives include linking to medical or social records, structured informant interviews, and using geocoded measures, which provide objective data without relying on recall. Applying exposomic approaches in postmortem suicide research can improve alignment of environmental stress exposure with biological measurements.

## Conclusions

Literature examining biological correlates of suicide suggests dysregulation in pathways involved in stress biology, inflammation, and serotonergic neurotransmission. Despite advances, current evidence does not provide sufficiently robust or specific biomarkers to support translation into effective interventions or risk assessment strategies. There is a paucity of reproducible research across brain regions and brain banks; few studies have leveraged novel multiomic analyses across biological systems and specific cell types. These approaches are critical for capturing the complex biology underpinning suicide pathogenesis. Future research should emphasize reproducibility, include larger samples, and employ study designs that enhance specificity of biological findings to suicide.

## Supplementary information

Supplemental Table of Contents and Table Legends and eMethods

Supplemental Table 1

Supplemental Table 2

Supplemental Table 3

Supplemental Table 4

## References

[1] WHO: Suicide. World Health Organization: https://www.who.int/news-room/fact-sheets/detail/suicide

[2] Garnett MF, Curtin SC: Suicide mortality in the United States, 2002-2022. NCHS Data Brief 2024 10.15620/cdc/160504 PMC11615958 39392858

[3] Mann JJ, Waternaux C, Haas GL, Malone KM. Toward a clinical model of suicidal behavior in psychiatric patients. American Journal of Psychiatry. 1999;156:181–9. 9989552 10.1176/ajp.156.2.181

[4] Oquendo MA, Sullivan GM, Sudol K, Baca-Garcia E, Stanley BH, Sublette EM, et al. Toward a biosignature for suicide. American Journal of Psychiatry. 2014;171:1259–77. 10.1176/appi.ajp.2014.14020194 25263730 10.1176/appi.ajp.2014.14020194 PMC4356635

[5] Oquendo MA, Galfalvy H, Sullivan GM, Miller JM, Milak MM, Sublette EM, et al. Positron emission tomographic imaging of the serotonergic system and prediction of risk and lethality of future suicidal behavior. JAMA Psychiatry. 2016;73:1048–55. https://jamanetwork.com/journals/jamapsychiatry/fullarticle/2537047 27463606 10.1001/jamapsychiatry.2016.1478 PMC6552665

[6] Herzog S, Galfalvy H, Keilp JG, Mann JJ, Sublette EM, Burke A, et al. Relationship of stress-reactive cortisol to suicidal intent of prior attempts in major depression. Psychiatry Res. 2023;327:115315 https://www.sciencedirect.com/science/article/abs/pii/S0165178123002652?via%3Dihub 37542793 10.1016/j.psychres.2023.115315 PMC10530442

[7] Berardelli I, Serafini G, Cortese N, Fiasche F, O’Connor R, Pompili M. The involvement of hypothalamus–pituitary–adrenal (HPA) axis in suicide risk. Brain Sci. 2020;10:653 https://www.mdpi.com/2076-3425/10/9/653/htm 32967089 10.3390/brainsci10090653 PMC7565104

[8] Appelbaum LG, Shenasa MA, Stolz L, Daskalakis Z. Synaptic plasticity and mental health: methods, challenges and opportunities. Neuropsychopharmacology. 2022;48:113–20. https://www.nature.com/articles/s41386-022-01370-w 35810199 10.1038/s41386-022-01370-w PMC9700665

[9] Baldini V, Gnazzo M, Varallo G, Atti AR, De Ronchi D, Fiorillo A, et al. Inflammatory markers and suicidal behavior: A comprehensive review of emerging evidence. Ann Gen Psychiatry. 2025;24:1–10. 10.1186/s12991-025-00575-9 40442662 10.1186/s12991-025-00575-9 PMC12124015

[10] Wisłowska-Stanek A, Kołosowska K, Maciejak P. Neurobiological basis of increased risk for suicidal behaviour. Cells. 2021;10:2519 https://www.mdpi.com/2073-4409/10/10/2519/htm 34685499 10.3390/cells10102519 PMC8534256

[11] Underwood MD, Kassir SA, Bakalian MJ, Galfalvi H, Dwork AJ, Mann JJ, et al. Serotonin receptors and suicide, major depression, alcohol use disorder and reported early life adversity. Transl Psychiatry. 2018;8:1–15. https://www.nature.com/articles/s41398-018-0309-1 30552318 10.1038/s41398-018-0309-1 PMC6294796

[12] Turecki G, Brent DA. Suicide and suicidal behaviour. The Lancet. 2016;387:1227–39. http://www.ncbi.nlm.nih.gov/pubmed/26385066 10.1016/S0140-6736(15)00234-2 PMC5319859 26385066

[13] Fazel S, Runeson B. Suicide. New England Journal of Medicine. 2020;382:266–74. 10.1056/NEJMra1902944 31940700 10.1056/NEJMra1902944 PMC7116087

[14] John Mann J, Rizk MM. A brain-centric model of suicidal behavior. American Journal of Psychiatry. 2020;177:902–16. 10.1176/appi.ajp.2020.20081224 32998550 10.1176/appi.ajp.2020.20081224 PMC7676389

[15] Tidemalm D, Runeson B, Waern M, Frisell T, Carlström E, Lichtenstein P, et al. Familial clustering of suicide risk: a total population study of 11.4 million individuals. Psychol Med. 2011;41:2527–34. https://www.cambridge.org/core/journals/psychological-medicine/article/familial-clustering-of-suicide-risk-a-total-population-study-of-114-million-individuals/85DB76A97AE7391805E251A39F78BC20 21733212 10.1017/S0033291711000833 PMC3207221

[16] Björkenstam C, Kosidou K, Björkenstam E. Childhood adversity and risk of suicide: cohort study of 548 721 adolescents and young adults in Sweden. BMJ. 2017;357:j1334 https://www.bmj.com/content/357/bmj.j1334 28424157 10.1136/bmj.j1334

[17] Dempsey CL, Benedek DM, Zuromski KL, Nock MK, Brent DA, Ao J, Georg MW, et al. Recent stressful experiences and suicide risk: implications for suicide prevention and intervention in U.S. Army Soldiers. Psychiatric Research and Clinical Practice. 2023;5:24–36. 10.1176/appi.prcp.20220027 36909141 10.1176/appi.prcp.20220027 PMC9997076

[18] Calcia MA, Bonsall DR, Bloomfield PS, Selvaraj S, Barichello T, Howes OD. Stress and neuroinflammation: A systematic review of the effects of stress on microglia and the implications for mental illness. Psychopharmacology (Berl). 2016;233:1637–1650. 10.1007/s00213-016-4218-9 26847047 10.1007/s00213-016-4218-9 PMC4828495

[19] Ménard C, Pfau ML, Hodes GE, Russo SJ. Immune and neuroendocrine mechanisms of stress vulnerability and resilience. Neuropsychopharmacology. 2017;42:62–80. https://www.nature.com/articles/npp201690 27291462 10.1038/npp.2016.90 PMC5143517

[20] Paolicelli RC, Bolasco G, Pagani F, Maggi L, Scianni M, Panzanelli P, et al. Synaptic pruning by microglia is necessary for normal brain development. Science. 2011;333:1456–8. 10.1126/science.1202529 21778362 10.1126/science.1202529

[21] Beurel E, Toups M, Nemeroff CB. The bidirectional relationship of depression and inflammation: double trouble. Neuron. 2020;107:234–56. 32553197 10.1016/j.neuron.2020.06.002 PMC7381373

[22] Herman JP, McKlveen JM, Ghosal S, Kopp B, Wulsin A, Makinson R, et al. Regulation of the hypothalamic-pituitary-adrenocortical stress response. Compr Physiol. 2016;6:603–21. 10.1002/j.2040-4603.2016.tb00694.x 27065163 10.1002/cphy.c150015 PMC4867107

[23] de Kloet ER, Joëls M, Holsboer F. Stress and the brain: from adaptation to disease. Nat Rev Neurosci. 2005;6:463–75. 15891777 10.1038/nrn1683

[24] Binder EB. The role of FKBP5, a co-chaperone of the glucocorticoid receptor in the pathogenesis and therapy of affective and anxiety disorders. Psychoneuroendocrinology. 2009;34:S186–S195. 19560279 10.1016/j.psyneuen.2009.05.021

[25] Rice L, Waters CE, Eccles J, Garside H, Sommer P, Kay P, et al. Identification and functional analysis of SKA2 interaction with the glucocorticoid receptor. Journal of Endocrinology. 2008;198:499–509. https://joe.bioscientifica.com/view/journals/joe/198/3/499.xml 18583474 10.1677/JOE-08-0019 PMC2518725

[26] Myers B, McKlveen JM, Herman JP. Glucocorticoid actions on synapses, circuits, and behavior: Implications for the energetics of stress. Front Neuroendocrinol. 2014;35:180–96. https://www.sciencedirect.com/science/article/abs/pii/S0091302213000733?via%3Dihub 24361584 10.1016/j.yfrne.2013.12.003 PMC4422101

[27] Laugesen K, Farkas DK, Vestergaard M, Jørgensen JOL, Petersen I, Sørensen HT. Glucocorticoid use and risk of suicide: a danish population-based case-control study. World Psychiatry. 2021;20:142–3. 10.1002/wps.20831 33432747 10.1002/wps.20831 PMC7801823

[28] Ragnarsson O, Olsson DS, Papakokkinou E, Chantzichristos D, Dahlqvist P, Segerstedt E, et al. Overall and disease-specific mortality in patients with cushing disease: a swedish nationwide study. J Clin Endocrinol Metab. 2019;104:2375–84. 10.1210/jc.2018-02524 30715394 10.1210/jc.2018-02524

[29] Chaurasia S, Ganvir R, Pandey RK, Singh S, Yadav J, Malik R, et al. Gross changes in adrenal glands in suicidal and sudden death cases: a postmortem study. Cureus. 2023;15:e51175 https://www.cureus.com/articles/212917-gross-changes-in-adrenal-glands-in-suicidal-and-sudden-death-cases-a-postmortem-study 38283486 10.7759/cureus.51175 PMC10811436

[30] Pandey GN, Rizavi HS, Bhaumik R, Ren X. Increased protein and mRNA expression of corticotropin-releasing factor (CRH), decreased CRH receptors and CRH binding protein in specific postmortem brain areas of teenage suicide subjects. Psychoneuroendocrinology. 2019;106:233–43. 31005044 10.1016/j.psyneuen.2019.04.015 PMC7061258

[31] Rizavi HS, Khan OS, Zhang H, Bhaumik R, Grayson DR, Pandey GN. Methylation and expression of glucocorticoid receptor exon-1 variants and FKBP5 in teenage suicide-completers. Translational. Psychiatry. 2023;13:1–9. https://www.nature.com/articles/s41398-023-02345-1 10.1038/s41398-023-02345-1 PMC9925759 36781843

[32] Pandey GN, Rizavi HS, Zhang H, Bhaumik R, Ren X. The expression of the suicide-associated gene SKA2 is decreased in the prefrontal cortex of suicide victims but not of nonsuicidal patients. Int J Neuropsychopharmacol. 2016;19:1–10. 10.1093/ijnp/pyw015 10.1093/ijnp/pyw015 PMC5006192 26902949

[33] Kouter K, Zupanc T, Videtič Paska A. Targeted sequencing approach: comprehensive analysis of DNA methylation and gene expression across blood and brain regions in suicide victims. World J Biol Psychiatry. 2023;24:12–23. 10.1080/15622975.2022.2046291 35200087 10.1080/15622975.2022.2046291

[34] Fudalej S, Kopera M, Wolynczyk-Gmaj D, Fudalej M, Krajewski P, Wasilewska K, et al. Association between FKBP5 functional polymorphisms and completed suicide. Neuropsychobiology. 2015;72:126–31. 10.1159/000441659 26630184 10.1159/000441659

[35] Park S, Hong JP, Lee JK, Park YM, Park Y, Jeon J, et al. Associations between the neuron-specific glucocorticoid receptor (NR3C1) Bcl-1 polymorphisms and suicide in cancer patients within the first year of diagnosis. Behavioral and Brain Functions. 2016;12:1–5. 10.1186/s12993-016-0104-1 27401254 10.1186/s12993-016-0104-1 PMC4940702

[36] Underwood MD, Galfalvy H, Hsiung SC, Liu Y, Simpson NR, Bakalian MJ, et al. A stress protein–based suicide prediction score and relationship to reported early-life adversity and recent life stress. Int J Neuropsychopharmacol. 2023;26:501–12. 10.1093/ijnp/pyad025 37243534 10.1093/ijnp/pyad025 PMC10388383

[37] Lutz PE, Tanti A, Gasecka A, Barnett-Burns S, Kim JJ, Zhou Y, et al. Association of a history of child abuse with impaired myelination in the anterior cingulate cortex: convergent epigenetic, transcriptional, and morphological evidence. American Journal of Psychiatry. 2017;174:1185–94. 10.1176/appi.ajp.2017.16111286 28750583 10.1176/appi.ajp.2017.16111286

[38] Black C, Miller BJ. Meta-analysis of cytokines and chemokines in suicidality: distinguishing suicidal versus nonsuicidal patients. Biol Psychiatry. 2015;78:28–37. 25541493 10.1016/j.biopsych.2014.10.014

[39] de la Bengoechea-Fortes SP, Ramírez-Expósito MJ, Martínez-Martos JM. Suicide, neuroinflammation and other physiological alterations. Eur Arch Psychiatry Clin Neurosci. 2024;274:1037–49. 10.1007/s00406-023-01584-z 36913003 10.1007/s00406-023-01584-z PMC10009854

[40] Kaushik R, Nayak B, Patra BN, Sharma N, Mohapatra BK, Singh H, et al. Association of TNF-α cytokine gene polymorphism with suicide in Indian population. Asian J Psychiatr. 2023;79:103330. 36413902 10.1016/j.ajp.2022.103330

[41] Wang Q, Roy B, Turecki G, Shelton RC, Dwivedi Y. Role of complex epigenetic switching in tumor necrosis factor-α upregulation in the prefrontal cortex of suicide subjects. American Journal of Psychiatry. 2018;175:262–74. 10.1176/appi.ajp.2017.16070759 29361849 10.1176/appi.ajp.2017.16070759 PMC5832541

[42] Clark SM, Pocivavsek A, Nicholson JD, Notarangelo FM, Langenberg P, McMahon RP, et al. Reduced kynurenine pathway metabolism and cytokine expression in the prefrontal cortex of depressed individuals. J Psychiatry Neurosci. 2016;41:386–94. https://www.jpn.ca/content/41/6/386 27070351 10.1503/jpn.150226 PMC5082509

[43] Shimmyo N, Hishimoto A, Otsuka I, Okazaki S, Boku S, Mouri K, et al. Association study of <em>MIF < /em> promoter polymorphisms with suicide completers in the Japanese population. Neuropsychiatr Dis Treat. 2017;13:899–908. https://www.dovepress.com/association-study-of-mif-promoter-polymorphisms-with-suicide-completer-peer-reviewed-fulltext-article-NDT 28367056 10.2147/NDT.S130855 PMC5370383

[44] Zheng H, Webster MJ, Weickert CS, Beasley CL, Paulus MP, Yolken RH, et al. Cytomegalovirus antibodies are associated with mood disorders, suicide, markers of neuroinflammation, and microglia activation in postmortem brain samples. Mol Psychiatry. 2023;28:5282–92. https://www.nature.com/articles/s41380-023-02162-4 37391529 10.1038/s41380-023-02162-4 PMC10756933

[45] Dickerson F, Origoni A, Schweinfurth LAB, Stallings C, Savage CLG, Sweeney K, et al. Clinical and serological predictors of suicide in schizophrenia and major mood disorders. Journal of Nervous and Mental Disease. 2018;206:173–8. https://journals.lww.com/jonmd/fulltext/2018/03000/clinical_and_serological_predictors_of_suicide_in.4.aspx 29474231 10.1097/NMD.0000000000000772

[46] Russell AE, Mars B, Wen CP, Chang SS, Gunnell D. Evidence for an association between inflammatory markers and suicide: a cohort study based on 359,849 to 462,747 Taiwanese adults. J Affect Disord. 2021;281:967–71. 33250203 10.1016/j.jad.2020.10.047

[47] Batty GD, Jung KJ, Lee S, Back JH, Jee SH. Systemic inflammation and suicide risk: cohort study of 419 527 Korean men and women. J Epidemiol Community Health. 2018;72:572–4. https://jech.bmj.com/content/72/7/572 29572361 10.1136/jech-2017-210086 PMC6031272

[48] Batty GD, Bell S, Stamatakis E, Kivimäki M. Association of systemic inflammation with risk of completed suicide in the general population. JAMA Psychiatry. 2016;73:993–5. https://jamanetwork.com/journals/jamapsychiatry/fullarticle/2542679 27532220 10.1001/jamapsychiatry.2016.1805

[49] Bultink IEM, De Vries F, Van Vollenhoven RF, Lalmohamed A. Mortality, causes of death and influence of medication use in patients with systemic lupus erythematosus vs matched controls. Rheumatology. 2021;60:207–16. 10.1093/rheumatology/keaa267 32653901 10.1093/rheumatology/keaa267 PMC8312724

[50] Punzi G, Ursini G, Viscanti G, Radulescu E, Shin JH, Quarto T, et al. Association of a noncoding RNA postmortem with suicide by violent means and in vivo with aggressive phenotypes. Biol Psychiatry. 2019;85:417–24. 30600091 10.1016/j.biopsych.2018.11.002

[51] Cabrera-Mendoza B, Fresno C, Monroy-Jaramillo N, Walss-Bass C, Glahn DC, Ostrosky-Wegman P, et al. Sex differences in brain gene expression among suicide completers. J Affect Disord. 2020;267:67–77. 32063575 10.1016/j.jad.2020.01.167

[52] Punzi G, Ursini G, Chen Q, Radulescu E, Tao R, Huuki LA, et al. Genetics and brain transcriptomics of completed suicide. American Journal of Psychiatry. 2022;179:226–41. 10.1176/appi.ajp.2021.21030299 35236118 10.1176/appi.ajp.2021.21030299 PMC8908792

[53] Huang Z, Xie N, Illes P, Di Virgilio F, Ulrich H, Semyanov A, et al. From purines to purinergic signalling: molecular functions and human diseases. Signal Transduct Target Ther. 2021;6:1–20. https://www.nature.com/articles/s41392-021-00553-z 33907179 10.1038/s41392-021-00553-z PMC8079716

[54] Hamazaki K, Maekawa M, Toyota T, Dean B, Hamazaki T, Yoshikawa T. Fatty acid composition of the postmortem prefrontal cortex of patients with schizophrenia, bipolar disorder, and major depressive disorder. Psychiatry Res. 2015;227:353–9. 25858798 10.1016/j.psychres.2015.01.004

[55] Hamazaki K, Maekawa M, Toyota T, Iwayama Y, Dean B, Hamazaki T, et al. Fatty acid composition and fatty acid binding protein expression in the postmortem frontal cortex of patients with schizophrenia: A case–control study. Schizophr Res. 2016;171:225–32. 26792082 10.1016/j.schres.2016.01.014

[56] Hamazaki K, Maekawa M, Toyota T, Dean B, Hamazaki T, Yoshikawa T, et al. Fatty acid composition of the postmortem corpus callosum of patients with schizophrenia, bipolar disorder, or major depressive disorder. Eur Psychiatry. 2017;39:51–56. 27821355 10.1016/j.eurpsy.2016.05.007

[57] Wang CS, Kavalali ET, Monteggia LM. BDNF signaling in context: From synaptic regulation to psychiatric disorders. Cell. 2022;185:62–76. 34963057 10.1016/j.cell.2021.12.003 PMC8741740

[58] Notaras M, van den Buuse M. Neurobiology of BDNF in fear memory, sensitivity to stress, and stress-related disorders. Mol Psychiatry. 2020;25:2251–74. 31900428 10.1038/s41380-019-0639-2

[59] Youssef MM, Underwood MD, Huang YY, Hsiung SC, Liu Y, Simpson NR, et al. Association of BDNF Val66Met polymorphism and brain BDNF levels with major depression and suicide. Int J Neuropsychopharmacol. 2018;21:528–38. 10.1093/ijnp/pyy008 29432620 10.1093/ijnp/pyy008 PMC6007393

[60] Liu X, Li S, Yu Y, Hu J, Xu Y. Changes in Plasma TPH2, GDNF, Trk-b, BDNF, and proBDNF in people who died by suicide. Brain Sci. 2023;13:1096 https://www.mdpi.com/2076-3425/13/7/1096/htm 37509026 10.3390/brainsci13071096 PMC10377529

[61] Ropret S, Kouter K, Zupanc T, Videtic Paska A. BDNF methylation and mRNA expression in brain and blood of completed suicides in Slovenia. World J Psychiatry. 2021;11:1301–13. http://www.ncbi.nlm.nih.gov/pubmed/35070779 35070779 10.5498/wjp.v11.i12.1301 PMC8717036

[62] García-Gutiérrez MS, Navarro D, Torregrosa AB, Viudez-Martínez A, Giner S, Manzanares J. Alterations of BDNF, mGluR5, Homer1a, p11 and excitatory/inhibitory balance in corticolimbic brain regions of suicide decedents. J Affect Disord. 2023;339:366–76. 37437733 10.1016/j.jad.2023.07.003

[63] Zou Y, Zhang Y, Tu M, Ye Y, Li M, Ran R, et al. Brain-derived neurotrophic factor levels across psychiatric disorders: A systemic review and network meta-analysis. Prog Neuropsychopharmacol Biol Psychiatry. 2024;131:110954 https://www.sciencedirect.com/science/article/abs/pii/S0278584624000228?via%3Dihub 38286331 10.1016/j.pnpbp.2024.110954

[64] Maussion G, Yang J, Suderman M, Diallo A, Nagy C, Arnovitz M, et al. Functional DNA methylation in a transcript specific 3′UTR region of TrkB associates with suicide. Epigenetics. 2014;9:1061–70. 10.4161/epi.29068 24802768 10.4161/epi.29068 PMC4164491

[65] Zarrilli F, Amato F, Keller S, Tomaiuolo R, Keller S, Florio E, et al. Tropomyosin-related kinase B receptor polymorphisms and isoforms expression in suicide victims. Psychiatry Res. 2014;220:725–6. 25110312 10.1016/j.psychres.2014.07.036

[66] Barfield ET, Gourley SL. Prefrontal cortical trkB, glucocorticoids, and their interactions in stress and developmental contexts. Neurosci Biobehav Rev. 2018;95:535–58. https://www.sciencedirect.com/science/article/abs/pii/S0149763418304895?via%3Dihub 30477984 10.1016/j.neubiorev.2018.10.015 PMC6392187

[67] Berger M, Gray JA, Roth BL. The expanded biology of serotonin. Annu Rev Med. 2009;60:355–66. 10.1146/annurev.med.60.042307.110802 19630576 10.1146/annurev.med.60.042307.110802 PMC5864293

[68] Carhart-Harris RL, Nutt DJ. Serotonin and brain function: A tale of two receptors. J Psychopharmacol. 2017;31:1091–120. 10.1177/0269881117725915 28858536 10.1177/0269881117725915 PMC5606297

[69] Di Narzo AF, Kozlenkov A, Roussos P, Hao K, Hurd Y, Lewis DA, et al. A unique gene expression signature associated with serotonin 2 C receptor RNA editing in the prefrontal cortex and altered in suicide. Hum Mol Genet. 2014;23:4801–13. 10.1093/hmg/ddu195 24781207 10.1093/hmg/ddu195 PMC4140462

[70] Ramos-Rosales D, Méndez-Hernández E, Salas-Pacheco J, Salas-Leal A, Urtiz-Estrada N, Barraza-Salas M, et al. Differential expression of HTR2A and MAOA genes in the prefrontal cortex and hypothalamus of suicide victims from Mexican population. Neurosci Lett. 2022;778:136611. 35364128 10.1016/j.neulet.2022.136611

[71] Odagaki Y, Kinoshita M, Meana JJ, Callado LF, García-Sevilla JA. 5-HT2A receptor-mediated Gαq/11 activation in psychiatric disorders: A postmortem study. World J Biol Psychiatry. 2021;22:505–15. 10.1080/15622975.2020.1839967 33084439 10.1080/15622975.2020.1839967

[72] Xu J, Zhang GL, Cheng YQ, Chen B, Dong Y, Li L, et al. Hypomethylation of the HTR1A promoter region and high expression of HTR1A in the peripheral blood lymphocytes of patients with systemic lupus erythematosus. Lupus. 2011;20:678–89. 10.1177/0961203310394892?icid=int.sj-full-text.similar-articles.7 21382916 10.1177/0961203310394892

[73] Rahikainen AL, Majaharju S, Haukka J, Palo JU, Sajantila A. Serotonergic 5HTTLPR/rs25531 s-allele homozygosity associates with violent suicides in male citalopram users. American Journal of Medical Genetics Part B: Neuropsychiatric Genetics. 2017;174:691–700. 10.1002/ajmg.b.32553 10.1002/ajmg.b.32553 28608626

[74] Bach H, Arango V, Kassir SA, Dwork AJ, Mann JJ, Underwood MD. Cigarette smoking and tryptophan hydroxylase 2 mRNA in the dorsal raphe nucleus in suicides. Archives of Suicide Research. 2016;20:451–62. 10.1080/13811118.2015.1048398 26954509 10.1080/13811118.2015.1048398 PMC4920715

[75] Krzyżanowska M, Steiner J, Karnecki K, Kaliszan M, Brisch R, Wiergowski M, et al. Decreased ribosomal DNA transcription in dorsal raphe nucleus neurons differentiates between suicidal and non-suicidal death. Eur Arch Psychiatry Clin Neurosci. 2016;266:217–24. 10.1007/s00406-015-0655-4 26590846 10.1007/s00406-015-0655-4 PMC4819736

[76] Krzyzanowska M, Steiner J, Brisch R, Mawrin C, Busse S, Karnecki K, et al. Decreased ribosomal DNA transcription in dorsal raphe nucleus neurons is specific for suicide regardless of psychiatric diagnosis. Psychiatry Res. 2016;241:43–46. 27155286 10.1016/j.psychres.2016.04.079

[77] Dogan KH, Unaldi M, Demirci S. Evaluation of postmortem cerebrospinal fluid S100B protein and serotonin levels: comparison of suicidal versus nonsuicidal deaths in Konya, Turkey. J Forensic Sci. 2016;61:1285–91. 10.1111/1556-4029.13124 27282656 10.1111/1556-4029.13124

[78] Bristow GC, Eisenlohr-Moul T, Lotesto K, Sodhi MS. Sex differences in the transcription of monoamine transporters in major depression. J Affect Disord. 2021;295:1215–9. 34706435 10.1016/j.jad.2021.08.124

[79] Rivero G, Martín-Guerrero I, de Prado E, Gabilondo AM, Callado LF, García-Sevilla JA, et al. Alpha2C-adrenoceptor Del322-325 polymorphism and risk of psychiatric disorders: significant association with opiate abuse and dependence. World J Biol Psychiatry. 2016;17:308–15. 10.3109/15622975.2016.1142608 27007576 10.3109/15622975.2016.1142608

[80] Hasegawa M, Tanifuji T, Okazaki S, Otsuka I, Shirai T, Shindo R, et al. Association of two variable number of tandem repeats in the monoamine oxidase A gene promoter with suicide completion: The present study and meta-analysis. Neuropsychopharmacol Rep. 2023;43:338–34510. 37202909 10.1002/npr2.12344 PMC10496037

[81] Čugura T, Boh J, Zupanc T, Pregelj P, Videtič Paska A. Differences in SNP genotype distributions between complex and simple suicides. Int J Legal Med. 2018;132:1595–601. 10.1007/s00414-018-1820-x 29557505 10.1007/s00414-018-1820-x

[82] Uršič K, Zupanc T, Paska AV. Analysis of promoter polymorphism in monoamine oxidase A (MAOA) gene in completed suicide on Slovenian population. Neurosci Lett. 2018;673:111–5. https://pubmed.ncbi.nlm.nih.gov/29505805/29505805 10.1016/j.neulet.2018.02.063

[83] Lombardo B, Zarrilli F, Ceglia C, Vitale A, Keller S, Sarchiapone M, et al. Two novel genomic rearrangements identified in suicide subjects using a-CGH array. Clin Chem Lab Med. 2015;53:e245–e248. 10.1515/cclm-2014-1255/html 25719327 10.1515/cclm-2014-1255

[84] Border R, Johnson EC, Evans LM, molen A, Berley N, Sullivan PF, et al. No support for historical candidate gene or candidate gene-by-interaction hypotheses for major depression across multiple large samples. American Journal of Psychiatry. 2019;176:376–87. 10.1176/appi.ajp.2018.18070881 30845820 10.1176/appi.ajp.2018.18070881 PMC6548317

[85] Pal MM. Glutamate: the master neurotransmitter and its implications in chronic stress and mood disorders. Front Hum Neurosci. 2021;15:722323 www.frontiersin.org 34776901 10.3389/fnhum.2021.722323 PMC8586693

[86] Wang YT, Wang XL, Feng ST, Chen NH, Wang ZZ, Zhang Y. Novel rapid-acting glutamatergic modulators: targeting the synaptic plasticity in depression. Pharmacol Res. 2021;171:105761. 34242798 10.1016/j.phrs.2021.105761

[87] Powers B, Joyce C, Kleinman JE, Hyde TM, Ajilore O, Leow A, et al. Sex differences in the transcription of glutamate transporters in major depression and suicide. J Affect Disord. 2020;277:244–52. 32836031 10.1016/j.jad.2020.07.055

[88] Danbolt NC. Glutamate uptake. Prog Neurobiol. 2001;65:1–105. https://www.sciencedirect.com/science/article/abs/pii/S0301008200000678?via%3Dihub 11369436 10.1016/s0301-0082(00)00067-8

[89] Davis KN, Tao R, Li C, Gao Y, Gondré-Lewis MC, Lipska BK, et al. GAD2 alternative transcripts in the human prefrontal cortex, and in schizophrenia and affective disorders. PLoS ONE. 2016;11:e0148558 10.1371/journal.pone.0148558 26848839 10.1371/journal.pone.0148558 PMC4744057

[90] Tao R, Davis KN, Li C, Shin JH, Gao Y, Jaffe AE, et al. GAD1 alternative transcripts and DNA methylation in human prefrontal cortex and hippocampus in brain development, schizophrenia. Mol Psychiatry. 2017;23:1496–505. https://www.nature.com/articles/mp2017105 28485403 10.1038/mp.2017.105 PMC7564279

[91] Yin H, Pantazatos SP, Galfalvy H, Huang YY, Rosoklija GB, Dwork AJ, et al. A pilot integrative genomics study of GABA and glutamate neurotransmitter systems in suicide, suicidal behavior, and major depressive disorder. American Journal of Medical Genetics Part B: Neuropsychiatric Genetics. 2016;171:414–26. 10.1002/ajmg.b.32423 10.1002/ajmg.b.32423 PMC4851346 26892569

[92] Elman I, Borsook D, Volkow ND. Pain and suicidality: Insights from reward and addiction neuroscience. Prog Neurobiol. 2013;109:1–27. 23827972 10.1016/j.pneurobio.2013.06.003 PMC4827340

[93] Lutz PE, Courtet P, Calati R. The opioid system and the social brain: implications for depression and suicide. J Neurosci Res. 2020;98:588–600. 10.1002/jnr.24269 30051488 10.1002/jnr.24269

[94] Lutz PE, Zhou Y, Labbe A, Mechawar N, Turecki G. Decreased expression of nociceptin/orphanin FQ in the dorsal anterior cingulate cortex of suicides. Eur Neuropsychopharmacol. 2015;25:2008–14. 26349406 10.1016/j.euroneuro.2015.08.015 PMC4655195

[95] Lutz PE, Gross JA, Dhir SK, Maussion G, Yang J, Bramoulle A, et al. Epigenetic regulation of the kappa opioid receptor by child abuse. Biol Psychiatry. 2018;84:751–61. http://www.biologicalpsychiatryjournal.com/article/S0006322317318127/fulltext 28886759 10.1016/j.biopsych.2017.07.012

[96] Gaine ME, Seifuddin F, Sabunciyan S, Lee RS, Benke KS, Monson ET, et al. Differentially methylated regions in bipolar disorder and suicide. American Journal of Medical Genetics Part B: Neuropsychiatric Genetics. 2019;180:496–507. 10.1002/ajmg.b.32754 10.1002/ajmg.b.32754 PMC8375453 31350827

[97] Mannekote Thippaiah S, Iyengar SS, Vinod KY. Exo- and endo-cannabinoids in depressive and suicidal behaviors. Front Psychiatry. 2021;12:636228. 33967855 10.3389/fpsyt.2021.636228 PMC8102729

[98] Morena M, Patel S, Bains JS, Hill MN. Neurobiological interactions between stress and the endocannabinoid system. Neuropsychopharmacology. 2015;41:80–102. https://www.nature.com/articles/npp2015166 26068727 10.1038/npp.2015.166 PMC4677118

[99] Ferreira FF, Ribeiro FF, Rodrigues RS, Sebastião AM, Xapelli S. Brain-derived neurotrophic factor (BDNF) role in cannabinoid-mediated neurogenesis. Front Cell Neurosci. 2018;12:417033. www.frontiersin.org 10.3389/fncel.2018.00441 PMC6279918 30546297

[100] Erdozain AM, Rubio M, Valdizan EM, Pazos A, Meana JJ, Fernández-Ruiz J, et al. The endocannabinoid system is altered in the post-mortem prefrontal cortex of alcoholic subjects. Addict Biol. 2015;20:773–83. 10.1111/adb.12160 25041461 10.1111/adb.12160

[101] Tao R, Li C, Jaffe AE, Shin JH, Deep-Soboslay A, Yamin R, et al. Cannabinoid receptor CNR1 expression and DNA methylation in human prefrontal cortex, hippocampus and caudate in brain development and schizophrenia. Transl Psychiatry. 2020;10:1–13. https://www.nature.com/articles/s41398-020-0832-8 32433545 10.1038/s41398-020-0832-8 PMC7237456

[102] Miguel-Hidalgo JJ, Wilson BA, Hussain S, Meshram A, Rajkowska G, Stockmeier CA. Reduced connexin 43 immunolabeling in the orbitofrontal cortex in alcohol dependence and depression. J Psychiatr Res. 2014;55:101–9. 24774648 10.1016/j.jpsychires.2014.04.007 PMC4078739

[103] Yoshida K, Hata Y, Ichimata S, Nishida N. Tau and amyloid-β pathology in japanese forensic autopsy series under 40 years of age: prevalence and association with APOE genotype and suicide risk. J Alzheimers Dis. 2019;72:641–52. 10.3233/JAD-190196?url_ver=Z39.88-2003&rfr_id=ori%3Arid%3Acrossref.org&rfr_dat=cr_pub++0pubmed 31594218 10.3233/JAD-190196

[104] Kurtulus Dereli A, Demırci GN, Dodurga Y, Özbal S, Cankurt U, Boz B, et al. Evaluation of human pineal gland acetylserotonin O-methyltransferase immunoreactivity in suicide: A preliminary study. Med Sci Law. 2018;58:233–8. 10.1177/0025802418797178 30185109 10.1177/0025802418797178

[105] Udawela M, Scarr E, Boer S, Um JY, Hannan AJ, McOmish C, et al. Isoform specific differences in phospholipase C beta 1 expression in the prefrontal cortex in schizophrenia and suicide. NPJ Schizophr. 2017;3:1–9. https://www.nature.com/articles/s41537-017-0020-x 28560265 10.1038/s41537-017-0020-x PMC5441535

[106] Otsuka I, Izumi T, Boku S, Kimura A, Zhang Y, Mouri K, et al. Aberrant telomere length and mitochondrial DNA copy number in suicide completers. Sci Rep. 2017;7:1–9. https://www.nature.com/articles/s41598-017-03599-8 28600518 10.1038/s41598-017-03599-8 PMC5466636

[107] Behera C, Kaushik R, Bharti DR, Nayak B, Bhardwaj DN, Pradhan D, et al. PsychArray-based genome wide association study of suicidal deaths in India. Brain Sci. 2023;13:136 https://www.mdpi.com/2076-3425/13/1/136/htm 36672117 10.3390/brainsci13010136 PMC9856809

[108] Galfalvy H, Haghighi F, Hodgkinson C, Goldman D, Oquendo MA, Burke A, et al. A genome-wide association study of suicidal behavior. American Journal of Medical Genetics Part B: Neuropsychiatric Genetics. 2015;168:557–63. 10.1002/ajmg.b.32330 10.1002/ajmg.b.32330 26079190

[109] Otsuka I, Akiyama M, Shirakawa O, Okazaki S, Momozawa Y, Kamatani Y, et al. Genome-wide association studies identify polygenic effects for completed suicide in the Japanese population. Neuropsychopharmacology. 2019;44:2119–24. https://www.nature.com/articles/s41386-019-0506-5 31476763 10.1038/s41386-019-0506-5 PMC6887868

[110] Han S, DiBlasi E, Monson ET, Shabalin A, Ferris E, Chen D, et al. Whole-genome sequencing analysis of suicide deaths integrating brain-regulatory eQTLs data to identify risk loci and genes. Mol Psychiatry. 2023;28:3909–19. https://www.nature.com/articles/s41380-023-02282-x 37794117 10.1038/s41380-023-02282-x PMC10730410

[111] Docherty AR, Shabalin AA, DiBlasi E, Monson E, Mullins N, Adkins DE, et al. Genome-wide association study of suicide death and polygenic prediction of clinical antecedents. American Journal of Psychiatry. 2020;177:917–27. 10.1176/appi.ajp.2020.19101025 32998551 10.1176/appi.ajp.2020.19101025 PMC7872505

[112] Coon H, Darlington TM, DiBlasi E, Callor WB, Ferris E, Fraser A, et al. Genome-wide significant regions in 43 Utah high-risk families implicate multiple genes involved in risk for completed suicide. Mol Psychiatry. 2020;25:3077–90. https://www.nature.com/articles/s41380-018-0282-3 30353169 10.1038/s41380-018-0282-3 PMC6478563

[113] Cabrera B, Monroy-Jaramillo N, Fries GR, Mendoza-Morales RC, García-Dolores F, et al. Brain gene expression pattern of subjects with completed suicide and comorbid substance use disorder. Mol Neuropsychiatry. 2019;5:60–73. 10.1159/000493940 31019919 10.1159/000493940 PMC6465692

[114] Policicchio S, Washer S, Viana J, Iatrou A, Burrage J, Hannon E, et al. Genome-wide DNA methylation meta-analysis in the brains of suicide completers. Translational. Psychiatry. 2020;10:1–13. https://www.nature.com/articles/s41398-020-0752-7 10.1038/s41398-020-0752-7 PMC7031296 32075955

[115] DiBlasi E, Shabalin AA, Monson ET, Keeshin BR, Bakian AV, Kirby AV, et al. Rare protein-coding variants implicate genes involved in risk of suicide death. American Journal of Medical Genetics Part B: Neuropsychiatric Genetics. 2021;186:508–20. 10.1002/ajmg.b.32861 10.1002/ajmg.b.32861 PMC9292859 34042246

[116] Gross JA, Bureau A, Croteau J, Galfalvy H, Oquendo MA, Haghighi F, et al. A Genome-Wide Copy Number Variant Study of Suicidal Behavior. PLoS ONE. 2015;10:e0128369 10.1371/journal.pone.0128369 26010658 10.1371/journal.pone.0128369 PMC4444178

[117] Duncan LE, Ostacher M, Ballon J. How genome-wide association studies (GWAS) made traditional candidate gene studies obsolete. Neuropsychopharmacology. 2019;44:1518–23. https://www.nature.com/articles/s41386-019-0389-5 30982060 10.1038/s41386-019-0389-5 PMC6785091

[118] Boldrini M, Xiao Y, Sing T, Zhu C, Jabbi M, Pantazopoulos H, et al. Omics Approaches to Investigate the Pathogenesis of Suicide. Biol Psychiatry. 2024;96:919–28. 38821194 10.1016/j.biopsych.2024.05.017 PMC11563882

[119] Uffelmann E, Huang QQ, Munung NS, De Vries J, Okada Y, Martin AR, et al. Genome-wide association studies. Nature Reviews Methods Primers. 2021;1:1–21. https://www.nature.com/articles/s43586-021-00056-9

[120] Radua J, Vieta E, Shinohara R, Kochunov P, Quidé Y, Green MJ, et al. Increased power by harmonizing structural MRI site differences with the ComBat batch adjustment method in ENIGMA. Neuroimage. 2020;218:116956. 32470572 10.1016/j.neuroimage.2020.116956 PMC7524039

[121] Schmaal L, van Harmelen AL, Chatzi V, Lippard ETC, Toenders YJ, Averill LA, et al. Imaging suicidal thoughts and behaviors: a comprehensive review of 2 decades of neuroimaging studies. Mol Psychiatry. 2019;25:408–27. https://www.nature.com/articles/s41380-019-0587-x 31787757 10.1038/s41380-019-0587-x PMC6974434

[122] Daskalakis NP, Iatrou A, Chatzinakos C, Jajoo A, Snijders C, Wylie D, et al. Systems biology dissection of PTSD and MDD across brain regions, cell types, and blood. Science. 2024;384:eadh3707 10.1126/science.adh3707 38781393 10.1126/science.adh3707 PMC11203158

[123] Pries LK, Erzin G, Rutten BPF, van Os J, Guloksuz S. Estimating aggregate environmental risk score in psychiatry: the exposome score for schizophrenia. Front Psychiatry. 2021;12:671334 https://pubmed.ncbi.nlm.nih.gov/34122186/34122186 10.3389/fpsyt.2021.671334 PMC8193078

[124] Hoffman KW, Tran KT, Moore TM, Gatavinš MM, Visoki E, Kwon O, et al. Exposomic and polygenic contributions to allostatic load in early adolescence. Nat. Mental Health 2024;2:828–39. 10.1038/s44220-024-00255-9.

[125] Vermeulen R, Schymanski EL, Barabási AL, Miller GW. The exposome and health: Where chemistry meets biology. Science. 2020;367:392–6. http://science.sciencemag.org/31974245 10.1126/science.aay3164 PMC7227413

[126] Visoki E, Moore TM, Zhang X, Tran KT, Ly C, Gatavinš MM, DiDomenico GE, et al. Classification of suicide attempt risk using environmental and lifestyle factors in 3 large youth cohorts. JAMA Psychiatry. 2024;81:1020–9. https://jamanetwork.com/journals/jamapsychiatry/fullarticle/2821270 39018056 10.1001/jamapsychiatry.2024.1887 PMC11255979

