# Supplementary material for: Pursuing the elusive biosignature for suicide: a decennial update
Source: Mol Psychiatry. 2026 Mar 12;31(7):4029–59. doi: 10.1038/s41380-026-03507-5 (PMC13268968; doi:10.1038/s41380-026-03507-5)
Supplement: Supplementary file 1 — Supplemental Table of Contents and Table Legends and eMethods [file 41380_2026_3507_MOESM1_ESM.docx]

**Pursuing the elusive biosignature for suicide: A decennial update and narrative review**

Supplemental Material

Table of contents

**Supplemental eMethods**- Literature search strategy, search criteria, and search terms

**Table S1**- Gene and Protein Expression Findings in Suicide Decedents in Studies with Experimental Groups < 20

**Table S2**- Genetic Findings in Suicide Decedents in Studies with Experimental Groups < 20

**Table S3**- Neurochemical, Autoradiographic, Morphological, and Other Findings in Suicide Decedents in Studies with Experimental Groups < 20

**Table S4**- Findings in Non-Brain Biological Substrate of Suicide Decedents in Studies with Experimental Groups < 20

**Supplemental eMethods**- Literature search strategy and search criteria

We chose search terms based on our approach of studying specific systems as detailed in the review. We applied the following two different syntaxes to derive references from Pubmed, and then combined the list of references obtained from the two:

Syntax 1: (Suicide AND “term” NOT suicidal NOT attempt NOT attempts NOT attempters NOT review) AND (("2014/01/01"[Date - Publication] : "2023/12/31"[Date - Publication]))

Syntax 2: (((“term”) AND suicide) AND postmortem) NOT review) AND ("2014/01/01"[Date - Publication] : "2023/12/31"[Date - Publication])

After retrieval of references, authors read each abstract or manuscript to verify it met inclusion criteria (described in main text). Papers that met inclusion criteria were further searched in their reference list to further identify references that met our inclusion criteria.

We included the individual terms listed below:

| **System** | **Individual search terms** |
| --- | --- |
| **Stress Biology** | HPA axis, Hypothalamus Pituitary Adrenal axis, CRH, Corticotrophin releasing hormone, cortisol, Corticosterone, Glucocorticoid receptor, Mineralocorticoid receptor, FKBP5, Vasopressin, Pro-opiomelanocortin, POMC, ACTH, Urocortin, Adrenal gland. |
| **Neuroinflammation** | Neuroinflammation, Inflammation, microglia, Inflammasome, CD11b, IBA 1,  Cytokines, Cytokine, Interferon, IL-6, Interleukin 6, IL1b/ interleukin 1b, TNF, Tumor necrosis factor, IL-4/ interleukin 4, IL-13/ interleukin 13,  Polyunsaturated Fatty acids (PUFA), docosahexaenoic acid (DHA), eicosapentaenoic acid (EHA), Monounsaturated Fatty acids (MUFA), Omega-6, Omega-3 |
| **Neuroplasticity** | Brain Derived Neurotrophic Factor (BDNF), Tropomyosin receptor kinase B (TrkB)  TrkB ligands: Adenosine, pituitary adenylate cyclase-activating polypeptide (PACAP), anandamide (an endocannabinoid), kainite, glucocorticoids, dopamine  Synaptogenesis, Bouttons, Spines, Cortical thinning, Notch signaling pathway,  mTOR, AKT |
| **Neurotransmitters** | Serotonergic system: Tryptophan, TPH, TPH1, TPH2, 5-HT, serotonin, 5-HTT, SERT, serotonin transporter, 5-HTTLPR, 5-HT1a, 5-HT1b, 5-HT1c, 5-HT1d, 5-HT1e, 5-HT1f, 5-HT2a, 5-HT2b, 5-HT2c, 5-HT3a, 5-HT3b, 5-HT4, 5-HT5, 5-HT6, 5-HT7, MAO-A, 5-HIAA  Noradrenergic system: Tyrosine, TH (tyrosine hydroxylase), Dopamine B-hydroxylase, NE, NET, VMAT, a1- and a2- adrenoreceptors, B-adrenoreceptors, COMT, MHPG, Norepinephrine, Noradrenaline, Locus Coeruleus, Monoamine Oxidase /(MAO), Catechol o-methyltransferase/ (COMT),  Dopaminergic system: Tyrosine, DOPA, DOPA decarboxylase, DA, DAT, VMAT, Dopamine receptors D1-D4, MAO-B, HVA, DOPAC, Apomorphine  GABAergic system: Glutamic acid decarboxylase, GABA, GABA transporter, GABA A & B receptors, GABA-transaminase, Succinic semialdehyde dehydrogenase.  Glutamatergic system: Glutamine, Glutamate, Glutamate decarboxylase, NMDA, AMPA  kainate receptor, mGluR 1-8  Endogenous Opioid System/ opioid system, Beta endorphin, Mu receptor, Prodynorphin, Opioid receptors,  Endogenous Cannabinoid System, Cannabinoid receptors, CB1, CB2, anandamide, 2-arachidonoylglycerol (2-AG) |
