# Supplementary material for: Pursuing the elusive biosignature for suicide: a decennial update
Source: Mol Psychiatry. 2026 Mar 12;31(7):4029–59. doi: 10.1038/s41380-026-03507-5 (PMC13268968; doi:10.1038/s41380-026-03507-5)
Supplement: Supplementary file 2 — Supplemental Table 1 [file 41380_2026_3507_MOESM2_ESM.docx]

**Supplemental Table 1. Gene and Protein Expression Findings in Suicide Decedents in Studies with Experimental Groups < 20**

| **System** | **Author/ Year** | **Sample**   - **Source: [Bank name]** - **Toxicology: psychotropics, drugs/alcohol** | **Brain Region** | **Gene expression** | | | **Protein expression** | | **Epigenetic analysis** | **Findings** | **Comments** |
| --- | --- | --- | --- | --- | --- | --- | --- | --- | --- | --- | --- |
|  |  |  |  | **Array/ other** | **qPCR** | **RNA-seq** | **WB / ELISA** | **IHC** |  |  |  |
| Neurotransmitter system: Cannabinoid | (Erdozain et al., 2015) | 12 S (6 AUD) and 12 NS (6 AUD). + Tox; + Med.  Source: local brain collection | caudate nucleus, hippocampus and cerebellum. |  |  |  | + |  |  | No difference in overall CB1R density across groups in all tested brain regions. S showed decreased CB1R density in the caudate nucleus, not in Hippocampus or cerebellum. Functional coupling of CB1 was slightly decreased in the cerebellum of suicide samples. | Positive toxicology for cannabis was an exclusion criterion. Study focused on AUD effects in a 2-WAY ANOVA where suicide main effect was also tested. |
| Neurotransmitter system: Cannabinoid | (García-Gutiérrez et al., 2018)- | 18 S with no psychiatric diagnoses and 15 NS controls with no psychiatric diagnoses.  Tox: + / Med: +  Source: local brain collection | DLPFC (BA 9) |  | + |  | + |  |  | Study evaluated gene and protein expression of CB2r and GPR55 and found lower CB2r A isoform and GPR55 gene expression in DLPFC of S vs NS, with higher CB2r protein expression and no differences in GPR55 protein expression in S vs NS. Discrepancy between gene and protein alterations was suggested as compensatory. Proximity ligation assays showed that CB2-GPR55 heteroreceptors were markedly higher in S vs NS. in both astrocytes and neurons, but not **microglia.** S<NS in CB2Ra isoform and GPR55 gene expression, but higher CB2R protein exp. GPR55 no diff. S>NS in CB2-GPR55 hetero receptors in astrocytes and neurons | All males. Donors were drug and medication free. |
| Neurotransmitter system:  Glutamate and GABA | (Zhao et al., 2018) | 17 MDD-S, 19 MDD-NS, 12 NPC.  Tox: + / Med: +  Source: Stanley Medical Research Institute | ACC, dlPFC |  | **+** |  |  |  |  | This study tested expression of 16 glutamatergic and 16 GABAergic gene transcripts (receptors, their subunits, transporters, enzymes, and scaffolding proteins). For glutamate genes, ACC expression of all studied markers (except mGluR3) was increased in MDD-S relative to MDD-NS and NPCs; MDD-NS did not differ from NPC. In dlPFC, both MDD-S and MDD-NS showed higher expression relative to NPC, but MDD-S had reductions relative to MDD-NS. For GABA genes, expression of all markers was increased in the ACC of MDD-S relative to MDD-NS and NPCs; MDD-NS did not differ from NPC. Expression of most but not all markers was increased in dlPFC of MDD-S and MDD-NS relative to NPCs. | Some NPCs had AUD and substance abuse; one NPC took anxiolytics. |
| Neurotransmitter system: Glutamate | (Gray et al., 2015) | 34 MDD-S; 19 MDD-NS ; 32 NPSx.  Tox: + / Med: +  Source: NIMH Clinical Brain Disorders Branch Bank | mPFC (BA 9/46) |  | **+** |  |  |  |  | MDD-S had higher GRIN2B, GRIK3 and GRM2 gene expression than MDD-NS. This was true for males plus females and females alone. Only GRIK3 was sig in males alone. Higher sensitivity GluR subunits, GluN2A and GluN2B were more prevalent in female MDD-S. | Donors were not drug or med free. |
| Neurotransmitter system:  Glutamate | (Dean et al., 2016) | 10 BD (1S), 10 MDD (8S), 20 SCZ (5S), and 20 NPCs.  Tox: NA / Med: +  Source: local brain collection | Frontal pole (BA10), dlPFC (BA46), ACC (BA24), parietal cortex (BA40) | **+** |  |  | **+** |  |  | The study used in situ hybridization to assess GRIN levels and tested post synaptic density of protein 95. The level of GRIN2B subunit mRNA was increased in the parietal cortex and decreased in the dlPFC of S relative to NS, even when NPCs were excluded. Post-synaptic density of protein 95 in ACC was higher in S relative to NS, even when NPCs excluded. | S significantly younger than NS. |
| Neurotransmitter system: Glutamate and GABA | (Yin, Pantazatos, et al., 2016) | 21 MDD-S, 9 MDD-NS, 29 NPC  Tox: + / Med: +  Tox: + / Med: +  Source: local brain collection, UK Brain Expression Consortium | dlPFC |  |  | **+** |  |  |  | This study used RNA-sequencing data to examine specific glutamatergic and GABAergic gene expression and found no relationship between gene expression and suicide.  MDD-S= MDD-NS in glutamate and GABA related gene expression | Study included a component of genomic associations (detailed in Table 2) |
| Neurotransmitter system: Glutamate | (Zhao et al., 2016) | 17 MDD-S, 7 MDD-NS, and 12 NPCs  Tox: + / Meds: + Source: Stanley Medical Research Institute | dlPFC, ACC |  | **+** |  |  |  |  | This study examined expression of various components of the glutamate-glutamine cycle in two brain regions. In ACC, expression of neuronal glutamate transporters (ASCT1, EAAT3, EAAT4) and transcription of glutamine transporters (SNAT1, SNAT2) were increased in MDD-S relative to both MDD-NS and NPCs. In dlPFC, expression of ASCT1 and glial genes EAAT1, EAAT2, and GLUL was decreased in MDD-S relative to MDD-NS.  No difference in gene expression were found between violent and non-violent suicides in either brain areas. | Some NPCs with AUD and substance abuse. |
| Neurotransmitter system: Glutamate | (Dean et al., 2019) | 15 MDD patients (12S), 15 BD patients (5S), 15 SCZ PTs (6S) and 15 NPCs  Tox: NA / Meds: NA Source: local brain collection | BA24, BA46 |  |  |  | **+** |  |  | The study examined metabotropic glutamate receptor levels and found GRM2 protein expression levels were higher in S relative to NS in BA46 but not BA24; the same was true for males versus females. There was no difference in GRM2 levels between NS and NPCs in either BA24 or BA46. | No information about psychotropic medications or toxicology. |
| Neurotransmitter system: Serotonin | (Lo Vasco et al., 2015) | 28 S (20 MDD) and 18 HC  Source: Section of Legal Medicine, Padua University  Tox +/Meds+ | Cortex |  | **+** |  |  |  |  | All controls expressed 6 isoforms (PLCB1,PLCB3, PLCB4,PLCG1,PLCD3 and PLCH1) of the phosphoinositide-specific phospholipase C (PLC) enzyme genes involved in the serotonin signaling system. Suicide decedents showed reduced expression of these same isoforms, with expression varying by isoform. Some suicide decedents expressed PLCD1 and PLCD4 which were not expressed in controls. | No information on diagnoses of 8/28 S. No information about demographics (age, sex). |
| Neurotransmitter system: Serotonin | (Bani-Fatemi et al., 2017) | 9 S and 11 NS (9 with psychiatric diagnoses and 2NPCs)  Tox: NA / Meds: +  Source: Stanley Medical Research sample | DLPFC (BA46) and PFC BA9 |  |  |  |  |  | **+** | This study examined epigenetic changes in six 5-HTR2A exon I CpG sites via direct CpG methylation analysis of genomic DNA and found no significant difference in CpG methylation between suicides and controls. |  |
| Neurotransmitter system: Other | (Labonté et al., 2020) | 35 S and 16 NPCs  Tox: NA /Meds: NA Source: Douglas–Bell Canada Brain Bank | Hippocampus |  | + |  |  |  | **+** | This study tested levels of methylation and expression of the MAO-A gene. Found downregulation of MAO-A but not MAO-B expression in S. S also showed upregulation of a novel long non-coding RNA, MAO-A-Associated lncRNA (MAALIN), which lies in the intergenic region and is regulated by variable methylation of its regulatory regions in S, thus leading to variable expression. MAALIN in turn regulates MAO-A expression. | Male Caucasian sample. Patient sample with impulse-control disorders (cluster B, gambling, substance dependence). No psychiatric controls. |
| **Stress biology** | (Guintivano et al., 2014) | Cohort 1:  21 MDD-S, 8 MDD-NS, 2 S without psychiatric diagnosis, 27 NPCs.  Tox: + / Meds: +  Source: NICHD.  Cohort 2:  6 SCZ-S, 23 SCZ-NS, 13 Bipolar-S, 15 Bipolar-NS, 29 NPCs.  Tox: + / Meds: +  Source: Stanley Medical Research Institute (SMRI).  Cohort 3:  4 Bipolar-S, 8 Bipolar-NS, 12 NPCs.  Tox: + / Meds: +  Source: local brain collection | PFC |  | **+** |  |  |  | **+** | In all 3 cohorts, SKA2 gene expression reduced in suicide and associated with genetic and epigenetic variation of rs7208505, possibly mediated by long-range interaction with miR-301a. SKA2 (spindle and kinetochore associated complex subunit 2) gene encodes a scaffold protein implicated in chaperoning the glucocorticoid receptor (GR) into the nucleus. miR-301a modulates SKA2 gene expression by inhibiting CREB binding to the SKA2 promoter. | Study included 3 cohorts |
| **Stress biology** | (Pantazatos et al., 2015) | 21 MDD-S, 9  MDD-NS, 29 NPCs.  Source: local brain collection | PFC (BA9) |  |  | **+** |  |  |  | SAT1, a rate-limiting enzyme of polyamine metabolism relevant to the polyamine stress response: lower in MDD-S and MDD relative to C. no S-specific effects in SAT1 total or by any of the 10 isoforms. No differential miRNA expression between MDD-S, MDD-NS and C | Exploratory whole genome analysis of RNA-seq data was used to quantify gene expression. no AUD or SUD. |
| **Stress biology** | (Schneider et al., 2015) | 6 S (of which 1 with MDD), 6 NS NPCs  Tox: NA / Meds: NA  Source: local brain collection | Frontal cortex (BA10) |  |  |  |  |  | + | Comparison of DNA methylation patterns between suicide and controls using Illumina 450K methylation arrays revealed DNA methylation changes in a large number of genes, but no changes with large effects reaching genome- wide significance. Global methylation of all analyzed CpG sites was significantly (0.25 percentage point) lower in suicide than in control brains, whereas the vast majority (97%) of the top 1,000 differentially methylated regions (DMRs) were higher methylated (0.6 percentage point) in suicide brains. | All samples were from Caucasian males. Small sample size. One out of 6 S cases had depression; hence the effects cannot be specifically related to suicide. |
| **Stress biology** | (Zhao et al., 2015) | 17 MDD-S, 7 MSS-NS, 12 NS NPCs.  Tox: + / Meds: +  Source: Stanley Medical Research Institute. | DLPFC (B46), ACC (BA24) |  | + |  |  |  |  | In the DLPFC, no significant differences were observed between MDD-S, MDD-NS, and control subjects in any of the transcripts of the stress biology genes evaluated in the study. In the ACC, CRH transcripts were upregulated in the MDD-S patients compared to MDD-NS patients (2-fold changes) and to the control group (1.6-fold changes), while no differences were observed between MDD-NS patients and control subjects. |  |
| **Stress biology** | (Yin, Galfalvy, et al., 2016) | For gene expression:  21 MDD-S, 9 MDD-NS, 29 NPCs;  Tox: + / Meds: +  Source: local brain collection | DLPFC (BA9) |  |  | **+** |  |  |  | RNA-seq analyses for expression of FKBP5, SKA2, and glucocorticoid receptor NR3C1 revealed that one NR3C1 transcript had lower expression in suicide relative to both MDD-NS and NPC controls.  Two SNPs of SKA2 were associated with suicide but did not stay significant after adjustment for multiple testing. | All subjects were of European Ancestry. For SNP and haplotype associations of suicide with FKBP5, SKA2, NR3C1 polymorphisms  121 S, 88 NS.  Psychological autopsy |
| **Stress biology** | (Lu et al., 2017) | 17 MDD-S, 7MDD-NS, 12 NPCs;  Tox: NA / Meds: +  Source: Stanley Medical Research Institute. | DLPFC, ACC |  | + |  |  |  |  | In the ACC, male MDD-S (n = 10) had significantly higher Hypocretin (Orexin) receptor-2-mRNA levels compared with male controls (n = 8 in both MDD-NS and NPCs control groups). No differences were observed in the DLPFC. |  |
| **Stress biology** | (Romero-Pimentel et al., 2021) | 35 S (of which 17 MDD and 26 substance use, overlap not specified), 13 NS (8 of which NPCs);  Tox: + / Meds: NA  Source: local brain collection. | DLPFC (BA9) | + |  |  |  |  | + | Genome-wide DNA methylation and gene expression analyses revealed altered DNA methylation profiles at 4430 genomic regions together with 622 genes characterized by differential expression in suicide cases vs controls. Metacore-enriched analysis identified 10 genes with biological relevance to psychiatric phenotypes and suicide (*ADCY9*, *CRH*, *NFATC4*, *ABCC8*, *HMGA1*, *KAT2A*, *EPHA2*, *TRRAP*, *CD22*, and *CBLN1*) and highlighted the association that *ADCY9* has with various pathways, including neurophysiological process regulated by the CRH, and synaptic plasticity. Hypomethylation of ADCY9 was validated in targeted bisulfite sequencing. | All male subjects. Not clear if findings are specific to suicide or relate to psychiatric diagnoses. |
| **Stress biology** | (Kormos et al., 2022) | 3 S, 3 NS;  Tox: NA / Meds: NA  Source: local brain collection. | Mesencephalic ventral periaqueductal grey matter |  | + |  |  |  |  | TRPA1 mRNA expression was downregulated in the Edinger–Westphal nucleus in people who died by suicide. | Small sample size. No data on diagnoses. |
| **Stress biology** | (Slabe, Pechler, et al., 2023) | Cohort 1:  17 MDD-S, 7 MDD-NS  Tox: + / Meds: +  Source: SMRI brain bank.  Cohort 2:  13 Bipolar-S, 17 Bipolar-NS, 34 NPCs.  Tox: + / Meds: +  Source: SMRI Array Collection. | ACC and DLPFC | **+** | + |  |  |  |  | Study evaluated mRNA expression of Pituitary adenylate cyclase-activating polypeptide (PACAP) (Adcyap1) and its receptors, PAC1 (Adcyap1r1), VPAC1, VPAC2 and CD38. In ACC, PACAP-mRNA was downregulated in MDD-S and BD-S compared to MDD-NS and BD-NS, with no significant differences between S and NS in DLPFC. |  |
| **Stress Biology** | (Slabe, Balesar, Verwer, Van Heerikhuize, et al., 2023) | 19 MDD-S, 16 MDD-NS, 12 C; 13 BD-S,  17 BD-NS, 34 C  Tox: NA / Meds: +  Source: SMRI | Hypothalamic paraventricular nucleus (PVN), DLPFC, ACC. |  | **+** |  |  |  |  | mRNA expression of PACAP (Adcyap1) and its receptors, PAC1 (Adcyap1r1), VPAC1, VPAC2 and CD38 assessed by qPCR in ACC and DLPFC. ACC but not DLPFC PACAP-mRNA in MDD-S > MDD-NS and PACAP-mRNA in BD-S> BD-NS in in both MDD and BD.. |  |
| **Stress Biology** | (Slabe, Balesar, Verwer, Drevenšek, et al., 2023) | 7 Sz-S, 28 Sz-NS  34 C  Tox: NA / Meds: +  Source: SMRI | DLPFC, ACC |  | **+** |  |  |  |  | mRNA expression of PACAP (Adcyap1) and its receptors, PAC1 (Adcyap1r1), VPAC1, and VPAC2 assessed by qPCR in ACC and DLPFC. Increased PACAP, VPAC 1 and VPAC 3 (but not PAC1) gene expression in ACC of S>NS. Decreased PAC 1 (but not PACAP (Adcyap1) and its receptors, VPAC1, VPAC2) gene expression in DLPFC of S<NS. |  |
| **Neuroinflammation: microglia** | (Schnieder et al., 2014) | 11 S and 25 NS with a similar distribution of psychiatric diagnoses.  Tox: NA / Meds: NA  Source: local brain collection | Dorsal Prefrontal white matter |  |  |  |  | **+** |  | Study evaluated staining for ionized calcium-binding adapter molecule 1 (Iba-1), cluster of differentiation 68 (CD68 or macrosialin) and myelin. Activated microglia and macrophages were combined to estimate density of “activated phagocytes.” No difference in resting or activated cells densities in S vs NS. Activated microglial density greater in ventral than dorsal prefrontal white matter in S, reverse of that observed in NS. 18% greater density of Iba-1-immunoreactive perivascular cells (phagocytes) in S suggesting exchange across the BBB, may cause an inflammatory prelude to S. | Psychological Autopsy |
| **Neuroinflammation: cytokines** | (Pandey et al., 2018) | 15 SCZ-S, 24 SCZ-NS; 24 NS NPCs;  Tox: + / Meds: +  Source: local brain collection. | PFC (BA9) |  | + |  | + |  |  | No significant differences were observed in RNA or protein expression of any of the inflammatory cytokines studied between SCZ-S to SCZ-NS. Gene and protein expression of cytokines was compared across groups and revealed that mRNA and protein levels of TNF-α and IL-6 are significantly increased and those of IL-10 are significantly decreased in the PFC of SCZ patients compared to control. | Psychiatric diagnoses determined by a structured interview with a family member/ friend. |
| **Neuroinflammation: cytokines** | (Gadad et al., 2021) | 10 S from diverse diagnostic groups: 9 mood disorders (of which 2 S), 17 mood disorders with substance use disorder (of which 6 S), 12 AUD/ substance use disorder (of which 2 S), 18 NS NPC;  Tox: + / Meds: +  Source: local brain collection. | DLPFC (BA10) |  |  |  | + |  |  | The study examined the association of cytokines level (IL-6, IL-1β, BDNF, and GDNF) between postmortem plasma, CSF, and brain tissue across heterogeneous diagnostic subject groups. Brain BDNF was higher in suicide compared to non-suicide subjects. IL-6 and GDNF were significantly correlated between brain and CSF. IL-6 was significantly correlated between brain and plasma | This study did not compare differences between S and NS within diagnostic groups, hence findings may not be specific to suicide, rather to psychiatric diagnoses. |
| **Neuroinflammation: other** | (Pandey et al., 2014) | 22 MDD-S, 11 S without MDD; 12 MDD-NS; 20 NS NPCs;  Tox: + / Meds: +  Source: local brain collection. | PFC (BA9) |  | + |  | + |  |  | mRNA expression of TLR3 and TLR4 was increased in DLPFC of depressed suicide and in depressed non-suicide subjects, compared with controls. Protein expression of TLR3 and TLR4 was significantly increased in depressed suicide subjects, but not in depressed non-suicide subjects compared with controls. | Psychiatric diagnoses determined by a structured interview with a family member/ friend. |
| **Neuroinflammation: microglia** | (Brisch et al., 2017) | 9 MDD-S, 11 MDD-NS, 7 Bipolar-S, 5 Bipolar-NS, 8 SCZ-S, 10 SCZ-NS, 22 NS NPCs;  Tox: NA / Meds: +  **Source:** local brain collection | Rostral and caudal DRN |  |  |  |  | **+** |  | Study evaluated microglia identified based on HLA-DR binding and found no differences in microglia density in S vs NS, by diagnosis or comparing patients and C in rostral and caudal DRN or in any DRN subnuclei. Diminished microglia activation in MDD NS vs MDD S and C. | Subset of patients had exposure to antidepressants or anti-psychotics. No history of substance abuse. |
| **Neuroinflammation: other** | (Pandey et al., 2019) | 24 MDD-S, 11 S without MDD, 24 NS NPCs;  Tox: + / Meds: +  Source: local brain collection. | PFC (BA9) |  | + |  | + |  |  | Protein expression of TLR2, TLR3, TLR4, TLR6 and TLR10, and mRNA expression of TLR2 and TLR3 was significantly increased in the MDD-S group compared with NC group. Suicide was independently associated with greater protein expression of TLR3, TLR4, TLR6. No independent effect of suicide or any interaction between suicide and depression have been found on protein expression of other TLRs. | Some MDD-S subjects had co-morbid diagnoses.  Psychiatric diagnoses determined by a structured interview with a family member/ friend. |
| **Neuroinflammation: other** | (Schnieder et al., 2019) | 11 S and 25 NS with a similar distribution of psychiatric diagnoses.  Tox: + / Meds: +  Source: local brain collection | Dorsal Prefrontal Cortex White Matter |  |  |  |  | **+** |  | Study evaluated Immunostaining with CD163 (brown) and Glut-1 (pink) to label perivascular monocytes and blood vessels, respectively. Findings revealed that blood vessel surface area per unit of DPFWM was 10% smaller in S than in NS, but not in VPFWM or PFC. S had total vascular area density was 14% lower dorsally than ventrally; in NS the difference was 4% and not significant. Increased density of vessel-associated immune cells in S do not reflect proliferation of non-parenchymal perivascular macrophages or peripheral immune cells. Increased immune cells at the BBB and changes in vascularization suggest alterations in neurovascular unit properties in S. | 27% (n=3) S had no diagnosis. 60% (n=15) of NS had no diagnosis.  Subjects were not med or drug free |
| **Neuroinflammation: microglia** | (Petrasch-Parwez et al., 2020) | 9 S (SCZ and Bipolar), 21 NS (SCZ and Bipolar), 17 NS NPCs.  Tox: NA / Meds: NA  Source: SMRI | anterior midcingulate cortex (aMCC) |  |  |  |  | **+** |  | This study evaluated ionized calcium binding adaptor molecule 1 (Iba1)—immunohistochemistry to identify resting and activated microglia. Findings revealed that in BD, S vs NS had lower density of microglia; in SCZ, S and NS showed no difference. Pooling S, trend to less microglial density vs C, but post-hoc analysis did not confirm this. | Study evaluated SCZ and Bipolar effects on microglia. Suicide effects were evaluated in a subsample with low power. S had later illness onset. |
| **Neuroinflammation: other** | (Shinko et al., 2020) | 16 S (with mixed comorbidities in 8) and 23 NS NPCs;  Tox: NA / Meds: -  Source: local brain collection | DLPFC |  |  |  | + |  |  | Out of 15 chemokines and related proteins, 10 proteins showed significance difference in expression levels between groups. The levels of CCL1, CCL8, CCL13, CCL15, CCL17, CCL19, CCL20, CXCL11, and IL-10 were significantly decreased, whereas the IL-16 levels were significantly increased in suicide samples (corrected P’s < 0.05). | Not clear if effects are specific to suicide or to psychiatric comorbidities in suicide cases, which included mood (n=5) psychotic (n=2) and anxiety disorders (n=1), and 3 unknown. |
| **Neuroinflammation: microglia** | (Zhang et al., 2020) | 28 SCZ-S, 7 SCZ-NS, 34 NS NPCs.  Tox: NA / Meds: NA  Source: SMRI | ACC (BA 24), DLPFC (BA 46) |  | **+** |  |  |  |  | Gene expression was evaluated in DLPFC and ACC grey matter to identify expression of: astrocyte-related genes (ALDH1L1, GFAP, GLT1, GS, S100b); microglia-related genes (CD68, CX3CR1, HLA-DRA, IBA1, P2RY12, TREM2, and TSPO; oligodendrocyte-related genes (MBP, MOG, OLIG2 and PLP1). Findings- in SCZ-S vs SZ-NS DLPFC astrocytes: lower ALDH1L1 and GS; ACC astrocytes: trend for higher ALDH1L1; DLPFC microglia: no diff; ACC microglia: higher P2RY12, TREM2 and CX3CR1; DLPFC or ACC oligodendrocytes: no diff | No data about presence of drugs or medication |
| **Neuroinflammation: microglia** | (Zhang et al., 2021) | 17 MDD-S, 7 MDD-NS, 12 NS NPCs.  Tox: + / Meds: +  Source: SMRI | ACC (BA 24) /DLPFC (BA 46) |  | **+** |  |  |  |  | Study evaluated RNA for microglia related proteins (CD11B (CR3), CD45, CD68, chemokine (C-X3-C motif) receptor 1 (CX3CR1), human leukocyte antigen-DR alpha chain (HLA-DRA), ionized calcium-binding adapter molecule 1 (IBA1), purinergic receptor 12 (P2RY12), transmembrane protein 119 (TMEM119), triggering receptor expressed on myeloid cells 2 (TREM2) and translocator protein (TSPO).  There was no impact of S on any protein’s gene transcription.  In ACC, but not DLPFC, there was a trend for increased microglia gene expression of CD11B, IBA1, P2RY12 and TREM2, after correction for multiple comparisons. | Sample included subjects with AUD and substance use disorders. |
| **Neuroinflammation: other** | (Dóra et al., 2022) | 8 S without evidence for acute or chronic depression and 8 NPCs.  Tox: NA / Meds: NA  Source: local brain collection | DMPFC (BA9) |  |  | **+** |  |  |  | RNA-seq data was used to compare transcriptome of suicide to non-suicide samples. 1400 genes differed using log2FC > +-1 and adjusted p-value < 0.05 criteria between groups, 1262 downregulated and 138 upregulated in S. Transcriptomic pathways related to cytokine receptor signaling were enriched in the downregulated transcripts among S, while glutamatergic synaptic signaling transcripts were enriched among the upregulated transcripts in S. | Relatively small sample, no data on diagnoses of S, no psychiatric control. |
| **Neuroinflammation: microglia** | (Naggan et al., 2023) | 9 Bipolar-S, 6 Bipolar-NS, 12 NS NPCs.  Tox: + / Meds: +  Source: SMRI | hippocampus |  |  |  |  | **+** |  | The study conducted Immunohistochemistry and image analysis determined % of LAG3 and of MNPC II-expressing microglia (co-labeled with P2RY12) out of the total # of microglia. Study found that BD-S had greater hippocampal microglia density than BD-NS as measured by P2RY12 with or without MNPC II-staining the latter indicating activated microglia. % of LAG3-labeled microglia out of all microglia was lower in the BD-S than BD-NS. LAG-3 down-regulates microglia activation. | No differences between BD-S and BD-NS in substance use disorder severity or antipsychotics use. |
| **Neuroplasticity** | (Młyniec et al., 2014) | 17S and 6 NS sudden death controls.  Tox: + / Meds: +  Source: local brain collection | PFC (BA 10); hippocampus |  |  |  | **+** |  |  | The study evaluated protein levels of **GPR39-(Zn2+)-**sensing  receptor using by Western blot and found GPR39 receptor/b-actin ratio was lower in hippocampus (19%) and PFC (17%) of S vs NS. | Unknown diagnoses of S and NS. GPR39 relative to neuroplasticity unknown. The study also involved rodent experiments that tested BDNF. |
| **Neuroplasticity** | (Monsalve et al., 2014) | 13 S without psychiatric diagnoses, 13 NS NPCs;  Tox: + / Meds: +  Source: local brain collection | DLPFC and amygdala |  | + |  |  |  |  | Evaluation of gene expression levels of Notch signaling pathway genes revealed lower expression levels of NOTCH receptor genes in S compared to NS (in DLPFC, NOTCH2 was decreased, in amygdala, NOTCH1,3,4 were decreased). Differences were also observed in NOTCH ligands, with overall lower levels of the ligands in S vs. NS (in DLPCD, lower DLL4, JAGGED1,2; in amygdala, lower DLL1,4 and Jagged 1). DLL1 levels were higher in S vs. NS in DLPFC. There were no differences in DLL3 in either brain regions. There was also a decrease in DLK1 expression, a non-canonical NOTCH ligand in the amygdala, with no such differences in DLPFC and no differences in DLK2 expression in either brain regions. There was decreased expression of Notch dependent transcription factor HES1 in DLPFC and amygdala of S vs. NS, with no differences in other two Notch dependent transcription factors, HEY1 and HEY2. | All male samples. No control or suicide subjects met the criteria for any Psychiatric diagnosis during their lifetime, based on data collected from close relatives, forensic psychological  assessment, and clinical history. |
| **Neuroplasticity** | (Pandya et al., 2014) | 15 S with unknown diagnoses and 13 NS NPCs.  Tox: NA / Meds: NA  Source: Quebec Suicide Brain Bank. | BA 10 |  | + |  |  |  |  | This study evaluated c-Cbl (Casitas b Lymphoma ; an E3 ubiquitin-protein ligase, from a family of >700 proteins that conjugate ubiquitin to target proteins, resulting in cellular responses, including DNA repair, pro-survival signalling and protein degradation) mRNA levels and found decreased expression in S vs controls. | All males. |
| **Neuroplasticity** | (Punzi et al., 2014) | 13 SCZ violent suicide, 16 SCZ non-violent suicide, 77 NS;  Tox: NA / Meds: NA  Source: Lieber Institute for Brain Development brain repository | DLPFC |  | + |  |  |  | **+** | Study evaluated gene expression levels for MARCKS, SAT1, UBA6, PTEN, MAP3K3 and found MARCKS expression greater in violent suicide than in NS and non violent suicide. Other genes did not differ (SAT1, UBA6, PTEN, MAP3K3).  The study also tested two epigenetic analyses related to MARCKS expression and found (i) lower methylation of a MARCKS CpG island (cg24011531) in violent suicides < non violent suicide < NS, and (ii) greater expression of LOC285758, which codes a long noncoding RNA that flanks MARRCKS CpG island, in VS. | No data about presence of drugs or medication in decedents but charted impact of drugs on MARCKS and LOC285758 expression. |
| **Neuroplasticity** | (Pandey et al., 2015) | 7 S (3 with Bipolar and 4 with SCZ), 52 NS (16 BIP and 16 SCZ);  Tox: NA / Meds: +  Source: local brain collection | DLPFC (BA 9), cingulate gyrus (BA 24) and temporal cortex (BA 38). |  | + |  | + |  |  | The study evaluated gene and protein expression of GSK-3b, phosphorylated form at serine 9 position (pGSK-3-ser-9) and b-catenin. When compared within diagnostic groups (BIP or SCZ), there were no significant differences in the protein or mRNA expression of GSK-3b or b-catenin between those subjects who died by suicide and those subjects who did not die by suicide. | This study compared BIP, SCZ and NPC. Small sample size of S cases. |
| **Neuroplasticity** | (Misztak et al., 2020) | 14 S without known diagnoses and 8 NPCs.  Tox: NA / Meds: NA  Source: local brain collection | PFC (BA 10), Hippocampus |  |  |  | + |  | **+** | Reduced BDNF levels i accompanied by decrease in histone acetylation both PFC and hippocampus in S compared to NS | According to available medical history, all study subjects were not treated for any chronic central nervous system diseases. |
| **Other** | (Nishida et al., 2015) | 11 MDD-S, 13 MDD-NS; all MDD is post stroke  Tox: NA / Meds: NA  Source: Local brain collection | Brain sections of frontal and temporal lobes, basal ganglia, and midbrain |  |  |  |  | + |  | The study conducted immunohistochemical analysis of neurodegeneration markers. Argyrophilic grain disease and progressive supranuclear palsy were found significantly more frequently in suicide cases than in non-suicide cases. | All subjects had post stroke depression. |
| **Other** | (Rajkowska et al., 2015) | 15 MDD-S, 21 NS (5 of which with MDD and 16 NS NPCs).  Tox: - / Meds: +  Source: local brain collection | PFC tissue of white matter underlying BA 47, 11, 12 and ventral BA24 |  |  |  | + | + |  | No association for suicide with oligodendrocyte soma size and no differences in CNPase levels (CNP=C-type natriuretic peptide) between suicide and non-suicide MDD cases. | The study focused on MDD effects and found reduced oligodendrocyte soma size and dysregulated mRNA expression of myelin related genes in MDD cases compared to non-MDD, but overall no specific effects for suicide were detected.  Diagnoses were made based on informants and medical records. |
| **Other** | (Pantazatos et al., 2017) | 21 MDD-S, 9 MDD-NS, 29 NS NPCs.  Tox: + / Meds: +  Source: local brain collection. | DLPFC (BA 9) |  |  | + |  |  |  | This study conducted whole-exome gene and exon expression using RNA-seq data; small RNA-seq examined miRNA expression.  7 genes showed S effects (survived FDR < 0.1) (MTRNR2L8, a neuroprotective mitochondrial-derived peptide, was higher [compensatory]; the rest were lower in S). No genes showed a S-specific effect (over and above MDD). No differences in miRNA expression. | No significant effects specific to S, but not depression.  All subjects had negative toxicological screens for psychotropic medication and illicit drugs. No AUD or Drug Use Disorders. |
| **Other** | (Kouter et al., 2019) | 9 S and 9 NS NPCs;  Tox: + / Meds: +  Source: local brain collection | BA9 and hippocampus |  | + |  |  |  | + | This study conducted genome wide methylation and targeted gene expression analyses of candidate genes. S showed differential patterns of methylation in both brain regions relative to NS NPCs (defined as >25% difference in methylation). Gene ontology indicated nervous system regulation and cell structural integrity. S had higher expression of two genes, ZNF714 and NRIP3, in BA9; no differences in expression of candidate genes found in hippocampus. | All male sample. Only 12/19 subjects were included in the hippocampal analysis. No information about psychiatric diagnoses of S. |
| **Other** | (Cabello-Arreola et al., 2020) | 5 S with mood disorder and 5 NS with mood disorder;  Tox: + / Meds: +  Source: local brain collection | DLPFC (BA 9) |  |  |  | + |  |  | This study conducted a proteomic analysis to compare protein expression between S and controls. Thirty-three of the 5162 detected proteins showed significantly altered expression levels in the suicide cases and two of them after adjustment for body mass index. The top differentially expressed protein was potassium voltage-gated channel subfamily Q member 3 (KCNQ3), which also showed a trend to downregulation in Western blot (p = 0.045, Bonferroni adjusted p = 0.090). | Data driven proteomic analysis of a small sample. |
| **Other** | (Jabbi et al., 2020) | 24 MDD-S. 6 MDD-NS, 28 Bipolar-S, 6 Bipolar-NS, 33 NS NPCs.  Tox: NA / Meds: NA.  Source: NIMH Human Brain Collection Core | Anterior insular cortex |  |  | + |  |  |  | Study used RNA-sequencing to evaluate gene expression differences related to suicide followed by Weighted Gene Co-Expression Network Analysis. Compared to MDD-NS and Bipolar-NS, MDD-S and Bipolar-S had 20 under-expressed innate immune and inflammatory-cytokine (CRISPLD1, CHI3L1, P2RY6, & SECTM1); protein-protein interaction regulatory (MT1A, HILPDA, HELZ2, FOSB,FAM198A, SOCS3, & TPST1); neurodegeneration (RP11-155G14.6, SLC39A14, & SERPINA3); cellular-neurodevelopmental and transcriptional (LIMK2, SFN, & EDN3) pathway genes and uncharacterized genes/pseudogenes (MTND2P28, BAALC- AS1, RP11.420L9.5, & RP11.435J9.2). There were 4 over-expressed intracellular protein transport (TBC1D3E); inflammatory (RP11.1100L3.8); cell fate and apoptosis regulation (GZMA); and transcriptional, embryonic/forebrain cell development and defect (CTD-2207O23.3, & TDGF1); and neurodevelopmental (EDN3) pathway genes | Large sample with 52 S. |
| **Other** | (Yoshino & Dwivedi, 2020) | 15 MDD-S, 28 MDD-NS, 23 NS NPCs.  Tox: + /Meds: + Source: 2 local brain collection Alabama and Maryland | dlPFC |  | + |  |  |  |  | This study examined expression of genes associated with unfolded protein response in the endoplasmic reticulum (GRP78, GRP94, XBP-1, CHOP, ATF4C, and ATF6C). MDD-S group had a significantly elevated expression of GRP78, GRP94 and ATF4C related to NPC but not relative to MDD-NS; there was no difference between the MDD-NS and NPC groups. |  |
| **Other** | (Glavan et al., 2021) | 20 S and 7 NS  Tox: + /Meds: + Source: Douglas–Bell Canada Brain Bank | amygdala, hippocampus, prefrontal cortex and thalamus | + | + |  |  |  |  | This study conducted genome-wide investigation of the gene expression in the multiple brain areas, followed by KEGG pathway analysis. Findings included non-specific differences between S and controls including disturbed gene expression involved in antigen neutralization, autoimmunity, neural plasticity, stress response, signal transduction at the neurovascular unit. KEGG enrichment analysis indicated that suicide cases had clusters of downregulated pathways involved in antigen neutralization and autoimmune thyroid disease (amygdala and thalamus) and decreased axonal plasticity in the hippocampus, and two upregulated pathways involved in neuronal death in the hippocampus and olfactory transduction in the thalamus and the prefrontal cortex. |  |
| **Abbreviations**: Activating transcription factor (ATF), Anterior Cingulate Cortex (ACC), adrenoreceptor alpha 1a/2a, beta 1 (ADRA1A, ADRA2A, ADRB1), alcohol use disorder (AUD), aldehyde dehydrogenase-1 family, member L1 (ALDH1L1), androgen receptor (AR), antidepressant (AD), arginine vasopressin receptor-1a (AVP1a), benzodiazepines (BZD), brain-derived neurotrophic factor (BDNF), Brain Enriched Guanylate Kinase Associated (BEGAIN), Brodman area (BA), calcium/calmodulin-dependent protein kinase II alpha (CAMK2A), CAMP-response element-binding protein (CREB), Cannabinoid receptor 1 (CB1), Cannabinoid receptor 2 (CB2), catechol-O-methyltransferase (COMT), C/EBP homologous protein (CHOP), cellular RA binding protein 1, 2 (CRABP1, 2), chemokine (C-X3-C motif) receptor 1 (CX3CR1), cluster of differentiation 68 (CD68), control (c), coronin1A CORO1A, corticotropin-releasing hormone (CRH), CRH binding protein (CRHBP), 2',3'-Cyclic-nucleotide 3'-phosphodiesterase (CNPase), cytochrome P450, dentate gyrus (DG), delta like canonical Notch ligand (DLL), differentially methylated region (DMR); dopamine receptor D1 and 2 (DRD1, DRD2), dopamine transporter (DAT), dorsal anterior cingulate cortex (dACC), dorsal raphe nucleus (DRN), dorsolateral prefrontal cortex (DLPFC), early life adversity (ELA), estrogen receptor a/b (ERa/b), family 26, A1, B1, C1 (CYP26A1, B1, C1), excitatory amino acid transporter (EAAT), FK506-binding protein 51 (FKBP5), protein-coupled receptor 30 (GPR39), G protein-coupled receptor 55 (GPR55), Glial fibrillary acidic protein (GFAP), glucocorticoid receptor (GR), glucose-regulated protein (GRP), glutamate ammonia ligase (GLUL), glutamic acid decarboxylase (GAD), glutamate transporter 1 (GLT1), Glycogen Synthase Kinase 3 Beta (GSK3b), glutamine synthetase (GS), GPM6A, GPM6B, GRIK3, GRIN2B, GRM2, Hardy-Weinberg Equilibrium (HWE), heat shock protein 70 (HSP 70) and HSP 90, human leukocyte antigen-DRA (HLA-DRA), IBA1-immunoreactive (IBA1-IR), Indoleamine 2,3-dioxygenase-1 (Ido-1), immunoglobulin (Ig), interferon (IFN), interleukin-1b (IL1b), interleukin 6 (IL-6), ionized calcium-binding adapter molecule-1 (IBA1), Kyoto Encyclopedia of Genes and Genomes (KEGG), Major Depressive Disorder (MDD), psychotropic medications (Med), metabotropic glutamate receptor (mGlu), microRNA (miR), mineralocorticoid receptor (MR), minor allele frequency (MAF), monoamine oxidase A (MAOA), monoamine oxidase B (MAOB), myelin-associated glycoprotein (MAG), myelin basic protein (MBP), myelin oligodendrocyte glycoprotein (MOG), Myelin Proteolipid Protein (PLP), nerve growth factor (NGF), nerve growth factor receptor (NGFR), neural progenitor cells (NPC); neurotrophic tyrosine receptor kinase (NTRK, also known as tropomyosin-related kinases, Trk), neurotrophin-3 (NT-3), Neurogenic locus notch homolog (NOTCH), neurotrophin-4/5 (NT-4/5), neutral amino acid transporter (ASCT), Nitrous Oxide Synthase: NOS1, NOS2, NOS3, NOS1-interacting DHHC domain-containing protein with dendritic mRNA (NIDD), non-suicide (NS), not assessed (NA), non-psychiatric controls (NPC), norepinephrine transporter (NET), nuclear receptor subfamily 3 group C member 1 (NR3C1), Nucleolar organizing regions (NORs), oligodendrocyte-lineage (OL), oligodendrocyte transcription factor 2 (OLIG2), orbitofrontal cortex (OFC), plasma membrane monoamine transporter (PMAT), polygenic risk score (PGR); polymerase chain reaction (PCR), Prefrontal Cortex (PFC), purinergic receptor 12 (P2RY12), quantitative real-time polymerase chain reaction (qPCR), RARa, b, g and retinoid X receptor a, b, g (RXRa, b, g), receiver operating characteristic (ROC), retinaldehyde dehydrogenase 1,2,3 (RALDH 1,2,3), S100 calcium binding protein b (S100b), serotonin receptor (5HTR), schizophrenia (SCZ), serotonin receptor 1A and 2A (5-HT1A and 5-HT2A), serotonin transporter (SERT), Shared Genomic Segments (SGS), single nucleotide polymorphism (SNP), sodium-coupled neutral amino acid transporter (SNAT), spindle and kinetochore associated complex subunit 2 (SKA2), substance use disorder (SUD), suicide (S), suicide attempter (SA), toll like receptor (TLR), toxicology (Tox), translocator protein (TSPO), triggering receptor expressed on myeloid cells 2 (TREM2), transactivation Response RNA binding protein (TRBP), Tryptophan 2,3-dioxygenase (TDO2), tryptophan hydroxylase (TPH), tumor necrosis factor-a (TNFa), urocortin 3 (UCN3), ventrolateral prefrontal cortex (VLPFC), vesicular glutamate transporter (VGLUT), vesicular monoamine transporter (VMAT), Xbox binding protein (XBP-1). | | | | | | | | | | | |

**Supplemental References**:

Bani-Fatemi, A., Strauss, J., Zai, C., Wong, A. H. C., & De Luca, V. (2017). Multiple tissue methylation analysis of HTR2A exon i in suicidal behavior. *Psychiatric Genetics*, *27*(6), 219–224. https://doi.org/10.1097/YPG.0000000000000183

Brisch, R., Steiner, J., Mawrin, C., Krzyżanowska, M., Jankowski, Z., & Gos, T. (2017). Microglia in the dorsal raphe nucleus plays a potential role in both suicide facilitation and prevention in affective disorders. *European Archives of Psychiatry and Clinical Neuroscience*, *267*(5), 403–415. https://doi.org/10.1007/S00406-017-0774-1/TABLES/3

Cabello-Arreola, A., Ho, A. M. C., Ozerdem, A., Cuellar-Barboza, A. B., Kucuker, M. U., Heppelmann, C. J., Charlesworth, M. C., Ceylan, D., Stockmeier, C. A., Rajkowska, G., Frye, M. A., Choi, D. S., & Veldic, M. (2020). Differential Dorsolateral Prefrontal Cortex Proteomic Profiles of Suicide Victims with Mood Disorders. *Genes 2020, Vol. 11, Page 256*, *11*(3), 256. https://doi.org/10.3390/GENES11030256

Dean, B., Duncan, C., & Gibbons, A. (2019). Changes in levels of cortical metabotropic glutamate 2 receptors with gender and suicide but not psychiatric diagnoses. *Journal of Affective Disorders*, *244*, 80–84. https://doi.org/10.1016/J.JAD.2018.10.088

Dean, B., Gibbons, A. S., Boer, S., Uezato, A., Meador-Woodruff, J., Scarr, E., & McCullumsmith, R. E. (2016). Changes in cortical N-methyl-d-aspartate receptors and post-synaptic density protein 95 in schizophrenia, mood disorders and suicide. *Australian and New Zealand Journal of Psychiatry*, *50*(3), 275–283. https://doi.org/10.1177/0004867415586601/ASSET/IMAGES/LARGE/10.1177_0004867415586601-FIG4.JPEG

Dóra, F., Renner, É., Keller, D., Palkovits, M., & Dobolyi, Á. (2022). Transcriptome Profiling of the Dorsomedial Prefrontal Cortex in Suicide Victims. *International Journal of Molecular Sciences*, *23*(13), 7067. https://doi.org/10.3390/IJMS23137067/S1

Erdozain, A. M., Rubio, M., Valdizan, E. M., Pazos, A., Javier Meana, J., Fernández-Ruiz, J., Alexander, S. P. H., & Callado, L. F. (2015). The endocannabinoid system is altered in the post-mortem prefrontal cortex of alcoholic subjects. *Addiction Biology*, *20*(4), 773–783. https://doi.org/10.1111/ADB.12160

Gadad, B. S., Vargas-Medrano, J., Ramos, E. I., Najera, K., Fagan, M., Forero, A., & Thompson, P. M. (2021). Altered levels of interleukins and neurotrophic growth factors in mood disorders and suicidality: an analysis from periphery to central nervous system. *Translational Psychiatry 2021 11:1*, *11*(1), 1–11. https://doi.org/10.1038/s41398-021-01452-1

García-Gutiérrez, M. S., Navarrete, F., Navarro, G., Reyes-Resina, I., Franco, R., Lanciego, J. L., Giner, S., & Manzanares, J. (2018). Alterations in Gene and Protein Expression of Cannabinoid CB2 and GPR55 Receptors in the Dorsolateral Prefrontal Cortex of Suicide Victims. *Neurotherapeutics*, *15*(3), 796–806. https://doi.org/10.1007/S13311-018-0610-Y

Glavan, D., Gheorman, V., Gresita, A., Hermann, D. M., Udristoiu, I., & Popa-Wagner, A. (2021). Identification of transcriptome alterations in the prefrontal cortex, hippocampus, amygdala and hippocampus of suicide victims. *Scientific Reports 2021 11:1*, *11*(1), 1–15. https://doi.org/10.1038/s41598-021-98210-6

Gray, A. L., Hyde, T. M., Deep-Soboslay, A., Kleinman, J. E., & Sodhi, M. S. (2015). Sex differences in glutamate receptor gene expression in major depression and suicide. *Molecular Psychiatry 2015 20:9*, *20*(9), 1057–1068. https://doi.org/10.1038/mp.2015.91

Guintivano, J., Brown, T., Newcomer, A., Jones, M., Cox, O., Maher, B. S., Eaton, W. W., Payne, J. L., Wilcox, H. C., & Kaminsky, Z. A. (2014). Identification and replication of a combined epigenetic and genetic biomarker predicting suicide and suicidal behaviors. *American Journal of Psychiatry*, *171*(12), 1287–1296. https://doi.org/10.1176/APPI.AJP.2014.14010008/SUPPL_FILE/APPI.AJP.2014.14010008_DS001.PDF

Jabbi, M., Arasappan, D., Eickhoff, S. B., Strakowski, S. M., Nemeroff, C. B., & Hofmann, H. A. (2020). Neuro-transcriptomic signatures for mood disorder morbidity and suicide mortality. *Journal of Psychiatric Research*, *127*, 62–74. https://doi.org/10.1016/J.JPSYCHIRES.2020.05.013

Kormos, V., Kecskés, A., Farkas, J., Gaszner, T., Csernus, V., Alomari, A., Hegedüs, D., Renner, É., Palkovits, M., Zelena, D., Helyes, Z., Pintér, E., & Gaszner, B. (2022). Peptidergic neurons of the Edinger–Westphal nucleus express TRPA1 ion channel that is downregulated both upon chronic variable mild stress in male mice and in humans who died by suicide. *Journal of Psychiatry and Neuroscience*, *47*(3), E162–E175. https://doi.org/10.1503/JPN.210187/TAB-RELATED-CONTENT

Kouter, K., Zupanc, T., & Videtič Paska, A. (2019). Genome-wide DNA methylation in suicide victims revealing impact on gene expression. *Journal of Affective Disorders*, *253*, 419–425. https://doi.org/10.1016/J.JAD.2019.04.077

Labonté, B., Abdallah, K., Maussion, G., Yerko, V., Yang, J., Bittar, T., Quessy, F., Golden, S. A., Navarro, L., Checknita, D., Gigek, C., Lopez, J. P., Neve, R. L., Russo, S. J., Tremblay, R. E., Côté, G., Meaney, M. J., Mechawar, N., Nestler, E. J., & Turecki, G. (2020). Regulation of impulsive and aggressive behaviours by a novel lncRNA. *Molecular Psychiatry 2020 26:8*, *26*(8), 3751–3764. https://doi.org/10.1038/s41380-019-0637-4

Lo Vasco, V. R., Leopizzi, M., DellaRocca, C., Fais, P., Montisci, M., & Cecchetto, G. (2015). Impairment and reorganization of the phosphoinositide-specific phospholipase C enzymes in suicide brains. *Journal of Affective Disorders*, *174*, 324–328. https://doi.org/10.1016/J.JAD.2014.12.006

Lu, J., Zhao, J., Balesar, R., Fronczek, R., Zhu, Q. Bin, Wu, X. Y., Hu, S. H., Bao, A. M., & Swaab, D. F. (2017). Sexually Dimorphic Changes of Hypocretin (Orexin) in Depression. *EBioMedicine*, *18*, 311–319. https://doi.org/10.1016/J.EBIOM.2017.03.043/ASSET/9FF85529-CC2E-4E1C-BA6A-8496D5662145/MAIN.ASSETS/GR4.JPG

Misztak, P., Pańczyszyn-Trzewik, P., Nowak, G., & Sowa-Kućma, M. (2020). Epigenetic marks and their relationship with BDNF in the brain of suicide victims. *PLOS ONE*, *15*(9), e0239335. https://doi.org/10.1371/JOURNAL.PONE.0239335

Młyniec, K., Doboszewska, U., Szewczyk, B., Sowa-Kućma, M., Misztak, P., Piekoszewski, W., Trela, F., Ostachowicz, B., & Nowak, G. (2014). The involvement of the GPR39-Zn(2+)-sensing receptor in the pathophysiology of depression. Studies in rodent models and suicide victims. *Neuropharmacology*, *79*, 290–297. https://doi.org/10.1016/J.NEUROPHARM.2013.12.001

Monsalve, E. M., García-Gutiérrez, M. S., Navarrete, F., Giner, S., Laborda, J., & Manzanares, J. (2014). Abnormal expression pattern of Notch receptors, ligands, and downstream effectors in the dorsolateral prefrontal cortex and amygdala of suicidal victims. *Molecular Neurobiology*, *49*(2), 957–965. https://doi.org/10.1007/S12035-013-8570-Z/FIGURES/5

Naggan, L., Robinson, E., Dinur, E., Goldenberg, H., Kozela, E., & Yirmiya, R. (2023). Suicide in bipolar disorder patients is associated with hippocampal microglia activation and reduction of lymphocytes-activation gene 3 (LAG3) microglial checkpoint expression. *Brain, Behavior, and Immunity*, *110*, 185–194. https://doi.org/10.1016/J.BBI.2023.02.021

Nishida, N., Hata, Y., Yoshida, K., & Kinoshita, K. (2015). Neuropathologic Features of Suicide Victims Who Presented With Acute Poststroke Depression: Significance of Association With Neurodegenerative Disorders. *Journal of Neuropathology & Experimental Neurology*, *74*(5), 401–410. https://doi.org/10.1097/NEN.0000000000000184

Pandey, G. N., Rizavi, H. S., Bhaumik, R., & Ren, X. (2019). Innate immunity in the postmortem brain of depressed and suicide subjects: Role of Toll-like receptors. *Brain, Behavior, and Immunity*, *75*, 101–111. https://doi.org/10.1016/J.BBI.2018.09.024

Pandey, G. N., Rizavi, H. S., Ren, X., Bhaumik, R., & Dwivedi, Y. (2014). Toll-like receptors in the depressed and suicide brain. *Journal of Psychiatric Research*, *53*(1), 62–68. https://doi.org/10.1016/J.JPSYCHIRES.2014.01.021

Pandey, G. N., Rizavi, H. S., Tripathi, M., & Ren, X. (2015). Region-specific dysregulation of glycogen synthase kinase-3β and β-catenin in the postmortem brains of subjects with bipolar disorder and schizophrenia. *Bipolar Disorders*, *17*(2), 160–171. https://doi.org/10.1111/BDI.12228

Pandey, G. N., Rizavi, H. S., Zhang, H., & Ren, X. (2018). Abnormal gene and protein expression of inflammatory cytokines in the postmortem brain of schizophrenia patients. *Schizophrenia Research*, *192*, 247–254. https://doi.org/10.1016/J.SCHRES.2017.04.043

Pandya, C., Kutiyanawalla, A., Turecki, G., & Pillai, A. (2014). Glucocorticoid regulates TrkB protein levels via c-Cbl dependent ubiquitination: A decrease in c-Cbl mRNA in the prefrontal cortex of suicide subjects. *Psychoneuroendocrinology*, *45*, 108–118. https://doi.org/10.1016/J.PSYNEUEN.2014.03.020

Pantazatos, S. P., Andrews, S. J., Dunning-Broadbent, J., Pang, J., Huang, Y. yu, Arango, V., Nagy, P. L., & John Mann, J. (2015). Isoform-level brain expression profiling of the spermidine/spermine N1-Acetyltransferase1 (SAT1) gene in major depression and suicide. *Neurobiology of Disease*, *79*, 123–134. https://doi.org/10.1016/J.NBD.2015.04.014

Pantazatos, S. P., Huang, Y. Y., Rosoklija, G. B., Dwork, A. J., Arango, V., & Mann, J. J. (2017). Whole-transcriptome brain expression and exon-usage profiling in major depression and suicide: Evidence for altered glial, endothelial and ATPase activity. *Molecular Psychiatry*, *22*(5), 760–773. https://doi.org/10.1038/MP.2016.130;TECHMETA=38,91;SUBJMETA=1414,378,476,631,692,699;KWRD=DEPRESSION,NEUROSCIENCE

Petrasch-Parwez, E., Schöbel, A., Benali, A., Moinfar, Z., Förster, E., Brüne, M., & Juckel, G. (2020). Lateralization of increased density of Iba1-immunopositive microglial cells in the anterior midcingulate cortex of schizophrenia and bipolar disorder. *European Archives of Psychiatry and Clinical Neuroscience*, *270*(7), 819–828. https://doi.org/10.1007/S00406-020-01107-0/TABLES/2

Punzi, G., Ursini, G., Shin, J. H., Kleinman, J. E., Hyde, T. M., & Weinberger, D. R. (2014). Increased expression of MARCKS in post-mortem brain of violent suicide completers is related to transcription of a long, noncoding, antisense RNA. *Molecular Psychiatry 2014 19:10*, *19*(10), 1057–1059. https://doi.org/10.1038/mp.2014.41

Rajkowska, G., Mahajan, G., Maciag, D., Sathyanesan, M., Iyo, A. H., Moulana, M., Kyle, P. B., Woolverton, W. L., Miguel-Hidalgo, J. J., Stockmeier, C. A., & Newton, S. S. (2015). Oligodendrocyte morphometry and expression of myelin – Related mRNA in ventral prefrontal white matter in major depressive disorder. *Journal of Psychiatric Research*, *65*, 53–62. https://doi.org/10.1016/J.JPSYCHIRES.2015.04.010

Romero-Pimentel, A. L., Almeida, D., Munõz-Montero, S., Rangel, C., Mendoza-Morales, R., Gonzalez-Saenz, E. E., Nagy, C., Chen, G., Aouabed, Z., Theroux, J. F., Turecki, G., Martinez-Levy, G., Walss-Bass, C., Monroy-Jaramillo, N., Fernández-Figueroa, E. A., Gómez-Cotero, A., Garciá-Dolores, F., Morales-Marin, M. E., & Nicolini, H. (2021). Integrative DNA Methylation and Gene Expression Analysis in the Prefrontal Cortex of Mexicans Who Died by Suicide. *International Journal of Neuropsychopharmacology*, *24*(12), 935–947. https://doi.org/10.1093/IJNP/PYAB042

Schneider, E., El Hajj, N., Müller, F., Navarro, B., & Haaf, T. (2015). Epigenetic Dysregulation in the Prefrontal Cortex of Suicide Completers. *Cytogenetic and Genome Research*, *146*(1), 19–27. https://doi.org/10.1159/000435778

Schnieder, T. P., Trencevska, I., Rosoklija, G., Stankov, A., Mann, J. J., Smiley, J., & Dwork, A. J. (2014). Microglia of Prefrontal White Matter in Suicide. *Journal of Neuropathology & Experimental Neurology*, *73*(9), 880–890. https://doi.org/10.1097/NEN.0000000000000107

Schnieder, T. P., Zhou, I. D., Qin, B. M., Trencevska-Ivanovska, I., Rosoklija, G., Stankov, A., Pavlovski, G., Mann, J. J., & Dwork, A. J. (2019). Blood Vessels and Perivascular Phagocytes of Prefrontal White and Gray Matter in Suicide. *Journal of Neuropathology & Experimental Neurology*, *78*(1), 15–30. https://doi.org/10.1093/JNEN/NLY103

Shinko, Y., Otsuka, I., Okazaki, S., Horai, T., Boku, S., Takahashi, M., Ueno, Y., Sora, I., & Hishimoto, A. (2020). Chemokine alterations in the postmortem brains of suicide completers. *Journal of Psychiatric Research*, *120*, 29–33. https://doi.org/10.1016/J.JPSYCHIRES.2019.10.008

Slabe, Z., Balesar, R. A., Verwer, R. W. H., Drevenšek, G., & Swaab, D. F. (2023). Increased pituitary adenylate cyclase-activating peptide genes expression in the prefrontal cortex in schizophrenia in relation to suicide. *Frontiers in Molecular Neuroscience*, *16*, 1277958. https://doi.org/10.3389/FNMOL.2023.1277958/BIBTEX

Slabe, Z., Balesar, R. A., Verwer, R. W. H., Van Heerikhuize, J. J., Pechler, G. A., Zorović, M., Hoogendijk, W. J. G., & Swaab, D. F. (2023). Alterations in pituitary adenylate cyclase-activating polypeptide in major depressive disorder, bipolar disorder, and comorbid depression in Alzheimer’s disease in the human hypothalamus and prefrontal cortex. *Psychological Medicine*, *53*(16), 7537–7549. https://doi.org/10.1017/S0033291723001265

Slabe, Z., Pechler, G. A., van Heerikhuize, J., Samuels, B. A., Živin, M., Zorović, M., & Swaab, D. F. (2023). Increased pituitary adenylate cyclase-activating polypeptide in the central bed nucleus of the stria terminalis in mood disorders in men. *Neurobiology of Disease*, *183*, 106191. https://doi.org/10.1016/J.NBD.2023.106191

Yin, H., Galfalvy, H., Pantazatos, S. P., Huang, Y. Y., Rosoklija, G. B., Dwork, A. J., Burke, A., Arango, V., Oquendo, M. A., & Mann, J. J. (2016). GLUCOCORTICOID RECEPTOR-RELATED GENES: GENOTYPE AND BRAIN GENE EXPRESSION RELATIONSHIPS TO SUICIDE AND MAJOR DEPRESSIVE DISORDER. *Depression and Anxiety*, *33*(6), 531–540. https://doi.org/10.1002/DA.22499

Yin, H., Pantazatos, S. P., Galfalvy, H., Huang, Y. yu, Rosoklija, G. B., Dwork, A. J., Burke, A., Arango, V., Oquendo, M. A., & Mann, J. J. (2016). A pilot integrative genomics study of GABA and glutamate neurotransmitter systems in suicide, suicidal behavior, and major depressive disorder. *American Journal of Medical Genetics Part B: Neuropsychiatric Genetics*, *171*(3), 414–426. https://doi.org/10.1002/AJMG.B.32423

Yoshino, Y., & Dwivedi, Y. (2020). Elevated expression of unfolded protein response genes in the prefrontal cortex of depressed subjects: Effect of suicide. *Journal of Affective Disorders*, *262*, 229–236. https://doi.org/10.1016/J.JAD.2019.11.001

Zhang, L., Verwer, R. W. H., Lucassen, P. J., Huitinga, I., & Swaab, D. F. (2020). Prefrontal cortex alterations in glia gene expression in schizophrenia with and without suicide. *Journal of Psychiatric Research*, *121*, 31–38. https://doi.org/10.1016/J.JPSYCHIRES.2019.11.002

Zhang, L., Verwer, R. W. H., Zhao, J., Huitinga, I., Lucassen, P. J., & Swaab, D. F. (2021). Changes in glial gene expression in the prefrontal cortex in relation to major depressive disorder, suicide and psychotic features. *Journal of Affective Disorders*, *295*, 893–903. https://doi.org/10.1016/J.JAD.2021.08.098

Zhao, J., Qi, X. R., Gao, S. F., Lu, J., van Wamelen, D. J., Kamphuis, W., Bao, A. M., & Swaab, D. F. (2015). Different stress-related gene expression in depression and suicide. *Journal of Psychiatric Research*, *68*, 176–185. https://doi.org/10.1016/J.JPSYCHIRES.2015.06.010

Zhao, J., Verwer, R. W. H., Gao, S. F., Qi, X. R., Lucassen, P. J., Kessels, H. W., & Swaab, D. F. (2018). Prefrontal alterations in GABAergic and glutamatergic gene expression in relation to depression and suicide. *Journal of Psychiatric Research*, *102*, 261–274. https://doi.org/10.1016/J.JPSYCHIRES.2018.04.020

Zhao, J., Verwer, R. W. H., van Wamelen, D. J., Qi, X. R., Gao, S. F., Lucassen, P. J., & Swaab, D. F. (2016). Prefrontal changes in the glutamate-glutamine cycle and neuronal/glial glutamate transporters in depression with and without suicide. *Journal of Psychiatric Research*, *82*, 8–15. https://doi.org/10.1016/J.JPSYCHIRES.2016.06.017
