# Supplementary material for: Pursuing the elusive biosignature for suicide: a decennial update
Source: Mol Psychiatry. 2026 Mar 12;31(7):4029–59. doi: 10.1038/s41380-026-03507-5 (PMC13268968; doi:10.1038/s41380-026-03507-5)
Supplement: Supplementary file 3 — Supplemental Table 2 [file 41380_2026_3507_MOESM3_ESM.docx]

**Supplemental Table 2. Genetic Findings in Suicide Decedents in Studies with Experimental Groups < 20**

| **System** | **Author/Year** | **Sample** | **Genes/Loci** | **Sample** | **Findings** | **Comments** |
| --- | --- | --- | --- | --- | --- | --- |
| Neurotransmitter system: Serotonin | (Bani-Fatemi et al., 2017) | 13 S (4 SCZ, 5BD, 4MDD) and 13 NS (3 SCZ, 3BD, 1MDD, and 6NPCs)  Tox: NA / Meds: +  Source: Stanley Medical Research sample | C861G single nucleotide polymorphism (SNP) of HTR1B | DLPFC (BA46) and PFC BA9 | This study tested the allelic imbalance of the C861G single nucleotide polymorphism (SNP) of HTR1B in the frontal cortex of suicide victims and found no alterations in the C/G expression ratio in suicide victims compared to controls (*p* = 0.370). |  |
| **Abbreviations**: DLPFC, dorsolateral prefrontal cortex; BD, bipolar disorder; MDD, major depressive disorder; SCZ, schizophrenia; NPC, non-psychiatric controls; PFC, prefrontal cortex | | | | | | |

Supplemental Reference:

Bani-Fatemi, A., Howe, A., Zai, C., Kennedy, J. L., Vincent, J., Strauss, J., Wong, A., & De Luca, V. (2017). Differential Allelic Expression of HTR1B in Suicide Victims: Genetic and Epigenetic Effect of the Cis-Acting Variants. *Neuropsychobiology*, *74*(3), 144–149. https://doi.org/10.1159/000456010
