# Supplementary material for: Pursuing the elusive biosignature for suicide: a decennial update
Source: Mol Psychiatry. 2026 Mar 12;31(7):4029–59. doi: 10.1038/s41380-026-03507-5 (PMC13268968; doi:10.1038/s41380-026-03507-5)
Supplement: Supplementary file 4 — Supplemental Table 3 [file 41380_2026_3507_MOESM4_ESM.docx]

**Supplemental Table 3. Neurochemical, Autoradiographic, Morphological, and Other Findings in Suicide Decedents in Studies with Experimental Groups < 20**

| **System** | **Author/year** | **Sample**   - **Source: [Bank name]** - **Toxicology: psychotropics, drugs/alcohol** | **Brain Region** | **Method** | **Findings** | **Comments** |
| --- | --- | --- | --- | --- | --- | --- |
| **Neurotransmitters:**  **Dopaminergic system** | (Fitzgerald et al., 2016) | 17 S and 17 controls  Tox: + /Meds: +  Source: Allegheny County Coroner and NYC Medical Examiner | Dorsal striatum | Quantitative autoradiography of DAT (using [^3^H]mazindol), D1 ([^3^H]SCH23390), and D2 ([^3^H]sulpiride) receptor binding. | Positive correlation between D1 and D2 receptor binding in controls but not S, and between D1 and DAT in those without ELA but not with ELA. Mean DAT, D1, and D2 receptor binding comparable between S and controls, MDD-S and controls, and those with and without ELA. | 10S and 6 HC with ELA. 11/17 S with MDD; various other diagnoses also present. Some controls with Axis I diagnoses. |
| **Neurotransmitters: GABAergic system** | (Smiley et al., 2016) | 17 MDD-S, 8 SCZ-S, 6 SCZ-NS, and 20 NPCs  Tox: - / Meds: +  Source: Institute for Forensic Medicine in Skopje, Macedonia | Primary auditory cortex (A1), auditory association cortex (Tpt) | Immunolabeling of GABA interneuron subtypes including calretinin, calbindin and parvalbumin to determine cell density and size | SCZ-S had lower calretinin density in Tpt and in A1 relative to SCZ-NS. Calbindin and parvalbumin cell densities did differ between SCZ-S an SCZ-NS. Calbindin cell soma immunolabeling in Tpt was associated with suicide. MDD-S group had lower calbindin and calretinin cell densities in A1 and Tpt relative to NPCs. | All subjects were White. A few samples excluded in analyses due to poor immunolabel penetration. No MDD-NS group. |
| **Neurotransmitters: Glutamatergic system** | (Dean et al., 2016) | 10 BD PTs (1S), 10 MDD PTs (8S), 20 SCZ PTs (5S), and 20 NS NPCs.  Tox: NA / Meds: +  Source: Victorian Brain Bank Network | Frontal pole (BA10), dlPFC (BA46), ACC (BA24), and parietal cortex (BA40) | Measured levels of NMDAR using [3H]MK-801 autoradiography | [3H]MK-801 binding did not differ between S and NS. | S significantly younger than NS |
| **Neurotransmitters: Glutamatergic system** | (Mcomish et al., 2016) | 15 MDD PTs (12S), 15 BD PTs (4S), 15 SCZ PTs (6S) and 15 HCs  Tox: NA / Meds: NA  Source: Victorian Brain Bank Network | BA17 (visual cortex), BA24 (ACC) and BA46 (dlPFC) | Used [3H]LY341495 autoradiography to assess binding to metabotropic mGlu2/3 receptors | Ligand binding in all regions studied did not differ in S compared to NS. MDD group had lower ligand binding relative to HCs in BA24. | No information about psychotropic medications or toxicology. |
| **Neurotransmitters:**  **Serotonergic system** | (Bach et al., 2014) | 6 S and 8 NC NPCs.  Tox: - / Meds: -  Source: Local brain collection. | Brainstem, PFC | Measured total amount of 5-HT and 5-HIAA using high pressure liquid chromatography | Brainstem: Total 5-HT of S was 4x that of HCs; total 5-HIAA was 1.5x that of HCs. 5-HIAA:5-HT ratio was decreased in S.  PFC: No significant difference between total 5-HT or 5-HIAA between the two groups. 5-HIAA:5-HT ratio was decreased in S. | Small sample. Mostly male sample. Various diagnoses. |
| **Neurotransmitters:**  **Serotonergic system** | (Dean et al., 2014) | 16 MDD PTs (11 S), 14 BD PTs (4 S), 14 HCs  Tox: NA / Meds: +  Source: Victorian Brain Bank Network | BA24 and BA46 | Used [3H]ketanserin binding and autoradiography to measure HTR2A levels | Lower levels of [3H]ketanserin binding in BA24 but not BA46 in suicides compared non-suicides. |  |
| **Neurotransmitters:**  **Serotonergic system** | (Krzyzanowska et al., 2015) | 9 S +MDD, 18 NS + MDD, 4 S + SCZ, 13 NS + SCZ;  Tox: NA / Meds: +  Source: Magdeburg Brain Bank, Germany. | DRN | The transcriptional activity of ribosomal DNA (rDNA) in DRN neurons was evaluated by the AgNOR silver staining method in paraffin embedded brain tissue. | Suicides and non-suicides with schizophrenia did not differ. The MDD suicide subgroup had significantly decreased nuclear (U-test P value0.002) and AgNOR (U test P value0.046) compared with the non-suicide MDD subgroup. | All cases were with psychiatric conditions (MDD or SCZ). |
| **Neurotransmitters:**  **Serotonergic system** | (Rajkowska et al., 2017) | 8 MDD-S, 10 MDD-NS and 17 NS NPCs.  Tox: NA / Meds: +  Source: Cuyahoga County Medical Examiner’s Office, Cleveland, OH | OFC | Examined the length and density of axons expressing SERT using immunohistochemical labeling | There was no significant difference in the mean length of SERT-expressing axons between MDD-S and MDD-NS. | Alcohol abuse/ dependence in 2 NS NPCs. No comment on drug/alcohol toxicology. |
| **Other: Stress biology** | (Baltazar-Gaytan et al., 2019) | 12 S and 12 NS with no data on diagnoses.  Tox: NA / Meds: NA  Source: local brain collection. | Anterior- and posterior-pituitary glands | 8 pituitaries were used for radioligand and biochemical assays and 4 for stereological studies in each group. assessed NO levels by quantifying NO2- and NO3- NO end products in homogenates, zinc levels, superoxide dismutase activity, 4-hydroxy-alkenals, malondialdehyde and metallothioneins | Compared to NS, S had greater anterior pituitary weight due to more cells with no difference in posterior pituitary weight. S also had lower NO levels (loss of neuroprotection) and higher superoxide dismutase activity (which reduces NO) compared to NS. There were no differences in other measures tested. | S had MDD (n=3) ADHD (n=1) others unknown. Past year drug abuse excluded; tox unknown. |
| **Abbreviations**: 5-HT, serotonin; 5-HTR, serotonin receptor; ACC, anterior cingulate cortex; AUD, alcohol use disorder; BA, Brodman area; D1, dopamine 1 receptor; D2, dopamine 2 receptor; DAT, dopamine transporter; DRN, dorsal raphe nucleus; GFAP, Glial fibrillary acidic protein; HIAA, hydroxyindoleacetic acid; Ido-1, Indoleamine 2,3-dioxygenase-1; IFN, interferon; MDD, major depression disorder; Meds, psychotropic medications; NA, not assessed; NMDAR, NMDA receptor; NPC, non-psychiatric control; NS, non-suicide; ; OFC, orbitofrontal cortex; PFC, prefrontal cortex; PUFA, polyunsaturated fatty acid; rDNA, ribosomal DNA; S, suicide; SERT, serotonin transporter; SCZ, schizophrenia; TDO2, Tryptophan 2,3-dioxygenase; TDP-43, Transactive response DNA binding protein of 43 kDa; TNF, tumor necrosis factor; Tox, toxicology; VLPFC, ventrolateral prefrontal cortex. | | | | | | |

**Supplemental References**:

Bach, H., Huang, Y. Y., Underwood, M. D., Dwork, A. J., Mann, J. J., & Arango, V. (2014). Elevated serotonin and 5-HIAA in the brainstem and lower serotonin turnover in the prefrontal cortex of suicides. *Synapse*, *68*(3), 127–130. https://doi.org/10.1002/SYN.21695;WGROUP:STRING:PUBLICATION

Baltazar-Gaytan, E., Aguilar-Alonso, P., Brambila, E., Tendilla-Beltran, H., Vázquez-Roque, R. A., Morales-Medina, J. C., Maceda-Mártinez, N., Castro-Flores, C., Susano-Pompeyo, M., Garcés-Ramírez, L., de la Cruz, F., García-Dolores, F., & Flores, G. (2019). Increased cell number with reduced nitric oxide level and augmented superoxide dismutase activity in the anterior-pituitary region of young suicide completers. *Journal of Chemical Neuroanatomy*, *96*, 7–15. https://doi.org/10.1016/J.JCHEMNEU.2018.11.002

Dean, B., Gibbons, A. S., Boer, S., Uezato, A., Meador-Woodruff, J., Scarr, E., & McCullumsmith, R. E. (2016). Changes in cortical N-methyl-d-aspartate receptors and post-synaptic density protein 95 in schizophrenia, mood disorders and suicide. *Australian and New Zealand Journal of Psychiatry*, *50*(3), 275–283. https://doi.org/10.1177/0004867415586601/ASSET/IMAGES/LARGE/10.1177_0004867415586601-FIG4.JPEG

Dean, B., Tawadros, N., Seo, M. S., Jeon, W. J., Everall, I., Scarr, E., & Gibbons, A. (2014). Lower cortical serotonin 2A receptors in major depressive disorder, suicide and in rats after administration of imipramine. *International Journal of Neuropsychopharmacology*, *17*(6), 895–906. https://doi.org/10.1017/S1461145713001648

Fitzgerald, M. L., Kassir, S. A., Underwood, M. D., Bakalian, M. J., Mann, J. J., & Arango, V. (2016). Dysregulation of Striatal Dopamine Receptor Binding in Suicide. *Neuropsychopharmacology 2017 42:4*, *42*(4), 974–982. https://doi.org/10.1038/npp.2016.124

Krzyzanowska, M., Steiner, J., Brisch, R., Mawrin, C., Busse, S., Braun, K., Jankowski, Z., Bernstein, H. G., Bogerts, B., & Gos, T. (2015). Ribosomal DNA transcription in dorsal raphe nucleus neurons is increased in residual schizophrenia compared to depressed patients with affective disorders. *Psychiatry Research*, *230*(2), 233–241. https://doi.org/10.1016/J.PSYCHRES.2015.08.045

Mcomish, C. E., Pavey, G., Gibbons, A., Hopper, S., Udawela, M., Scarr, E., & Dean, B. (2016). Lower [3H]LY341495 binding to mGlu2/3 receptors in the anterior cingulate of subjects with major depressive disorder but not bipolar disorder or schizophrenia. *Journal of Affective Disorders*, *190*, 241–248. https://doi.org/10.1016/J.JAD.2015.10.004

Rajkowska, G., Mahajan, G., Legutko, B., Challagundla, L., Griswold, M., Albert, P. R., Daigle, M., Miguel-Hidalgo, J. J., Austin, M. C., Blakely, R. D., Steffens, D. C., & Stockmeier, C. A. (2017). Length of axons expressing the serotonin transporter in orbitofrontal cortex is lower with age in depression. *Neuroscience*, *359*, 30–39. https://doi.org/10.1016/J.NEUROSCIENCE.2017.07.006

Smiley, J. F., Hackett, T. A., Bleiwas, C., Petkova, E., Stankov, A., Mann, J. J., Rosoklija, G., & Dwork, A. J. (2016). Reduced GABA neuron density in auditory cerebral cortex of subjects with major depressive disorder. *Journal of Chemical Neuroanatomy*, *76*, 108–121. https://doi.org/10.1016/J.JCHEMNEU.2015.10.008
