# Supplementary material for: Pursuing the elusive biosignature for suicide: a decennial update
Source: Mol Psychiatry. 2026 Mar 12;31(7):4029–59. doi: 10.1038/s41380-026-03507-5 (PMC13268968; doi:10.1038/s41380-026-03507-5)
Supplement: Supplementary file 5 — Supplemental Table 4 [file 41380_2026_3507_MOESM5_ESM.docx]

**Supplemental Table 4. Findings in Non-Brain Biological Substrate of Suicide Decedents in Studies with Experimental Groups < 20**

| **System** | **Author/year** | **Sample** | **Biological Substrate** | **Method** | **Findings** | **Comments** |
| --- | --- | --- | --- | --- | --- | --- |
| **Neurotransmitters, Serotonergic system** | Neider et al., 2016) | 12 S and 87 NS with SCZ spectrum disorders  Tox: -/Meds: -  Source: University Hospital in Uppsala, Sweden | CSF | Examined CSF HIAA and HVA levels using gas chromatography-mass spectrometry | There were no differences between HIAA and HVA levels between the S and NS groups. The HVA/HIAA ratio was however significantly lower in the S group. | Four subjects had undetermined causes of death but were classified as probable suicides. |
| **Abbreviations**: BD, bipolar disorder; CMV, cytomegalo virus; CSF, cerebrospinal fluid; HIAA, hydroxyindoleacetic acid; HVA, homovanillic acid; MDD, major depressive disorder; NS, non-suicide S, suicide; SCZ, schizophrenia; | | | | | | |

**Supplemental Reference**:

Neider, D., Lindström, L. H., & Bodén, R. (2016). Risk factors for suicide among patients with schizophrenia: a cohort study focused on cerebrospinal fluid levels of homovanillic acid and 5-hydroxyindoleacetic acid. *Neuropsychiatric Disease and Treatment*, *12*, 1711–1714. https://doi.org/10.2147/NDT.S107178
